# Supplementary material for: Inhibition of DHCR24 activates LXRα to ameliorate hepatic steatosis and inflammation
Source: EMBO Mol Med. 2023 Jun 26;15(8):e16845. doi: 10.15252/emmm.202216845 (PMC10405065; doi:10.15252/emmm.202216845)
Supplement: Supplementary file 1 — Appendix [file EMMM-15-e16845-s005.pdf]

Zhou et al.

Inhibition of DHCR24 activates LXRα to ameliorate hepatic steatosis and inflammation

APPENDIX

Table of Contents

- **Appendix Figure S1. Inhibition of DHCR24 by SH42 alters liver lipid class compositions.** *Page 2*
- **Appendix Figure S2. Inhibition of DHCR24 by SH42 does not increase plasma DAG and TAG fractions.** *Page 3*
- **Appendix Figure S3. Representative gating scheme for FACS analysis in liver samples.** *Page 4*
- **Appendix Figure S4. Representative gating scheme for FACS analysis in blood samples.** *Page 4*
- **Appendix Figure Table S1. Antibodies used for immunohistochemistry.** *Page 5*
- **Appendix Figure Table S2. Antibodies used for flow cytometry.** *Page 10*
- **Appendix References.** *Page 5*

Appendix Figures

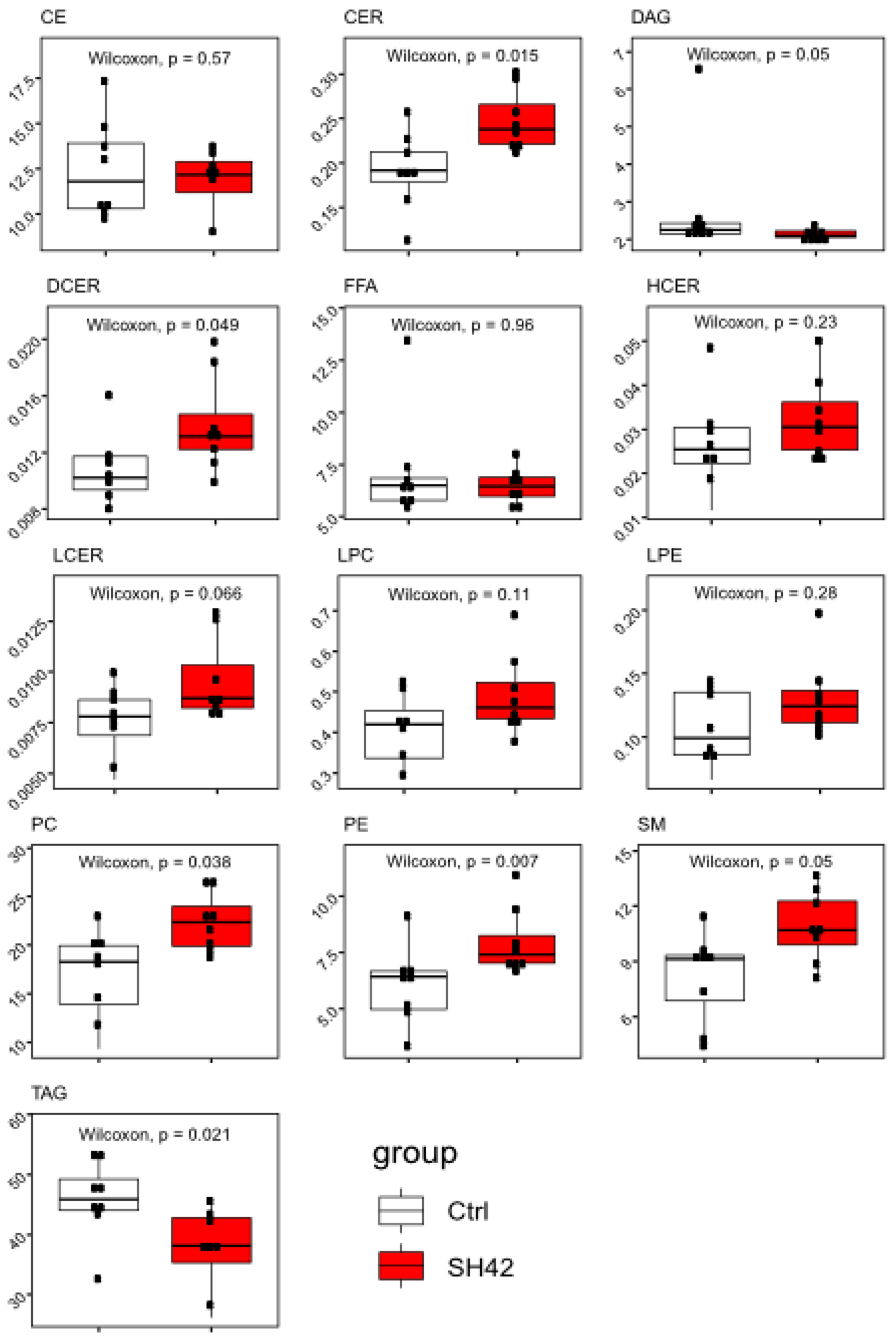

Appendix Fig. S1. Inhibition of DHCR24 by SH42 alters liver lipid class compositions.

E3L.CETP mice fed a HFCD were treated with vehicle (Ctrl) or DHCR24 inhibitor SH42 (SH42) (n= 8 mice per group). After 8 weeks of treatment, mice were killed and livers were collect to determine liver lipid class compositions. The boxplot indicates 1st quartile, median, and 3rd quartile. Differences between two groups (SH42/Ctrl) were determined using Wilcoxon rank sum test. Abbreviations: CE, cholesteryl esters; CER, ceramides; DAG, diacylglycerols; DCER, dihydroceramides; FFA, free fatty acids; HCER, hexosylceramides; LPC, lysophosphatidylcholines; LPE, lysophosphatidylethanolamines; PC, phosphatidylcholines; PE, phosphatidylethanolamines; SM, sphingomyelins; TAG, triacylglycerols.

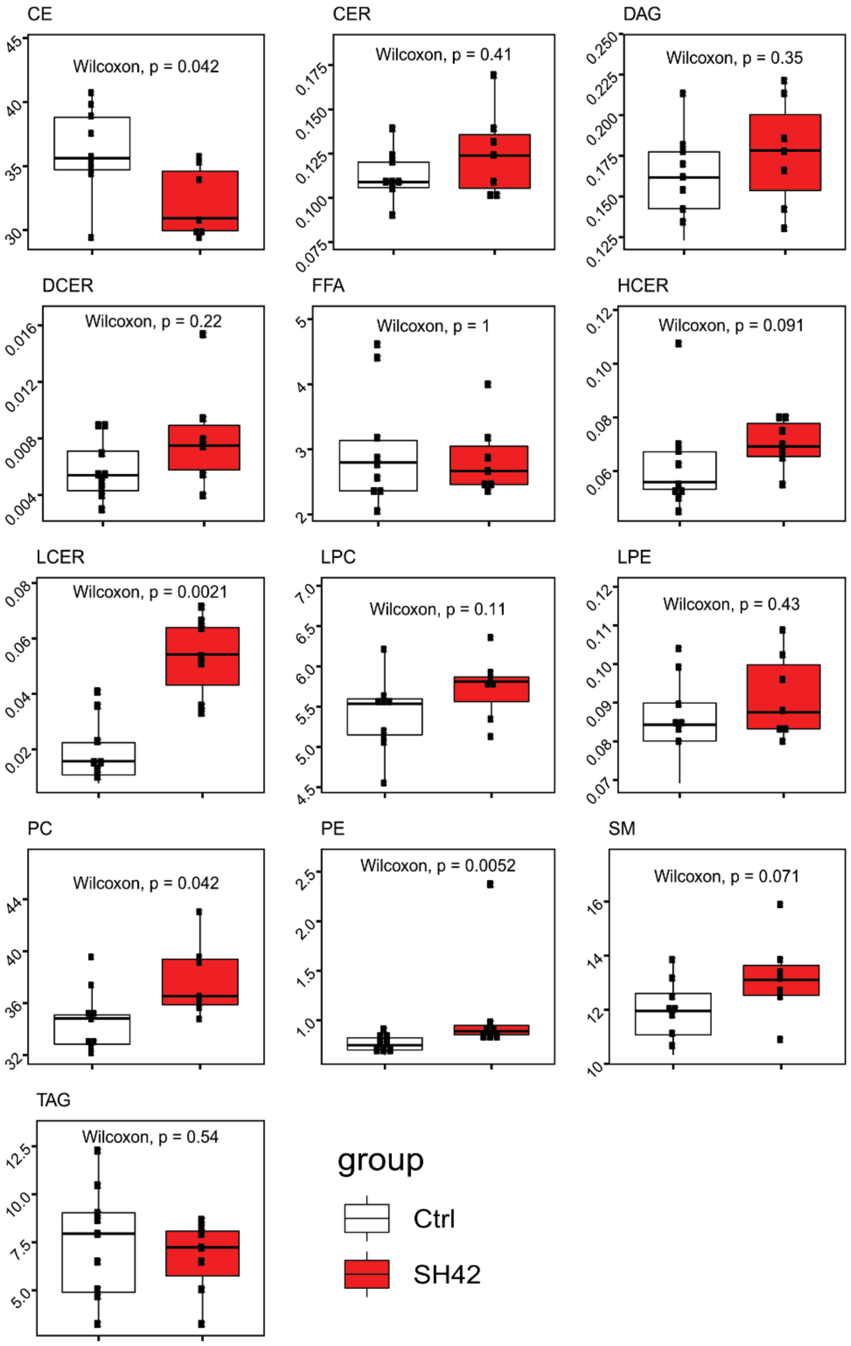

Appendix Fig. S2. Inhibition of DHCR24 by SH42 does not increase plasma DAG and TAG fractions.

E3L.CETP mice fed a HFCD were treated with vehicle (Ctrl) or DHCR24 inhibitor SH42 (SH42) (n= 8 mice per group). After 8 weeks of treatment, blood samples were collected to determine lipid class compositions. The boxplot indicates 1st quartile, median, and 3rd quartile. Differences between two groups (SH42/Ctrl) were determined using Wilcoxon rank sum test. Abbreviations: CE, cholesteryl esters; CER, ceramides; DAG, diacylglycerols; DCER, dihydroceramides; FFA, free fatty acids; HCER, hexosylceramides; LPC, lysophosphatidylcholines; LPE, lysophosphatidylethanolamines; PC, phosphatidylcholines; PE, phosphatidylethanolamines; SM, sphingomyelins; TAG, triacylglycerols.

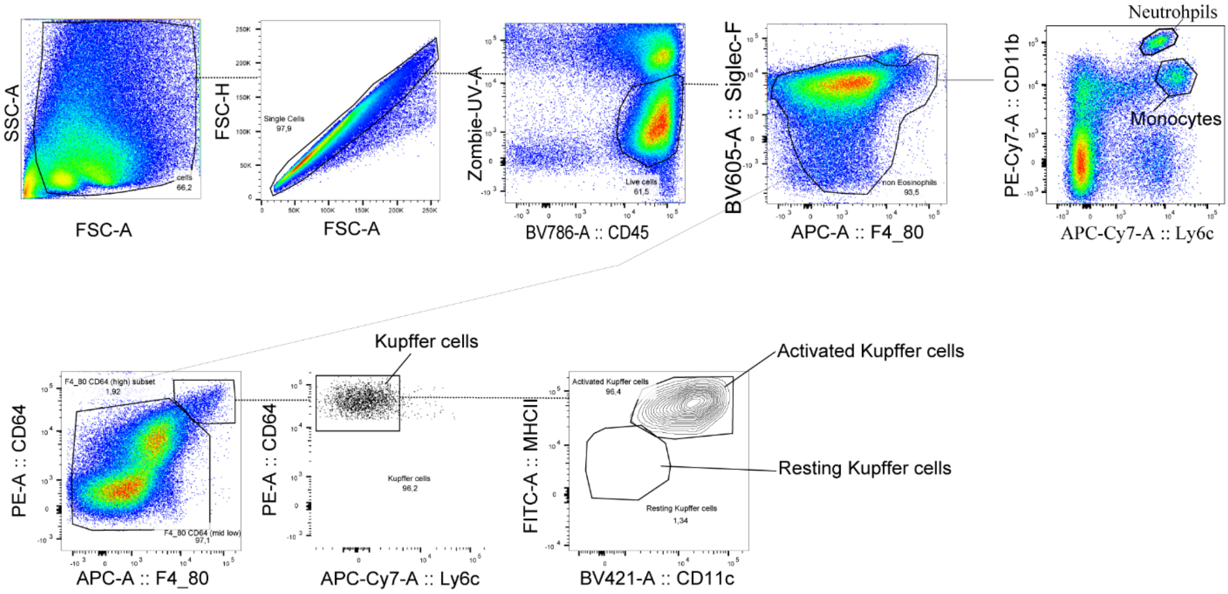

Appendix Fig. S3. Representative gating scheme for FACS analysis in liver samples.

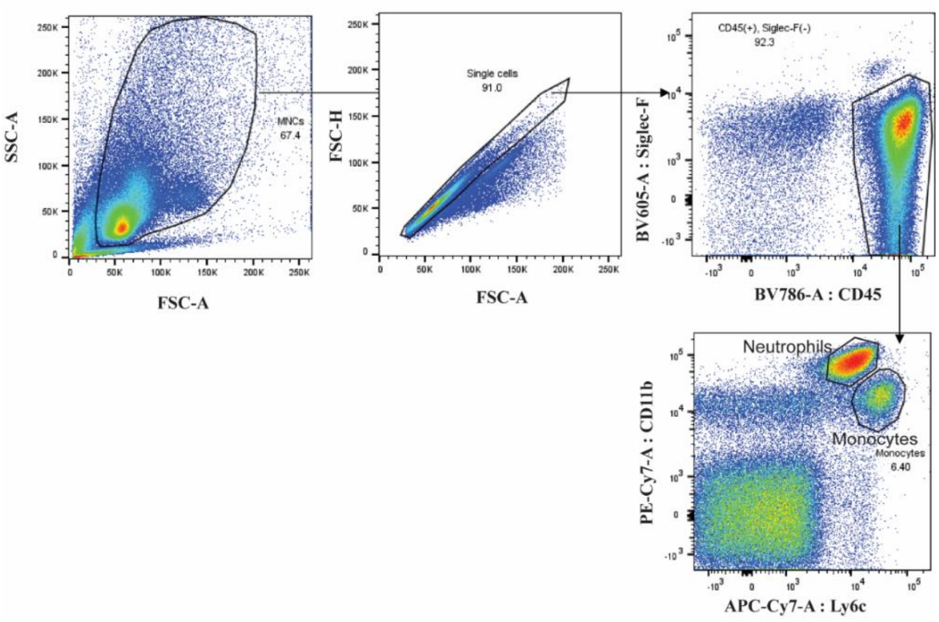

Appendix Fig. S4. Representative gating scheme for FACS analysis in blood samples.

Appendix Table S1. Antibodies used for immunohistochemistry

Target	Concentration used	Vendor	Catalog No.
F4/80	1 $\mu g \cdot mL^{-1}$	Serotec, Oxford, UK	MCA497

Appendix Table S2. Antibodies used for flow cytometry

Fluorophore	Target	Clone	Vendor
FITC	MHCII	M5/114.15.2	Biolegend
PE	CD64	X54-5/7.1	Biolegend
PE-Cy7	CD11b	M1/70	eBioscience
APC	F4/80	BM8	eBioscience
APC-Cy7	Ly6C	HK1.4	Biolegend
BV421	CD11c	N418	Biolegend
BV605	Siglec-F	E50-2440	BD Biosciences
BV785	CD45	30F11	Biolegend
