# Supplementary material for: Inhibition of DHCR24 activates LXRα to ameliorate hepatic steatosis and inflammation
Source: EMBO Mol Med. 2023 Jun 26;15(8):e16845. doi: 10.15252/emmm.202216845 (PMC10405065; doi:10.15252/emmm.202216845)
Supplement: Supplementary file 2 — Expanded View Figures PDF [file EMMM-15-e16845-s009.pdf]

Expanded View Figures

Figure EV1. Inhibition of DHCR24 by SH42 does not affect food intake, body composition, organ weight, and plasma glucose and insulin levels, while increasing plasma desmosterol levels.

A *E3L.CETP* mice fed a high-fat high-cholesterol diet (HFCD) were treated with vehicle (Ctrl) or DHCR24 inhibitor SH42 (SH42) (n = 8 mice per group).
B Food intake was measured during week 3 to 6 (n = 6 and 7 cages, respectively).
C–E Body weight was measured weekly and (D) lean body mass and (E) fat body mass were determined at the end of week 8.
F After 8 weeks of treatment, mice were killed and organs were collected and weighted.
G–I Four-hour-fasted blood samples were collected before the sacrifice to measure (G) desmosterol levels and (H) glucose and (I) insulin levels.
J The homeostatic Model Assessment for Insulin Resistance (HOMA-IR) scores were calculated.

Data information: Values are mean ± SEM. Differences between two groups (SH42/Ctrl) were determined using a nonparametric Mann–Whitney test. gWAT, gonadal white adipose tissue; iBAT, interscapular brown adipose tissue.

© 2023 The Authors

© 2023 The Authors

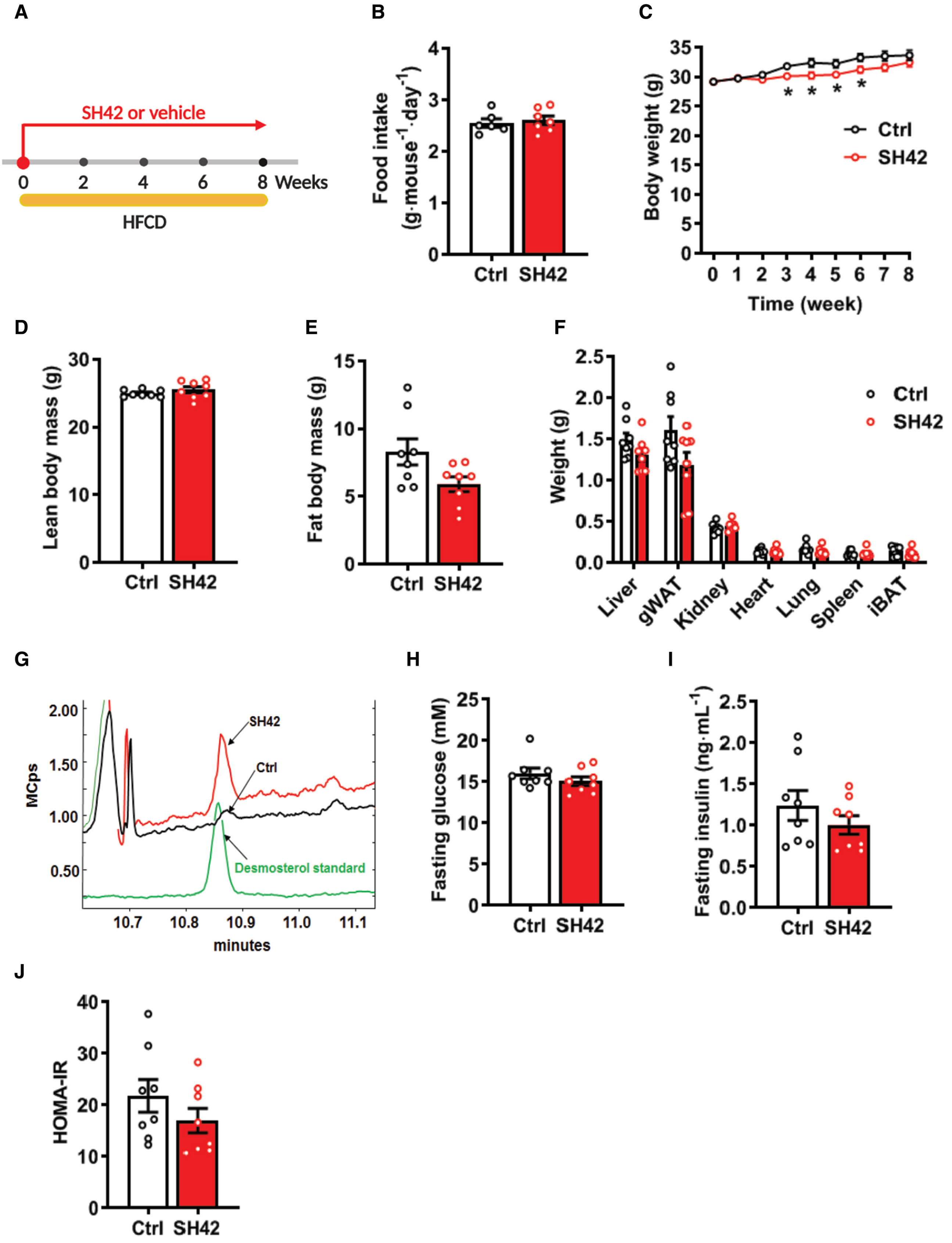
A
SH42 or vehicle
0 2 4 6 8 Weeks
HFCD
B
Food intake (g·mouse-1·day-1)
Ctrl SH42
C
Body weight (g)
Ctrl
SH42
Time (week)
D
Lean body mass (g)
Ctrl SH42
E
Fat body mass (g)
Ctrl SH42
F
Weight (g)
Ctrl
SH42
Liver gWAT Kidney Heart Lung Spleen iBAT
G
MCps
SH42
Ctrl
Desmosterol standard
minutes
H
Fasting glucose (mM)
Ctrl SH42
I
Fasting insulin (ng·mL-1)
Ctrl SH42
J
HOMA-IR
Ctrl SH42

Figure EV1.

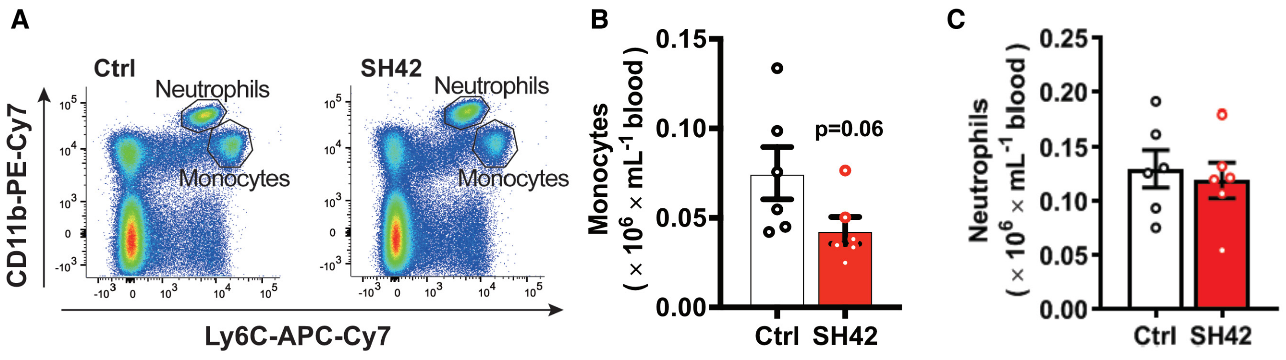

Figure EV2. Inhibition of DHCR24 by SH42 tends to reduce circulating monocytes while no effect on circulating neutrophils.

A–C *E3L.CETP* mice fed a HFCD were treated with vehicle (Ctrl) or DHCR24 inhibitor SH42 (SH42) (n = 6 mice per group). After 4 weeks of treatment, blood samples were collected to measure (A and B) monocytes and (A and C) neutrophils via flow cytometry. Values are mean ± SEM. Differences between two groups (SH42/Ctrl) were determined using a nonparametric Mann–Whitney test.

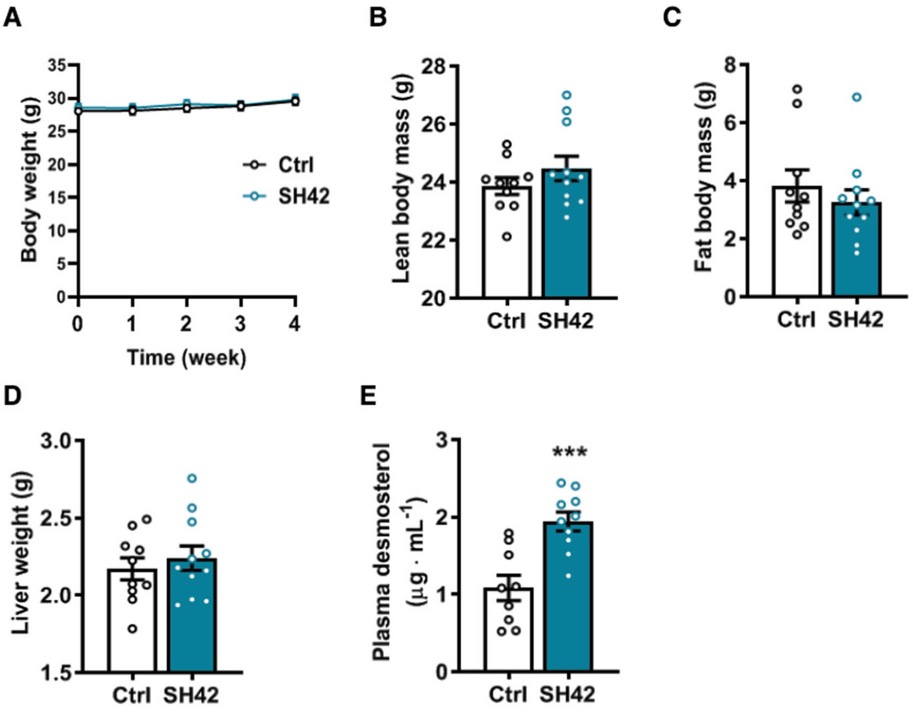

Figure EV3. Inhibition of DHCR24 by SH42 does not affect body weight, body composition or liver weight, while increasing plasma desmosterol levels in LXRα-deficient mice.

LXRα-deficient mice fed HFCD were treated with vehicle (Ctrl) or DHCR24 inhibitor SH42 (SH42) (n = 10 and 11 mice, respectively).

A Body weight was measured weekly.

B, C After 4 weeks of treatment, (B) lean body mass and (C) fat body mass were determined.

D Mice were killed and livers were collected and weighted.

E Plasma desmosterol levels were measured at the end of the experiment (n = 9 and 11 mice, respectively; one sample in control group was lost due to technical failure).

Data information: Values are mean ± SEM. Differences between two groups (SH42/Ctrl) were determined using a nonparametric Mann–Whitney test. ***$P < 0.001$ vs. ctrl.

© 2023 The Authors

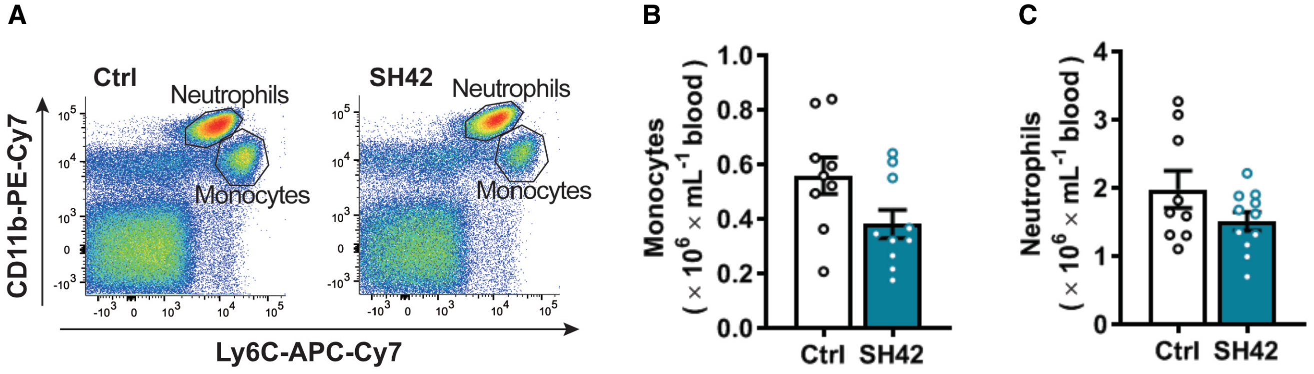

Figure EV4. Inhibition of DHCR24 by SH42 does affect circulating monocytes and neutrophils in LXRα-deficient mice.

A–C LXRα-deficient mice fed HFCD were treated with vehicle (Ctrl) or DHCR24 inhibitor SH42 (SH42) (n = 10 and 11 mice, respectively). After 4 weeks of treatment, blood samples were collected to measure (A and B) monocytes (n = 9 and 10 mice, respectively; two values were identified as outliers based on a Grubbs' test and removed from statistical analysis) and (A and C) neutrophils were determined (n = 9 and 11 mice, respectively; one value was identified as an outlier based on a Grubbs' test and removed from statistical analysis) via flow cytometry analysis. Values are mean ± SEM. Differences between two groups (SH42/Ctrl) were determined using a nonparametric Mann–Whitney test.

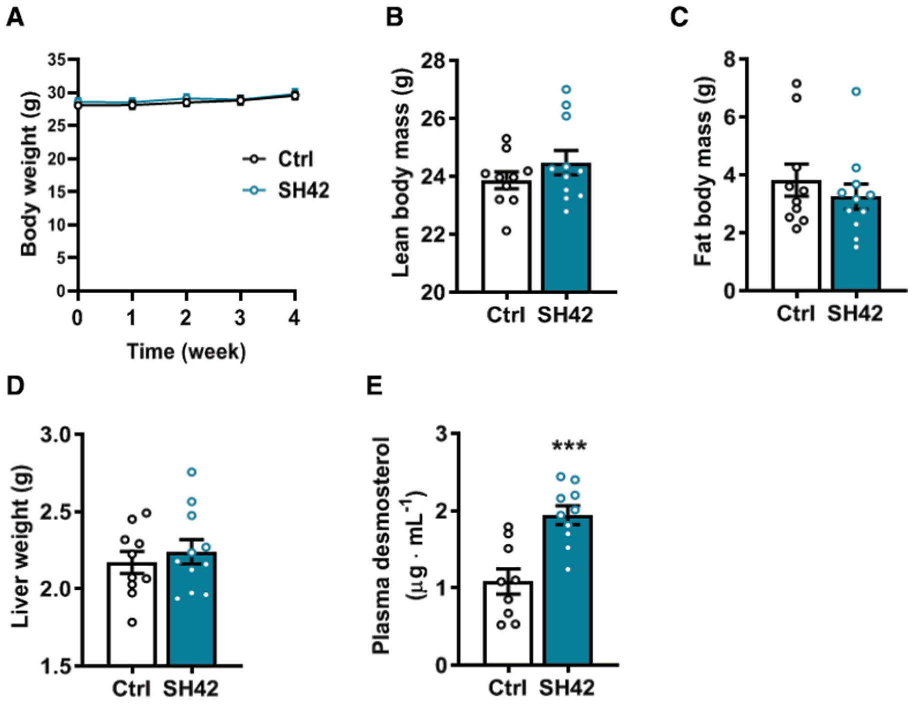

Figure EV5. Inhibition of DHCR24 by SH42 increases plasma desmosterol and does not influence body and liver weight after 10 weeks of HFCD treatment.

E3LCETP mice were fed with a HFCD for 10 weeks first and then treated with vehicle (Ctrl) or DHCR24 inhibitor SH42 (SH42) (n = 10 and 9 mice, respectively) for additional 8 weeks.

A Body weight was measured at indicated time points.

B, C After the 18 weeks, (B) lean and (C) fat body mass was evaluated.

D, E Mice were killed and (D) liver weight and (E) plasma desmosterol levels were measured.

Data information: Values are mean ± SEM. Differences between two groups (SH42/Ctrl) were determined using a nonparametric Mann–Whitney test. ***$P < 0.001$ vs. control (ctrl).

© 2023 The Authors
