# Supplementary material for: Inhibition of DHCR24 activates LXRα to ameliorate hepatic steatosis and inflammation
Source: EMBO Mol Med. 2023 Jun 26;15(8):e16845. doi: 10.15252/emmm.202216845 (PMC10405065; doi:10.15252/emmm.202216845)
Supplement: Supplementary file 3 — PDF+ [file EMMM-15-e16845-s007.pdf]

SOURCE DATA | TRANSPARENT PROCESS | OPEN ACCESS

EMBO Molecular Medicine

Article

Inhibition of DHCR24 activates LXRα to ameliorate hepatic steatosis and inflammation

Enchen Zhou[1,2], Xiaoke Ge[1], Hiroyuki Nakashima[1], Rumei Li[3], Hendrik J P van der Zande[4], Cong Liu[1], Zhuang Li[1], Christoph Müller[5], Franz Bracher[5], Yassene Mohammed[6], Jan Freark de Boer[3,7], Folkert Kuipers[3,7], Bruno Guigas[4], Christopher K Glass[2], Patrick C N Rensen[1,8], Martin Giera[6] & Yanan Wang[1,8,*]

Abstract

Liver X receptor (LXR) agonism has theoretical potential for treating NAFLD/NASH, but synthetic agonists induce hyperlipidemia in preclinical models. Desmosterol, which is converted by Δ24-dehydrocholesterol reductase (DHCR24) into cholesterol, is a potent endogenous LXR agonist with anti-inflammatory properties. We aimed to investigate the effects of DHCR24 inhibition on NAFLD/NASH development. Here, by using APOE*3-Leiden.CETP mice, a well-established translational model that develops diet-induced human-like NAFLD/NASH characteristics, we report that SH42, a published DHCR24 inhibitor, markedly increases desmosterol levels in liver and plasma, reduces hepatic lipid content and the steatosis score, and decreases plasma fatty acid and cholesteryl ester concentrations. Flow cytometry showed that SH42 decreases liver inflammation by preventing Kupffer cell activation and monocyte infiltration. LXRα deficiency completely abolishes these beneficial effects of SH42. Together, the inhibition of DHCR24 by SH42 prevents diet-induced hepatic steatosis and inflammation in a strictly LXRα-dependent manner without causing hyperlipidemia. Finally, we also showed that SH42 treatment decreased liver collagen content and plasma alanine transaminase levels in an established NAFLD model. In conclusion, we anticipate that pharmacological DHCR24 inhibition may represent a novel therapeutic strategy for treatment of NAFLD/NASH.

Keywords desmosterol; Kupffer cell; liver X receptor; nonalcoholic steatohepatitis; Δ24-dehydrocholesterol reductase
Subject Categories Digestive System; Metabolism

DOI 10.15252/emmm.202216845 | Received 7 October 2022 | Revised 6 June 2023 | Accepted 7 June 2023 | Published online 26 June 2023
EMBO Mol Med (2023) 15: e16845

Introduction

Nonalcoholic fatty liver disease (NAFLD) comprises a spectrum of diseases ranging from simple hepatic steatosis to nonalcoholic steatohepatitis (NASH). The latter is characterized by steatosis, hepatocellular damage, and inflammatory cell infiltration with or without fibrosis. Hepatic steatosis is defined as the accumulation of primarily neutral lipids such as triacylglycerols (TAG) and cholesteryl esters (CE) in the form of lipid droplets within hepatocytes, as well as nonparenchymal liver cells, including Kupffer cells (KCs; Berlanga *et al*, 2014). Hepatic steatosis results from an imbalance between lipid uptake, synthesis, secretion, and lipolysis and is usually associated with dyslipidemia, insulin resistance, hypertension, type 2 diabetes, and obesity (Chalasani *et al*, 2018). The essential trigger for the transition of simple steatosis to NASH is not yet completely elucidated. Despite the high global prevalence of NAFLD (32.4%) in the general population (Riazi *et al*, 2022), no FDA-approved medication is available yet for the treatment of NAFLD/NASH. In fact, lifestyle adjustment is still the main clinical intervention.

Inflammation contributes to the transition of simple steatosis to NASH and liver-resident KCs play a pivotal role in NAFLD/NASH pathogenesis (Baffy, 2009). Once activated during NAFLD progression, KCs produce proinflammatory factors, such as monocyte chemoattractant protein 1 (MCP-1) and tumor necrosis factor

1 Department of Medicine, Division of Endocrinology, and Einthoven Laboratory for Experimental Vascular Medicine, Leiden University Medical Center, Leiden, The Netherlands
2 Department of Cellular and Molecular Medicine and Department of Medicine, University of California San Diego, La Jolla, CA, USA
3 Department of Pediatrics, University of Groningen, University Medical Center Groningen, Groningen, The Netherlands
4 Department of Parasitology, Leiden University Medical Center, Leiden, The Netherlands
5 Department of Pharmacy, Center for Drug Research, Ludwig Maximilians University, Munich, Germany
6 The Center for Proteomics and Metabolomics, Leiden University Medical Center, Leiden, The Netherlands
7 Department of Laboratory Medicine, University of Groningen, University Medical Center Groningen, Groningen, The Netherlands
8 Med-X Institute, Center for Immunological and Metabolic Diseases, and Department of Endocrinology, First Affiliated Hospital of Xi'an Jiaotong University, Xi'an Jiaotong University, Xi'an, China
*Corresponding author. Tel: +86 29 85323338; E-mail: yanan.wang@xjtu.edu.cn

© 2023 The Authors. Published under the terms of the CC BY 4.0 license

(TNF), leading to increased hepatic monocyte recruitment and inhibition of canonical insulin signaling, further aggravating liver injury and steatosis (Aparicio-Vergara *et al*, 2013; Wandrer *et al*, 2020). In addition, KC-derived pro-fibrinogenic factors increase collagen production by hepatic stellate cells, generating a vicious circle that exacerbates NAFLD and inflammation, and derive progression towards NASH (Tosello-Trampont *et al*, 2012; Schuster *et al*, 2018). Thereby, selective depletion of KCs from the liver alleviates hepatocellular damage and prevents diet-induced hepatic steatosis and insulin resistance (Huang *et al*, 2010; Lanthier *et al*, 2011). Furthermore, consumption of a cholesterol-rich diet causes cholesterol accumulation in KCs to yield foamy inflammatory KCs, which directly contribute to liver inflammation (Bieghs *et al*, 2013). Recently, we and others showed that NAFLD/NASH impaired KC self-renewal and induce KC death, thus reducing embryonically-derived liver-resident KCs (Remmerie *et al*, 2020; Seidman *et al*, 2020; Tran *et al*, 2020). This KC niche is replenished through recruitment, differentiation, and proliferation of monocyte-derived macrophages, which are, however, reported to be more inflammatory than KCs and to contribute to the aggravation of liver inflammation and damage (Remmerie *et al*, 2020; Tran *et al*, 2020).

Liver X receptors (LXRs), that is, the LXRα and LXRβ isoform, are essential (oxy)sterol-activated transcription factors involved in lipid metabolism and immune responses (Bensinger & Tontonoz, 2008; Ito *et al*, 2015). LXRα is abundantly expressed in liver, adipose tissue, and macrophages, while LXRβ is ubiquitously expressed (Prufer & Boudreaux, 2007). In macrophages, LXRα directly promotes reverse cholesterol transport via up-regulating ATP binding cassette (ABC) A1 (Venkateswaran *et al*, 2000). Thus, LXRα-deficiency impairs cholesterol efflux and is associated with increased atherosclerosis (Bischoff *et al*, 2010; Ishibashi *et al*, 2013). Simultaneously, LXRs exert anti-inflammatory effects in immune cells (Joseph *et al*, 2004), suppress KC activation, and protect against hepatic injury (Wang *et al*, 2006). In addition, LXRs promote the formation of long-chain polyunsaturated fatty acids (PUFAs), for example, eicosapentaenoic acid and docosahexaenoic acid, which have anti-inflammatory activities (Li *et al*, 2013; Korner *et al*, 2019). The simultaneous regulation of lipid metabolism and inflammation serves LXRs as a potential drug target for NAFLD/NASH treatment (Bensinger & Tontonoz, 2008; Ito *et al*, 2015). However, at present no selective LXR agonists exist for the clinical treatment of NAFLD/NASH. This is mainly due to the unfavorable effects of pharmacological LXR activation on sterol regulatory element-binding proteins (SREBPs)-induced lipogenesis, resulting in elevated atherogenic low-density lipoprotein (LDL) cholesterol and triglycerides. Therefore, rather than alleviating liver lipid levels, most synthetic LXR agonists actually cause hepatic steatosis and hypertriglyceridemia (Schultz *et al*, 2000; Grefhorst *et al*, 2002). Moreover, some synthetic LXR agonists have been reported to cause neutropenia due to the downregulation of neutrophil production in bone marrow and stimulation of their clearance by macrophages within peripheral tissues (Kirchgessner *et al*, 2016). Taken together, a successful drug candidate for the treatment of NASH through LXR activation, should be LXR selective, not induce sterol regulatory element-binding transcription factor 1 (*Srebf1*), and not cause neutropenia.

Previously, we showed that desmosterol increases LXR target genes while inhibiting SREBP target genes in macrophages (Spann *et al*, 2012; Muse *et al*, 2018). In addition, we reported that synthetic SH42 inhibits Δ24-dehydrocholesterol reductase (DHCR24), an important enzyme intertwining the Bloch and Kandutsch-Russell pathways of distal cholesterol biosynthesis, and causes accumulation of desmosterol (Nes, 2011). Accordingly, we have recently reported that inhibition of DHCR24 induces LXR activation through the accumulation of desmosterol, promoting the resolution of inflammation without affecting *Srebf1c* expression in macrophages (Muller *et al*, 2017; Korner *et al*, 2019). Taken together, we hypothesized that inhibiting DHCR24 increases desmosterol levels and subsequently induces LXR activation in KCs, thereby suppressing inflammation, without inducing lipid synthesis via *Srebf1*. Using our published DHCR24 inhibitor SH42 (Muller *et al*, 2017), we aimed to investigate the potential therapeutic effects of DHCR24 inhibition on diet-induced NAFLD development in APOE*3-Leiden.CETP mice, a well-established humanized mouse model for the study of (cardio) metabolic diseases.

Results

Inhibition of DHCR24 by SH42 markedly increases liver desmosterol levels and ameliorates hepatic steatosis

To assess the effects of DHCR24 inhibition on hepatic steatosis, *E3L.CETP* mice were fed a HFCD while being treated with vehicle or the synthetic DCHR24 inhibitor SH42 (Muller *et al*, 2017; Korner *et al*, 2019) for a period of 8 weeks (Fig EV1A). SH42 treatment did not affect food intake (Fig EV1B), while temporarily preventing HFCD-induced body weight gain as compared to the control group (Fig EV1C). After 8 weeks of treatment, body weight and body composition, that is, lean body mass (Fig EV1D) and fat body mass (Fig EV1E) of SH42-treated mice, were comparable to that of the control group. In addition, the weight of various tissues (i.e., liver, white adipose tissue, kidney, heart, lung, spleen, and brown adipose tissue) was unchanged by SH42 treatment (Fig EV1F). As anticipated, SH42 markedly increased hepatic desmosterol levels (10-fold, Figs 1A and EV1G).

Hepatic steatosis was evaluated by HE staining and scored as described previously (Liang *et al*, 2014). As compared to the control treatment, SH42 treatment ameliorated diet-induced hepatic steatosis (Fig 1B), as evident by a clear reduction of the hepatic steatosis score (−58%, Fig 1C), and liver lipid area stained by Oil Red O (−53%, Fig 1B and D). We next analyzed liver lipid profiles by comprehensive lipidomic analysis. Firstly, we observed a clear alteration in the lipid class composition after SH42 treatment (Fig 1E). Specifically, SH42 treatment caused a relative reduction of TAG (−21%) and DAG (−22%), accompanied by a relative increase of the other lipid classes, including CER, PC, PE, and SM (Fig 1E and Appendix Fig S1). Volcano plot analysis of the lipid species concentrations of different lipidomes revealed the majority of significantly altered lipid species by SH42 treatment were down-regulated, and most of them were TAGs (Fig 1F). Consistently, SH42 tended to decrease hepatic concentrations of triacylglycerides (TAG; −39%; $P = 0.06$, $P\_adj = 0.08$, Fig 1G) and diacylglycerides (DAG; −20%; $P = 0.08$, $P\_adj = 0.08$, Fig 1H). In addition, SH42 treatment reduced hepatic free fatty acids (FFA; −16%; $P = 0.02$, $P\_adj = 0.08$, Fig 1I) and cholesterol ester (CE) levels (−25%; $P = 0.08$, $P\_adj = 0.08$, Fig 1J). Despite these strong effects on hepatic lipid content, SH42 did not

 © 2023 The Authors

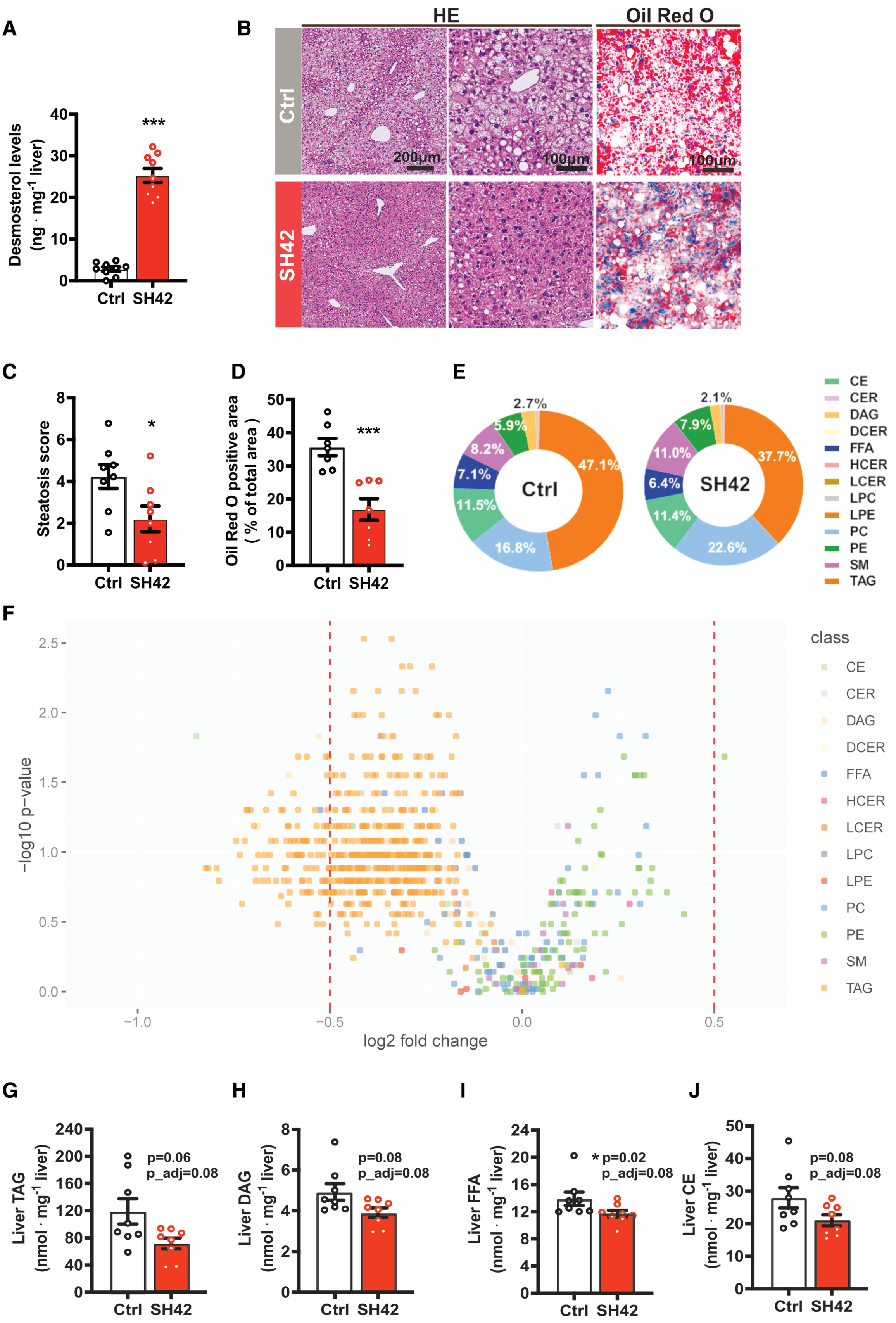

A
Desmosterol levels (ng · mg-1 liver)
Ctrl SH42
***
B
HE
Oil Red O
Ctrl
SH42
200µm
100µm
100µm
C
Steatosis score
*
D
Oil Red O positive area (% of total area)
***
E
Ctrl
SH42
CE
CER
DAG
DCER
FFA
HCER
LCER
LPC
LPE
PC
PE
SM
TAG
F
class
−log10 p−value
log2 fold change
G
Liver TAG (nmol · mg-1 liver)
p=0.06
p_adj=0.08
H
Liver DAG (nmol · mg-1 liver)
p=0.08
p_adj=0.08
I
Liver FFA (nmol · mg-1 liver)
* p=0.02
p_adj=0.08
J
Liver CE (nmol · mg-1 liver)
p=0.08
p_adj=0.08

Figure 1.

Figure 1. Inhibition of DHCR24 by SH42 markedly increases liver desmosterol and ameliorates hepatic steatosis.

E3LCETP mice fed a high-fat high-cholesterol diet (HFCD) were treated with vehicle (Ctrl) or DHCR24 inhibitor SH42 (SH42) (n = 8 mice per group).

A, B After 8 weeks of treatment, mice were sacrificed and livers were collected (A) to measure desmosterol levels, and (B) to stain with hematoxylin and eosin (HE) and Oil Red O.

C, D Hepatic steatosis was scored using the HE-stained slides and (D) lipid-positive area was quantified using the Oil Red O stained slides (n = 7 mice per group; two values were identified as outliers based on a Grubbs' test and removed from statistical analysis).

E Liver lipid classes were analyzed (Appendix Fig S1), and the relative average abundance of lipid classes in each group are depicted as pie charts.

F Volcano plot analysis of liver lipid species concentrations is shown.

G–J Hepatic (G) TAG, (H) DAG, (I) FFA and (J) CE lipid class concentrations were summarized. Benjamini–Hochberg correction was used for multiple hypothesis testing and Benjamini–Hochberg adjusted P-values are presented (P_adj).

Data information: Values are mean ± SEM. Differences between two groups (SH42/Ctrl) were determined using a Mann–Whitney test. *P < 0.05, ***P < 0.001 vs. control (ctrl). CE, cholesteryl esters; CER, ceramides; DAG, diacylglycerols; DCER, dihydroceramides; FFA, free fatty acids; HCER, hexosylceramides; LPC, lysophosphatidylcholines; LPE, lysophosphatidylethanolamines; PC, phosphatidylcholines; PE, phosphatidylethanolamines; SM, sphingomyelins; TAG, triacylglycerols. Scale bar: 100 or 200 μm as indicated.

Source data are available online for this figure.

affect fasting plasma glucose, insulin, or homeostatic Model Assessment for Insulin Resistance (HOMA-IR) scores (Fig EV1H–J). Taken together, inhibition of DHCR24 by SH42 markedly increases liver desmosterol levels, accompanied by amelioration of diet-induced hepatic steatosis without marked effects on body composition and glucose homeostasis.

Inhibition of DHCR24 by SH42 prevents Kupffer cell activation and reduces immune cell infiltration into the liver

We next evaluated the effect of DHCR24 inhibition on the hepatic inflammation in mice treated with SH42 or vehicle for 8 weeks by immunohistochemistry. SH42 treatment significantly reduced the hepatic F4/80 content (−29%, Fig 2A and B), as well as the number of hepatic crown-like structures of macrophages surrounding dying hepatocytes (−79%, Fig 2C), which is a prominent feature of NASH development (Itoh *et al*, 2013).

Flow cytometry on MACS-purified hepatic leukocytes revealed that 4 weeks of SH42 treatment tended to decrease total hepatic leukocytes (−37%; P = 0.09, Fig 2D). Given the critical role of KCs in the progression of NASH (Huang *et al*, 2010; Lanthier *et al*, 2011), we next explored the effects of DHCR24 inhibition on KCs. We observed a significant increase in total KCs upon SH42 treatment (+44%, Fig 2E and F). Although SH42 did not significantly reduce MHCII^{+}/CD11c^{+}-activated KCs (Fig 2G and H), MHCII^{-}/CD11c^{-} resting KCs were significantly increased (+21%, Fig 2G and I), indicative of prevented KC activation. In addition, SH42 treatment reduced monocytes in both liver (−79%, Fig 2J) and blood (−43%; P = 0.06, Fig EV2A and B), and decreased hepatic neutrophils (−50%, Fig 2K) without affecting circulating neutrophils (Fig EV2A and C). Together, these data indicate that SH42 treatment prevents KC activation, limits hepatic immune cell recruitment, and dampens hepatic inflammation.

Inhibition of DHCR24 by SH42 does not increase circulating lipids

Consistent with the potent increase in hepatic desmosterol levels, SH42 also markedly increased plasma desmosterol levels from undetectable levels (< 0.5 μg ml^{-1}) to 3.1 ± 0.4 μg ml^{-1} (Fig 3A). Since synthetic LXR agonists usually induce lipogenesis and hypertriglyceridemia as unwanted effects (Grefhorst *et al*, 2002; Kirchgessner *et al*, 2016), we next determined the effect of 8 weeks of SH42 treatment on circulating lipid levels using quantitative comprehensive lipidomic analysis. Analysis of the plasma lipidome revealed that SH42 treatment relatively decreased circulating CE while relatively increasing lactosylceramides (LCER), phosphatidylcholine (PC), and phosphatidylethanolamine (PE; Appendix Fig S2). With respect to absolute lipid concentrations, SH42 did not affect plasma levels of total TAG (Fig 3B) and DAG (Fig 3C), while significantly decreasing plasma levels of FFA (−16%, Fig 3D) and CE (−24%; P = 0.08, Fig 3E). These data imply that inhibition of DHCR24 by SH42 increases plasma desmosterol levels and decreases FFA and CE levels, importantly without inducing hypertriglyceridemia.

The therapeutic effects of DHCR24 inhibition by SH42 on hepatic steatosis are strictly dependent on LXRα

Based on previous reports (Spann *et al*, 2012; Muse *et al*, 2018; Korner *et al*, 2019), we hypothesized that the therapeutic effects of SH42 were attributed to LXR activation triggered by increased desmosterol levels as a result of DHCR24 inhibition. Since LXRα plays a crucial role in both lipid metabolism (Peet *et al*, 1998) and macrophage/KC homeostasis (Bischoff *et al*, 2010; Ishibashi *et al*, 2013; Endo-Umeda *et al*, 2018), we next evaluated the effects of 4 weeks of SH42 treatment in HFCD-fed LXRα-deficient mice to evaluate the importance of LXRα in the observed protective effects of SH42 in ameliorating hepatic steatosis and inflammation. SH42 treatment had no effect on body weight (Fig EV3A), body composition (Fig EV3B and C), or liver weight (Fig EV3D) in LXRα-deficient mice. Consistent with previous findings that LXRα-deficient mice are more susceptible to high-fat diet-induced hepatic steatosis and inflammation (Endo-Umeda *et al*, 2018; Endo-Umeda & Makishima, 2019), we observed significant hepatic steatosis after 4 weeks of HFCD (Fig 4A and B). Although SH42 treatment largely increased plasma desmosterol levels also in LXRα-deficient mice (+80%, Fig EV3E), it failed to improve hepatic steatosis (Fig 4A) as evidenced by an unchanged steatosis score (Fig 4B) and similar lipid-positive areas stained by Oil Red O in SH42 treated mice compared with control (Fig 4A and C). Liver lipidomic analysis further revealed that almost no lipid species was significantly changed upon SH42 treatment as shown by volcano plot analysis (Fig 4D). Accordingly, SH42 treatment showed no effects on hepatic concentrations

 © 2023 The Authors

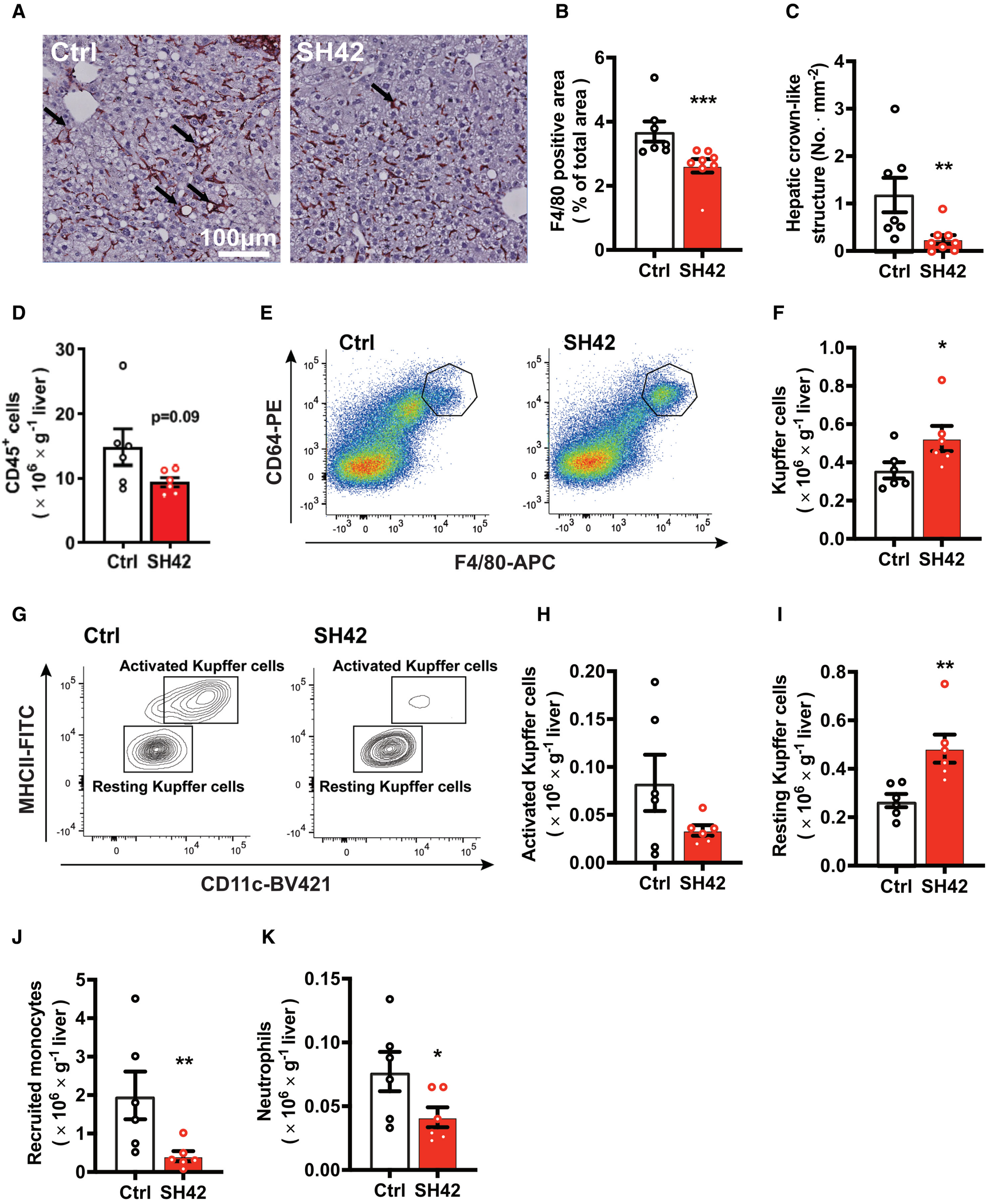
A
Ctrl
SH42
100μm
B
F4/80 positive area
(% of total area)
***
Ctrl SH42
C
Hepatic crown-like structure (No. · mm-2)
**
Ctrl SH42
D
CD45+ cells
(× 10^6 × g^-1 liver)
p=0.09
Ctrl SH42
E
Ctrl
SH42
CD64-PE
F4/80-APC
F
Kupffer cells
(× 10^6 × g^-1 liver)
*
Ctrl SH42
G
Ctrl
SH42
Activated Kupffer cells
Resting Kupffer cells
MHCII-FITC
CD11c-BV421
H
Activated Kupffer cells
(× 10^6 × g^-1 liver)
Ctrl SH42
I
Resting Kupffer cells
(× 10^6 × g^-1 liver)
**
Ctrl SH42
J
Recruited monocytes
(× 10^6 × g^-1 liver)
**
Ctrl SH42
K
Neutrophils
(× 10^6 × g^-1 liver)
*
Ctrl SH42

Figure 2.

◀ **Figure 2. Inhibition of DHCR24 by SH42 prevents Kupffer cell activation and reduces immune cell infiltration into the liver.**

E3LCETP mice fed a HFCD were treated with vehicle (Ctrl) or DHCR24 inhibitor SH42 (SH42) ($n = 8$ mice per group). After 8 weeks of treatment, mice were killed and livers were collected to stain with F4/80.

A, B Representative pictures of F4/80 staining are shown and (B) F4/80 positive area was quantified.

C, D Hepatic crown-like structures as indicated by the arrows in Fig 2A were counted ($n = 7$ and 8 mice, respectively; one value was identified as an outlier based on a Grubbs' test and removed from statistical analysis). In another experiment after 4 weeks of treatment ($n = 6$ mice per group), fresh livers were collected to isolate and count (D) CD45^{+} cells.

E–K (E and F) Total Kupffer cells (KCs), (G and H) activated KCs, (G and I) resting KCs, (J) recruited monocytes, and (K) neutrophils in the liver were measured via flow cytometry.

Data information: Values are mean ± SEM. Differences between two groups (SH42/Ctrl) were determined using a Mann–Whitney test. *$P < 0.05$, **$P < 0.01$, ***$P < 0.001$ vs. ctrl. Scale bar: 100 µm.
Source data are available online for this figure.

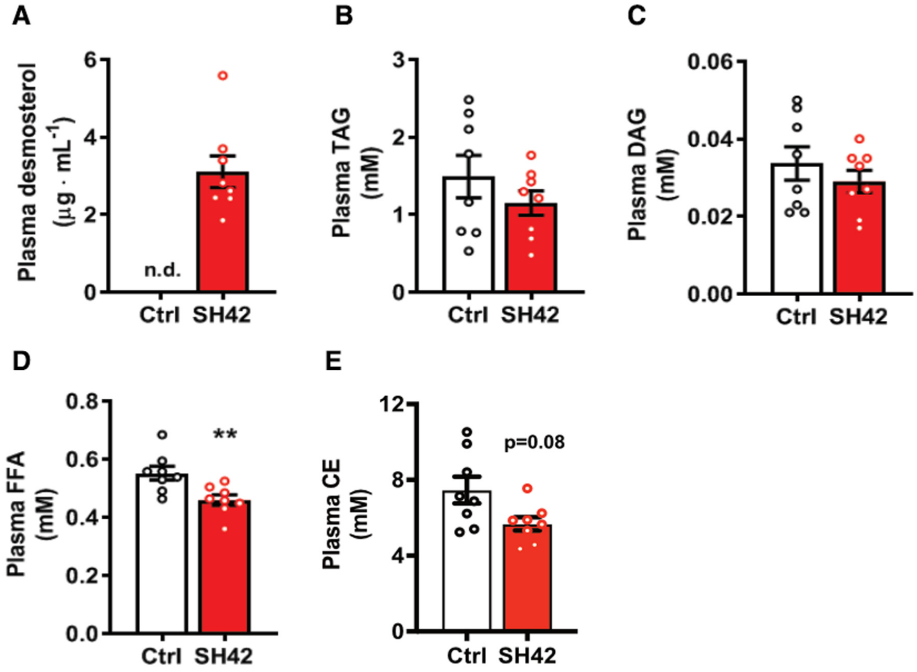

Figure 3. Inhibition of DHCR24 by SH42 does not increase circulating lipids.

E3LCETP mice fed a HFCD were treated with vehicle (Ctrl) or DHCR24 inhibitor SH42 (SH42) ($n = 8$ mice per group).

A After 8 weeks of treatment, blood was collected to measure desmosterol levels and for quantitative lipidomic analysis.

B–E Plasma (B) TAG, (C) DAG, (D) FFA and (E) CE lipid class concentrations were summarized.

Data information: Values are mean ± SEM. Differences between two groups (SH42/Ctrl) were determined using a Mann–Whitney test. **$P < 0.01$ vs. ctrl. CE, cholesteryl esters; DAG, diacylglycerols; FFA, free fatty acids; TAG, triacylglycerols.
Source data are available online for this figure.

of TAG (Fig 4E), DAG (Fig 4F), FFA (Fig 4G), or CE (Fig 4H) in the absence of LXRα.

The therapeutic effects of DHCR24 inhibition by SH42 on Kupffer cell activation and hepatic monocyte infiltration are strictly dependent on LXRα

Subsequently, we analyzed hepatic immune cells by flow cytometry after 4 weeks of treatment with SH42 in HFCD-fed LXRα-deficient mice. In contrast to *E3L.CETP* mice, SH42 did not affect hepatic leukocytes (Fig 5A) in LXRα-deficient mice. Strikingly, SH42 did not affect KC numbers (Fig 5B and C), and did not prevent KC activation as evidenced by unchanged high levels of NASH-associated MHCII^{+}CD11c^{+}-activated KCs (Fig 5D and E) and MHCII^{-}CD11c^{-} resting KCs (Fig 5D and F). SH42 did not reduce recruited monocytes in liver (Fig 5G) but reduced hepatic neutrophils in LXRα-deficient mice (−42%; Fig 5H). Congruent with these data, circulating monocytes and neutrophils were not affected by SH42 treatment (Fig EV4A–C). Taken together, these data show that inhibition of DHCR24 by SH42 does not prevent diet-induced hepatic steatosis and inflammation in LXRα-deficient mice.

 © 2023 The Authors

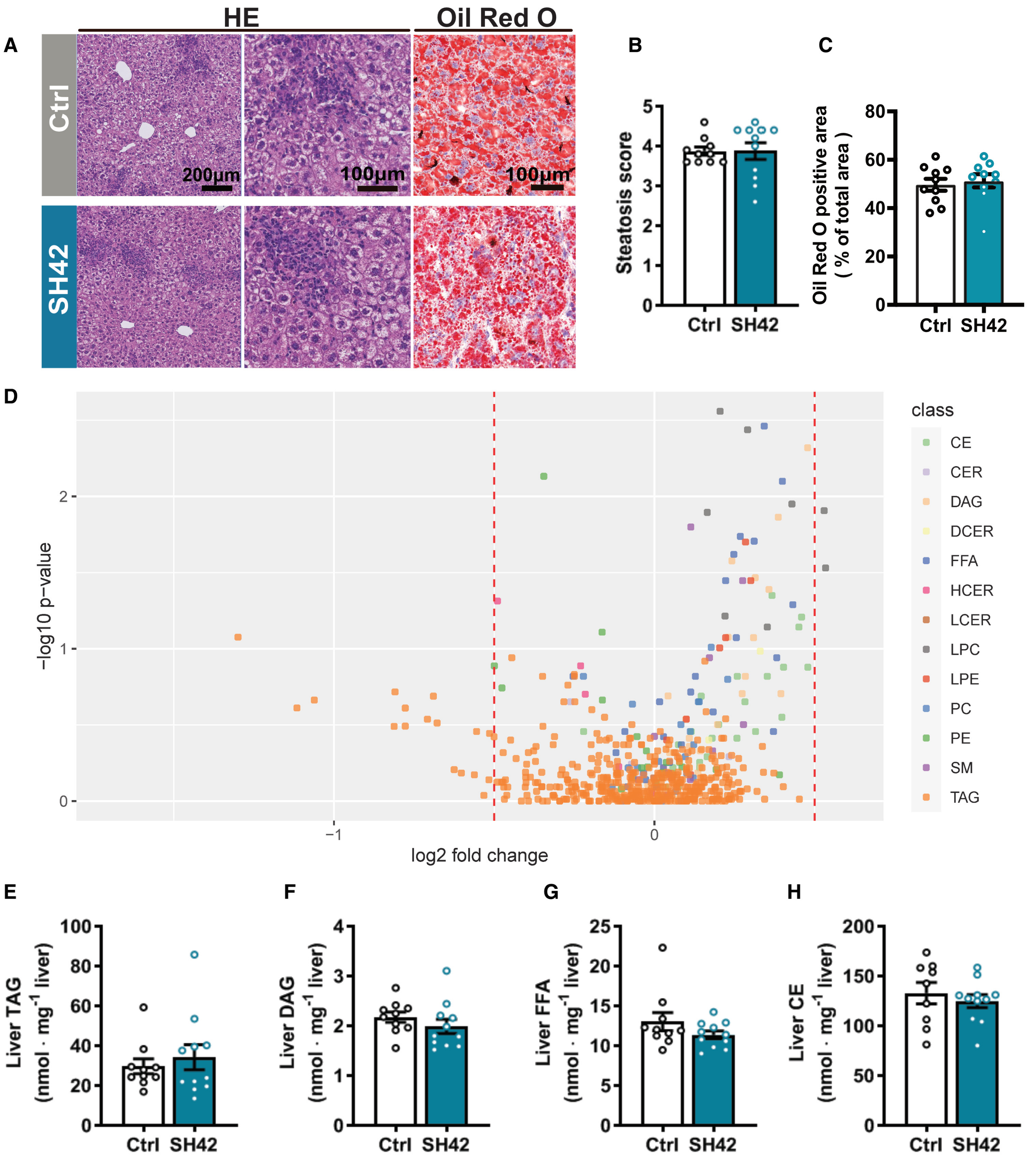
A
HE
Oil Red O
Ctrl
SH42
200μm
100μm
100μm
B
Steatosis score
Ctrl SH42
C
Oil Red O positive area (% of total area)
Ctrl SH42
D
−log10 p−value
log2 fold change
class
CE
CER
DAG
DCER
FFA
HCER
LCER
LPC
LPE
PC
PE
SM
TAG
E
Liver TAG (nmol · mg⁻¹ liver)
Ctrl SH42
F
Liver DAG (nmol · mg⁻¹ liver)
Ctrl SH42
G
Liver FFA (nmol · mg⁻¹ liver)
Ctrl SH42
H
Liver CE (nmol · mg⁻¹ liver)
Ctrl SH42

Figure 4.

Figure 4. The therapeutic effects of DHCR24 inhibition on hepatic steatosis are strictly dependent on LXRα.

LXRα-deficient mice fed HFCD were treated with vehicle (Ctrl) or DHCR24 inhibitor SH42 (SH42) (n = 10 and 11 mice, respectively).

A After 4 weeks of treatment, mice were killed and liver samples were collected to stain with HE and Oil Red O.

B, C Hepatic steatosis was scored using the HE-stained slides and (C) lipid-positive area was quantified using the Oil Red O stained slides.

D Volcano plot analysis of liver lipid species concentrations is shown.

E–H Hepatic (E) TAG, (F) DAG, (G) FFA and (H) CE lipid class concentrations were summarized. Benjamini–Hochberg correction was used for multiple hypothesis testing and Benjamini–Hochberg adjusted P-values are presented (P_adj). One outlier was identified and excluded in the hepatic CE analysis.

Data information: Values are mean ± SEM. Differences between two groups (SH42/Ctrl) were determined using a Mann–Whitney test if not indicated otherwise. Scale bar: 100 μm or 200 μm as indicated. CE, cholesteryl esters; CER, ceramides; DAG, diacylglycerols; DCER, dihydroceramides; FFA, free fatty acids; HCER, hexosylceramides; LPC, lysophosphatidylcholines; LPE, lysophosphatidylethanolamines; PC, phosphatidylcholines; PE, phosphatidylethanolamines; SM, sphingomyelins; TAG, triacylglycerols. Source data are available online for this figure.

Treatment with SH42 reduces hepatic crown-like structures, liver collagen content, and plasma alanine transaminase levels in an established NAFLD model

Translationally, we next investigated whether the inhibition of DHCR24 could rescue NASH progression. To this end, *E3L.CETP* mice were fed with a HFCD for 10 weeks first to establish NAFLD and then treated with vehicle or SH42 while still on HFCD for an additional 8 weeks (Fig 6A). SH42 treatment did not significantly influence either total body weight (Appendix Fig S7A) or composition (Fig EV5A–C). Although we did not observe any difference in liver weight (Fig EV5D) and hepatic steatosis (Fig 6B–D), SH42 treatment reduced hepatic crown-like structures (−89%; Fig 6E and F) without significant influence on the F4/80 positive area (Fig 6E and G), and ameliorated liver collagen content (−50%; Fig 6H and I). In addition, SH42 treatment reduced plasma levels of the liver injury marker plasma alanine transaminase (ALT; −42%; Fig 6J). These effects were accompanied by a robust increase in plasma desmosterol levels (> 400-fold; Fig EV5E).

Discussion

As key regulators of metabolic and inflammatory signaling (Bensinger & Tontonoz, 2008; Ito *et al*, 2015), LXRs have taken center stage as potential therapeutic targets for the treatment of cardiometabolic diseases. However, undesirable side effects of pharmacological LXR activation, including hyperlipidemia via induction of *Srebf1c* expression and neutropenia (Grefhorst *et al*, 2002; Kirchgessner *et al*, 2016), have prevented clinical application. Interestingly, desmosterol has been reported to be an endogenous ligand of LXR while inhibiting SREBP activity (Muse *et al*, 2018). Therefore, enhancing endogenous desmosterol levels by targeting DHCR24 is already considered as a promising strategy for activating LXR transcription programs to combat atherosclerotic cardiovascular disease (Rodriguez-Acebes *et al*, 2009; Spann *et al*, 2012; Muse *et al*, 2018). In this study, we exploited the synthetic DHCR24 inhibitor SH42, developed by our group (Muller *et al*, 2017), for the treatment of hepatic steatosis and inflammation, that is, the two hallmarks of NAFLD/NASH development. We show that inhibition of DHCR24 induces marked increases in desmosterol levels in both liver and circulation, which is accompanied by decreased hepatic steatosis and inflammation. Experiments performed in LXRα-deficient mice demonstrated that the therapeutic effects of SH42 are strictly dependent on LXRα activation, most likely due to increased desmosterol, as we have previously shown that SH42 does not have intrinsic LXR affinity (Korner *et al*, 2019).

We found that inhibition of DHCR24 by SH42 markedly increased desmosterol levels in both *E3L.CETP* mice and LXRα-deficient mice, while SH42 prevented circulating monocyte recruitment and increased MHCII$^-$/CD11c$^-$ resting KCs in *E3L.CETP* mice only (Fig 2G–J). This effect on monocytes/KCs is likely attributed to the direct effects of desmosterol-induced LXRα activation in monocytes/KCs, based on our previous study showing that desmosterol activates LXR in macrophages but not in hepatocytes (Muse *et al*, 2018; Snodgrass *et al*, 2020). In addition, we previously showed that inhibition of DHCR24 by SH42 increased desmosterol and induced the expression of LXR target genes, including *Abca1* and *Abcg1* in RAW 264.7 macrophages (Korner *et al*, 2019). LXRα is highly expressed in KCs and the region of enhancers unique to KCs are enriched in LXR-regulating sequence motifs (Lavin *et al*, 2014), indicating a role of LXRs in KC homeostasis. Accordingly, we here observed that about 95% of KCs in LXRα-deficient mice displayed markers indicative of activated KCs (i.e., MHCII$^+$/CD11c$^+$ KCs) versus less than 25% of KCs in wild-type mice upon feeding the same HFCD, which is in agreement with our previous study in LXRα-deficient mice showing a robust increase in KC activation and hepatic inflammation (Endo-Umeda *et al*, 2018). Taken together, our study suggests that pharmacological inhibition of DHCR24 is a feasible way to prevent KC activation and alleviate liver inflammation through desmosterol-induced LXRα activation.

Importantly, we observed that inhibition of DHCR24 by SH42 prevented high-fat diet-induced hepatic steatosis in *E3L.CETP* mice (Fig 1) but not in LXRα-deficient mice (Fig 4). We excluded the potential contribution of altered systemic glucose homeostasis in this process as evidenced by unchanged body composition, circulating glucose and insulin levels, as well as HOMA-IR scores in both groups (Fig EV1). We reasoned that the alleviated liver inflammation may largely contribute to the reduction of hepatic steatosis. The feed-forward loop between lipotoxic hepatocytes and proinflammatory immune cells, especially KCs, promotes NAFLD/NASH progression (Hirsova & Gores, 2015). Our study strongly suggests that this vicious circle can be abrogated by desmosterol-induced LXRα activation in liver-resident KCs. Consistently, both prevention of KC activation and depletion of KCs reduces liver inflammation (Bieghs *et al*, 2012) and alleviates hepatic steatosis (Huang *et al*, 2010; Lanthier *et al*, 2011). Therefore, the alleviated liver inflammation could be the direct effect of desmosterol in macrophages/KCs, which subsequently prevents hepatic steatosis. Additionally, the alleviation of liver inflammation has been shown to have multiple

 © 2023 The Authors

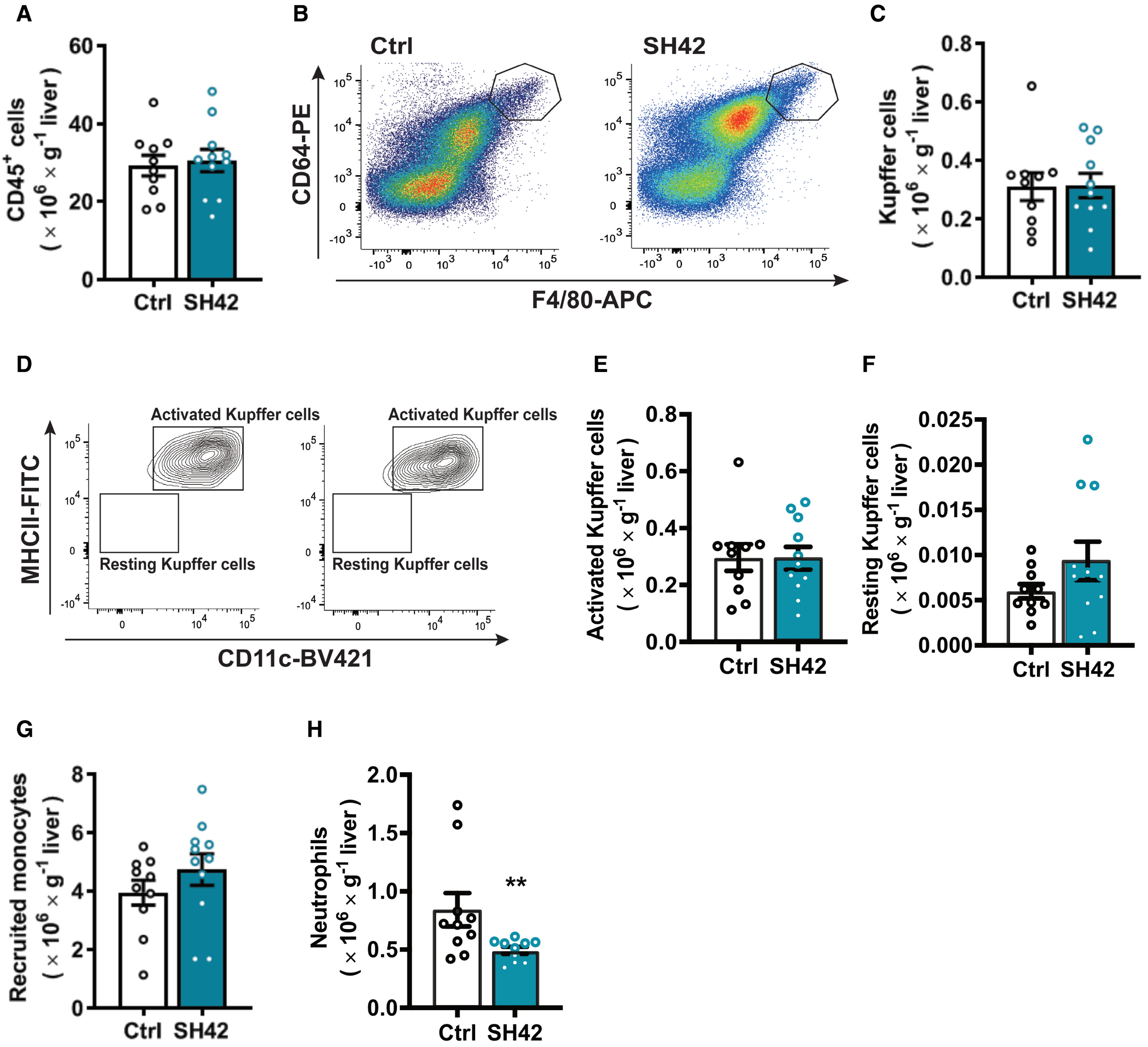

Figure 5. The therapeutic effects of DHCR24 inhibition on liver inflammation are strictly dependent on LXRα.

LXRα-deficient mice fed HFCD were treated with vehicle (Ctrl) or DHCR24 inhibitor SH42 (SH42) ($n = 10$ and 11 mice, respectively).

A After 4 weeks of treatment, mice were killed and fresh liver samples were collected to isolate and count $CD45^+$ cells.

B–H (B and C) Total Kupffer cells (KCs), (D and E) activated KCs, (D and F) resting KCs, (G) recruited monocytes, and (H) neutrophils in the liver were measured via flow cytometry.

Data information: Values are mean ± SEM. Differences between two groups (SH42/Ctrl) were determined using a Mann–Whitney test. $^{**}P < 0.01$ vs. ctrl.
Source data are available online for this figure.

effects, including reduced fatty acid uptake, VLDL secretion, and lipid oxidation (Liu *et al*, 2014), which could be the cause of a reduction in liver lipids by SH42 treatment, and needs to be further investigated.

Pharmaceutical development of LXR agonists has been challenged by the fact that these compounds usually induce *de novo* lipogenesis (Grefhorst *et al*, 2002; Kirchgessner *et al*, 2016), which is mainly attributable to hepatocyte-specific LXRα activation (Bradley *et al*, 2007; Zhang *et al*, 2012). Importantly, DHCR24 inhibition by SH42 decreases plasma FFA and CE levels, without increasing plasma total TAG and DAG levels (Fig 3), which implies that LXRα activation by desmosterol, in contrast to synthetic agonists, does not lead to hyperlipidemia. This can be easily understood from the fact that desmosterol activates LXRα in macrophages/KCs only, whereas

© 2023 The Authors

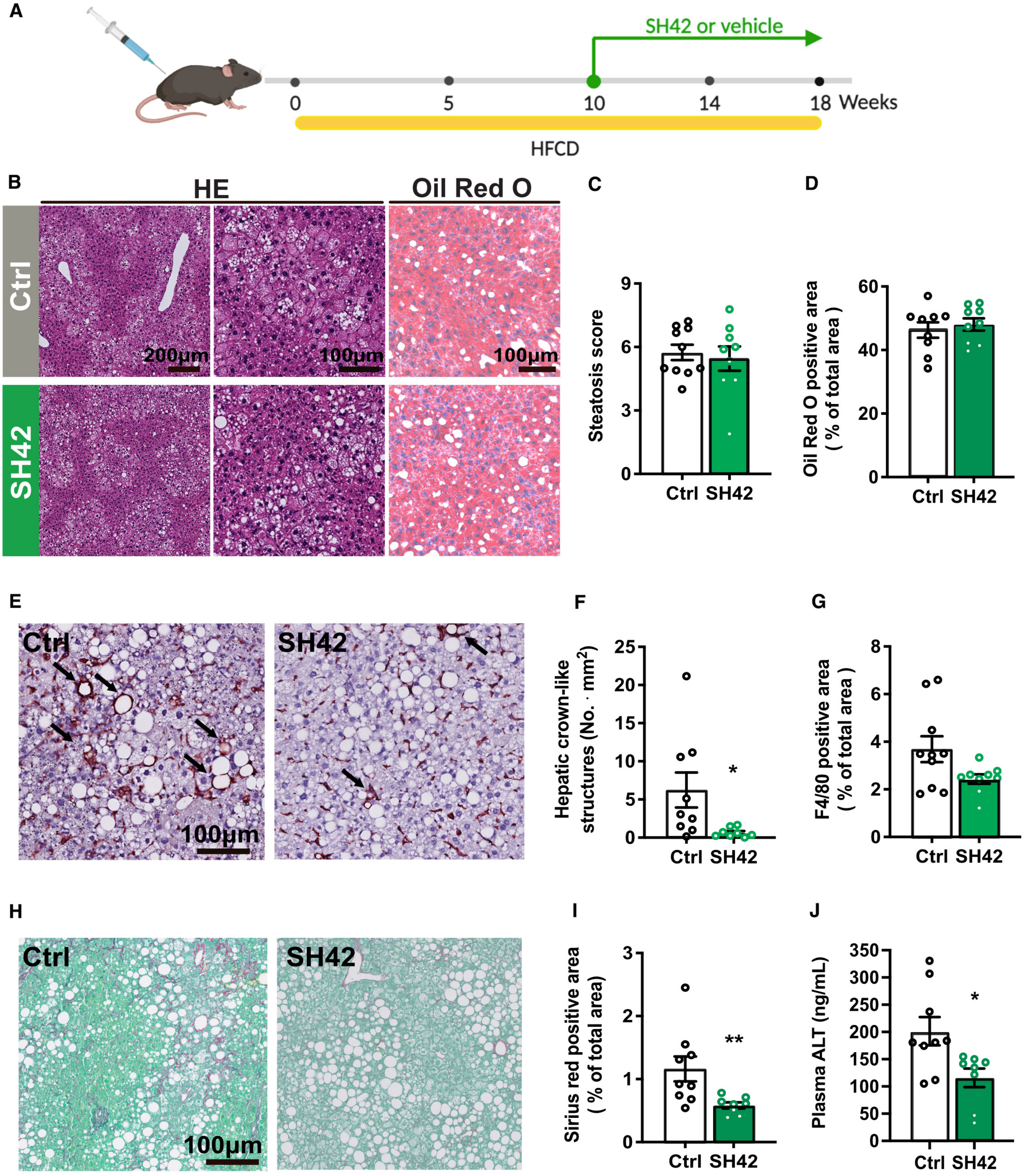

Figure 6.

 © 2023 The Authors

Figure 6. Treatment with SH42 reduces hepatic crown-like structures, liver collagen content, and plasma alanine transaminase levels in an established NAFLD mode.

A *E3L.CETP* mice were fed with a HFCD for 10 weeks first and then treated with vehicle (Ctrl) or DHCR24 inhibitor SH42 (SH42) (n = 10 and 9 mice, respectively) for additional 8 weeks. Mice were killed and livers were collected.

B Liver sections were stained with hematoxylin and eosin (HE) and Oil Red O.

C, D Hepatic steatosis was scored using the HE-stained slides and (D) lipid-positive area was quantified using the Oil Red O stained slides.

E–G Liver sections were stained with F4/80 (E) to quantify (F) hepatic crown-like structures as indicated by the arrows in Fig 6E and (G) F4/80 positive area.

H, I To quantify the collagen content, liver sections were stained with (H) Sirius red/Fast green and (I) quantified accordingly.

J Blood samples were collected after 18 weeks of treatment and plasma alanine transaminase (ATL) levels were measured.

Data information: Values are mean ± SEM. Differences between groups (SH42/Ctrl) were determined using a Mann–Whitney test. *P < 0.05, **P < 0.01, vs. ctrl. Scale bar: 100 or 200 μm as indicated.
Source data are available online for this figure.

LXR-induced hyperlipidemia is mainly due to increased *de novo* lipogenesis within hepatocytes (Muse *et al*, 2018). In fact, we have shown that desmosterol even suppresses SREBPs in macrophages via an Insig/SREBP cleavage-activating protein (SCAP)-dependent mechanism (Rodriguez-Acebes *et al*, 2009; Spann *et al*, 2012; Muse *et al*, 2018). Taken together, SH42-induced desmosterol does not evoke an undesirable increase in plasma and hepatic lipid levels that are typical for synthetic LXR agonists, likely by selective LXRα activation in macrophages/KC rather than hepatocytes.

A human study has demonstrated that desmosterol levels in circulation and liver are associated with NASH development (Simonen *et al*, 2013). However, whether desmosterol has a specific role in the pathophysiology of NAFLD/NASH is still unknown. To the best of our knowledge, our present study for the first time shows the potential application of pharmacological DHCR24 inhibition to enhance endogenous desmosterol levels for the treatment of NAFLD/NASH. Interestingly, a recent genetic study demonstrated that the rs588709 variant near the *DHCR24* locus is associated with lower circulating TAG-rich VLDL particles (Sliz *et al*, 2020), which potentially extends the role of DHCR24 to human lipid metabolism. Triparanol is the most widely used (nonspecific) DHCR24 inhibitor (Muller *et al*, 2019); however, it was withdrawn from clinical application due to severe adverse side effects, such as nausea and vomiting, cataracts, and skin disorders (Kirby, 1967). In contrast to Triparanol with an IC_{50} value of 14 μM, SH42 is a much more potent DHCR24 inhibitor, having an IC_{50} value of less than 10 nM in a cellular assay (Moebius *et al*, 1998; Muller *et al*, 2017) as judged by desmosterol accumulation and ^{13}C labelling of cholesterol. In our preclinical mouse model, we did not observe adverse effects of SH42 treatment on food intake, body weight, and body composition. Additionally, desmosterol-induced LXR activation via inhibition of DHCR24 did not cause a reduction of circulating neutrophils or neutropenia, another adverse effect of systemic LXR activation (Kirchgessner *et al*, 2016). Importantly, our study showed that DHCR24 inhibition by SH42 not only prevented but also affected NASH progression in mice with established hepatic steatosis, as evidenced by reducing the number of hepatic crown-like structures, collagen content, and plasma alanine transaminase levels. These advantages of targeting DHCR24 over synthetic LXR agonists will possibly lead to a promising application of DHCR24 inhibitors to combat NAFLD/NASH, in addition to other cardiometabolic and inflammatory diseases. Of note, our study is limited by our inability to provide direct evidence of SH42 binding to DHCR24, due to a lack of available pure enzyme and the fact that DHCR24 is membrane-bound making its isolation and co-crystallization very difficult. Therefore, we cannot exclude that SH42 exerts indirect inhibitory actions responsible for desmosterol accumulation or interaction with yet undiscovered targets contributing to the improvement of the NAFLD phenotype in our study.

In conclusion, we show that pharmacological inhibition of DHCR24 increases desmosterol to prevent diet-induced hepatic steatosis and inflammation, two main hallmarks of NAFLD/NASH development, without inducing hyperlipidemia. As such, our study paves the way for developing a new therapeutic strategy for the treatment of NAFLD/NASH.

Materials and Methods

Animals and treatments

Hemizygous *APOE*3-Leiden* (*E3L*) mice were crossbred with homozygous human cholesteryl ester transfer protein (CETP) transgenic mice to generate heterozygous *E3L.CETP* mice on a C57BL/6J background (Westerterp *et al*, 2006). Because of the phenotypical heterogeneity of *E3L.CETP* mice, nonresponder mice were identified by 4-h fasting plasma lipid levels, that is, total cholesterol levels < 2 mM and triglyceride levels < 2 mM, and excluded before high-fat high-cholesterol diet (HFCD) treatment (Tarasco *et al*, 2018). LXRα-deficient mice (also on C57BL/6J background), generated by Deltagen using gene-targeting methods as described (Plosch *et al*, 2006), were kindly provided by Tularik (San Francisco, CA, USA). Mice were group-housed in individually ventilated cages in standard conditions at room temperature (22°C) with 40 ± 5% relative humidity and a 12-h light/dark (7 am lights on; 7 pm lights off) cycle. Water and standard laboratory diet (801203, Special Diets Services, UK) were available *ad libitum*, unless indicated otherwise. This study was approved by the Animal Ethical Committee of Leiden University Medical Center, Leiden, The Netherlands (AVD1160020173305, PE.18.034.007) and the Animal Ethical Committee of University Groningen, Groningen, The Netherlands (PE.18.034.030, 173305-02-001). All animals received humane care according to the criteria outlined in the NIH "Guide for the Care and Use of Laboratory Animals." All animal procedures were performed conform the guidelines from Directive 2010/63/EU of the European Parliament on the protection of animals used for scientific purposes.

At the age of 10–12 weeks, male *E3L.CETP* mice and LXRα-deficient mice were fed a HFCD (Altromin, Germany) containing

© 2023 The Authors

60% (energy) fat and 1% (wt/wt) cholesterol, and were randomized into two groups treated with either the DHCR24 inhibitor SH42 (0.5 mg•mouse^{-1}; Muller *et al*, 2017; Korner *et al*, 2019) or vehicle (saline containing 3.3% ethanol and 3.3% Cremophor EL) 3 times per week by intraperitoneal injection. *E3L.CETP* mice were treated for 4 weeks (n = 6 mice per group) and 8 weeks (n = 8 mice per group) to evaluate effects on hepatic immune cells via flow cytometry analysis and hepatic steatosis via quantitative lipidomic analysis, respectively. LXRα-deficient mice were treated with either SH42 (n = 11 mice) or vehicle (n = 10 mice) for 4 weeks to evaluate effects on hepatic steatosis and immune cells. In a rescue experiment, male *E3L.CETP* mice were fed with HFCD for 10 weeks first and cotreated with SH42 (0.5 mg•mouse^{-1}) or vehicle (saline containing 3.3% ethanol and 3.3% Cremophor EL) 3 times per week by intraperitoneal injection for additional 8 weeks.

Body weight was measured weekly. Body composition (i.e., fat body and lean body mass; EchoMRI-100; EchoMRI, Houston, TX, USA) was evaluated before and after intervention. Food intake was determined during the treatment period.

Plasma glucose, insulin, and ALT assay

Four-hour-fasted (9 am till 1 pm) blood samples were collected via tail vein of *E3L.CETP* mice bleeding into heparin-coated capillaries after 8 weeks of treatment with vehicle or SH42. Blood samples were then centrifuged at 15,000 *g* for 5 min at 4°C to collect plasma. 2.5 µl plasma was used to measure glucose (INstruchemie, The Netherlands) and 10 µl to measure insulin (Mercodia AB, Sweden) according to the manufacturers' protocols. The homeostatic Model Assessment for Insulin Resistance (HOMA-IR) scores were calculated using the equation: HOMA-IR = fasting insulin (mU l^{-1}) × fasting glucose (mmol l^{-1})/22.5 (Matthews *et al*, 1985).

To measure ALT levels, 4 h-fasted (9 am till 1 pm) blood samples were collected via tail vein of *E3L.CETP* mice after 16 weeks of HFCD treatment. Blood samples were then centrifuged at 15,000 *g* for 5 min at 4°C to collect plasma. Two microliter plasma was diluted 200 times and used for plasma ALT assay (ab282882, Abcam, UK) according to the manufacturer's protocol.

Liver histology and hepatic steatosis scoring

After 8 or 18 weeks of treatment in *E3L.CETP* mice or 4 weeks in LXRα-deficient mice, mice were killed and transcardially perfused with ice-cold PBS, and a small piece of the right liver lobe was collected and fixed in phosphate-buffered 4% formaldehyde. Fixed tissues were embedded in paraffin and cut into sections of 5 µm thickness for staining with hematoxylin and eosin (HE) and/or the macrophage marker F4/80 (1 µg ml^{-1}, MCA497, Serotec, Oxford, UK), which was detected using ImmPRESS HRP goat anti-rat IgG detection kit (MP-744-15, Vector Laboratories, CA, USA). Hepatic steatosis was evaluated from HE-stained slides by two blinded experts, respectively, with the criteria proposed by Liang *et al* (2014). Hepatocellular vesicular steatosis, that is, microvesicular steatosis and macrovesicular steatosis, and hepatocellular hypertrophy were scored (grade 0–3) based on the percentage of the total area affected, and the grades were summed into one standard as "steatosis score" (0–9). Liver Oil Red O staining was performed on frozen liver tissue sections to visualize and quantify lipid droplets using ImageJ software (version 1.50i). F4/80 positive area using F4/80 stained slides were quantified using ImageJ software (version 1.50i). Hepatic crown-like structures formed by macrophages aggregating around dead hepatocytes were counted from F4/80 stained slides, which were blinded before analysis and expressed as number per area (Itoh *et al*, 2013). To quantify the collagen content, liver sections were stained with Sirius red/Fast green and quantified using ImageJ software (version 1.50i). Values in figures for each staining present means of nine randomly selected image fields (0.468 mm^2 per field) per mouse. The antibody used is listed in Appendix Table S1.

GC–MS analysis of desmosterol

After 8 weeks of treatment in *E3L.CETP* mice or 4 weeks in LXRα-deficient mice, desmosterol levels were measured using GC–MS after alkaline hydrolysis, as described previously for plasma (Muller *et al*, 2017) and liver (Korner *et al*, 2019). In brief, a Bruker Scion GC–MS system (used for *E3L.CETP* mice) or an Agilent 7890B coupled to a 5977B MS (used for LXR KO mice) were utilized and operated in selected ion monitoring mode (SIM). An Agilent VF-5 ms column, 25 m × 0.25 mm × 0.25 µm was used. Helium 99.9990% was used as carrier gas at a constant flow rate of 1.4 ml min^{-1}. Injector and transfer line were operated at 280°C. The oven program started at 90°C held for 0.5 min, then ramped to 270 at 30°C min^{-1}, and ramped to 310 at 10°C min^{-1}. The following masses were used to quantify desmosterol levels against an external calibration line (0–10 ppm) m/z 445 + 355 for cholestanol (IS), m/z 357 and 271 for cholestan (IS) and m/z 343 + 253 or 441.2 for desmosterol. Desmosterol was identified by matching characteristic ions and retention times with an authentic standard (Sigma Aldrich).

Quantitative lipidomic analysis

Quantitative lipidomic analysis was carried out in liver and plasma samples in *E3L.CETP* mice and in LXRα-deficient mice as described elsewhere (Korner *et al*, 2019). Briefly, a small piece of the right liver lobe was homogenized with 600 µl LCMS grade water in a bullet blender for 30 s and an aliquot corresponding to 5 mg liver tissue was used. For plasma samples, 25 µl was used. 25 µl internal standard mix (100 µl for liver samples; Lipidyzer™ internal standard kit, containing > 50 labeled internal standards for 13 lipid classes, Sciex cat# 504156), 500 µl methyl tert-butyl ether (MTBE), and 160 µl methanol were subsequently added and the mixture was shaken for 30 min at room temperature. After adding 200 µl water, samples were centrifuged at 16,000 *g* for 3 min and the upper organic layer was collected in a glass vial. The remaining sample was extracted again by adding 300 µl MTBE, 100 µl methanol, and 100 µl water for 30 min. The organic extracts were then pooled and dried under a gentle stream of nitrogen. The dry extract was subsequently dissolved in 250 µl Lipidyzer running buffer and analyzed according to the manufacturer's protocol. For data analysis, 13 lipid classes, including cholesteryl esters (CE), ceramides (CER), diacylglycerols (DAG), dihydroceramides (DCER), free fatty acids (FFA), hexosylceramides (HCER), lysophosphatidylcholines (LPC), lysophosphatidylethanolamines (LPE), phosphatidylcholines (PC), phosphatidylethanolamines (PE), sphingomyelins (SM) and triacylglycerols (TAG) were compared.

© 2023 The Authors

Isolation of liver immune cells and peripheral blood mononuclear cells

After 4 weeks of SH42 treatment, blood and liver were collected and processed for flow cytometry as described previously (Hussaarts *et al*, 2015). In short, blood was collected from the retro-orbital sinus of anesthetized mice into heparin capillaries, after which mice were killed and transcardially perfused with PBS for 5 min to remove circulating cells. A piece of right liver lobe was cut off for other liver assays and the remainder of liver tissues were minced with a scalpel to form a paste, digested with collagenase type IV (Sigma Aldrich, USA), and hepatocytes were removed by low gravity centrifugation at 50 g for 3 min. $CD45^+$ leukocytes were isolated using CD45 microbeads (35 µl beads per liver; Miltenyi Biotec, USA) and an LS column, according to the manufacturer's instructions. In addition, peripheral blood mononuclear cells were isolated. To this end, 450 µl blood was mixed with red blood cell lysis/fix buffer (Becton Dickinson, USA) (3:20; v/v) for 15 min at room temperature. Subsequently, white blood cells were pelleted by centrifugation at 600 *g* for 5 min. The supernatant was discarded and cells were resuspended and washed with PBS. The cells isolated from the liver and blood were counted and characterized by flow cytometry.

Flow cytometry analysis

All cell suspensions were stained with the fixable viability dye Zombie-UV (BioLegend, USA) and fixed with 1.9% paraformaldehyde. Within 24 h of fixation, the cells were preincubated with an Fcγ blocking antibody (Thermo Fisher Scientific) for 15 min. The cells were then stained with fluorescently labeled antibodies listed in the Appendix Table S2 for 30 min at 4°C in the dark. Fluorescently labeled cells were measured on an LSRII (Becton Dickinson) and gates were set according to Fluorescence Minus One (FMO) controls using FlowJo™ Software (Becton Dickinson). Representative gating schemes are shown in Appendix Figs S3 and S4.

Statistical analysis

Group size was determined by power analysis of data from our previous studies to achieve a statistical power of 80% and *P*-value of 0.05. Mice were randomized into different groups before the treatments. Investigators were blinded for hepatic steatosis scoring and were not blinded for other assays. Outliers were identified using the Grubbs' test (https://www.graphpad.com/quickcalcs/Grubbs1.cfm) and removed from statistical analysis, which is clearly stated in figure legends if applicable. Because some datasets did not pass a statistical test for Gaussian distribution, differences between two groups were compared using a nonparametric Mann–Whitney test performed in GraphPad Prism 8.1 (GraphPad Software). For quantification of different lipid classes, Benjamini–Hochberg correction was used for multiple hypothesis testing (Figs 1G–J and 4E–H). $P < 0.05$ was considered significant (*$P < 0.05$, **$P < 0.01$, ***$P < 0.001$).

Data availability

The authors declare that all data supporting the findings of this study are available within the paper and its Supplementary Information Appendix. This study includes no data deposited in external repositories.

The paper explained

Problem

Although more than 30% of the general population have nonalcoholic fatty liver disease (NAFLD), no medication is available yet for the treatment of NAFLD. As the liver X receptor (LXR) is a key regulator of metabolic and inflammatory signaling, LXR agonism has a theoretical potential for treating NAFLD, but synthetic LXR agonists induce hyperlipidemia in preclinical models and humans.

Results

In this study, we show the potential application of pharmacological Δ24-dehydrocholesterol reductase (DHCR24) inhibition by SH42 to prevent diet-induced hepatic steatosis and liver inflammation in a well-established translational model with human-like NAFLD characteristics. Inhibition of DHCR24 increased the endogenous LXR agonist desmosterol to exert metabolic and immune benefits in a strictly LXRα-dependent manner without causing hyperlipidemia.

Impact

Pharmacological DHCR24 inhibition by SH42 increases desmosterol to prevent diet-induced hepatic steatosis and inflammation, two main hallmarks of NAFLD/NASH development, without inducing hyperlipidemia. Pharmacological DHCR24 inhibition may represent a novel therapeutic strategy to activate LXR for the treatment of NAFLD and potentially other cardiometabolic diseases.

Expanded View for this article is available online.

Acknowledgements

This work was supported by the Netherlands Organization for Health Research and Development-ZonMW (Early Career Scientist Hotel grant 435004007 to YW); the National Natural Science Foundation of China (grant 82170876 to YW); the Netherlands Cardiovascular Research Initiative: an initiative with the support of the Dutch Heart Foundation (CVON-GENIUS-2 to PCNR, CVON-IN CONTROL-2 to FK); the Netherlands Heart Foundation (2009T038 to PCNR); and the Department of Science and Technology Foundation of Shaanxi Province (2021SF-021 to YW). MG was partially supported by the Netherlands X-omics initiative, which is partially funded by NWO, project 184.034.019. YW is supported by the China "Thousand Talents Plan" (Young Talents), Shaanxi province "Thousand Talents Plan" (Young Talents), and Foundation of Xi'an Jiaotong University (Plan A).

Author contributions

Enchen Zhou: Conceptualization; data curation; formal analysis; investigation; visualization; methodology; writing – original draft; project administration; writing – review and editing. **Xiaoke Ge:** Formal analysis; investigation; methodology; writing – review and editing. **Hiroyuki Nakashima:** Conceptualization; data curation; formal analysis; investigation; visualization; methodology; writing – review and editing. **Rumei Li:** Formal analysis; investigation; methodology; writing – review and editing. **Hendrik J P van der Zande:** Formal analysis; investigation; methodology; writing – review and editing. **Cong Liu:** Investigation; writing – review and editing. **Zhuang Li:** Investigation; writing – review and editing. **Christoph Müller:** Resources; writing – review and editing. **Franz Bracher:** Resources; writing –

review and editing. **Yassene Mohammed:** Data curation; formal analysis; visualization; writing – review and editing. **Jan Freark de Boer:** Investigation; project administration; writing – review and editing. **Folkert Kuipers:** Resources; project administration; writing – review and editing. **Bruno Guigas:** Resources; project administration; writing – review and editing. **Christopher K Glass:** Resources; supervision; project administration; writing – review and editing. **Patrick C N Rensen:** Conceptualization; resources; data curation; supervision; funding acquisition; project administration; writing – review and editing. **Martin Giera:** Conceptualization; resources; data curation; supervision; funding acquisition; project administration; writing – review and editing. **Yanan Wang:** Conceptualization; resources; data curation; supervision; funding acquisition; project administration; writing – review and editing.

Disclosure and competing interests statement

The authors declare that they have no conflict of interest.

References

Aparicio-Vergara M, Hommelberg PP, Schreurs M, Gruben N, Stienstra R, Shiri-Sverdlov R, Kloosterhuis NJ, de Bruin A, van de Sluis B, Koonen DP *et al* (2013) Tumor necrosis factor receptor 1 gain-of-function mutation aggravates nonalcoholic fatty liver disease but does not cause insulin resistance in a murine model. *Hepatology* 57: 566 – 576

Baffy G (2009) Kupffer cells in non-alcoholic fatty liver disease: the emerging view. *J Hepatol* 51: 212 – 223

Bensinger SJ, Tontonoz P (2008) Integration of metabolism and inflammation by lipid-activated nuclear receptors. *Nature* 454: 470 – 477

Berlanga A, Guiu-Jurado E, Porras JA, Auguet T (2014) Molecular pathways in non-alcoholic fatty liver disease. *Clin Exp Gastroenterol* 7: 221 – 239

Bieghs V, van Gorp PJ, Walenbergh SM, Gijbels MJ, Verheyen F, Buurman WA, Briles DE, Hofker MH, Binder CJ, Shiri-Sverdlov R (2012) Specific immunization strategies against oxidized low-density lipoprotein: a novel way to reduce nonalcoholic steatohepatitis in mice. *Hepatology* 56: 894 – 903

Bieghs V, Walenbergh SM, Hendrikx T, van Gorp PJ, Verheyen F, Olde Damink SW, Masclee AA, Koek GH, Hofker MH, Binder CJ *et al* (2013) Trapping of oxidized LDL in lysosomes of Kupffer cells is a trigger for hepatic inflammation. *Liver Int* 33: 1056 – 1061

Bischoff ED, Daige CL, Petrowski M, Dedman H, Pattison J, Juliano J, Li AC, Schulman IG (2010) Non-redundant roles for LXRalpha and LXRbeta in atherosclerosis susceptibility in low density lipoprotein receptor knockout mice. *J Lipid Res* 51: 900 – 906

Bradley MN, Hong C, Chen M, Joseph SB, Wilpitz DC, Wang X, Lusis AJ, Collins A, Hseuh WA, Collins JL *et al* (2007) Ligand activation of LXR beta reverses atherosclerosis and cellular cholesterol overload in mice lacking LXR alpha and apoE. *J Clin Invest* 117: 2337 – 2346

Chalasani N, Younossi Z, Lavine JE, Charlton M, Cusi K, Rinella M, Harrison SA, Brunt EM, Sanyal AJ (2018) The diagnosis and management of nonalcoholic fatty liver disease: practice guidance from the American Association for the Study of Liver Diseases. *Hepatology* 67: 328 – 357

Endo-Umeda K, Makishima M (2019) Liver X receptors regulate cholesterol metabolism and immunity in hepatic nonparenchymal cells. *Int J Mol Sci* 20: 5045

Endo-Umeda K, Nakashima H, Umeda N, Seki S, Makishima M (2018) Dysregulation of Kupffer cells/macrophages and natural killer T cells in Steatohepatitis in LXRalpha knockout male mice. *Endocrinology* 159: 1419 – 1432

Grefhorst A, Elzinga BM, Voshol PJ, Plosch T, Kok T, Bloks VW, van der Sluijs FH, Havekes LM, Romijn JA, Verkade HJ *et al* (2002) Stimulation of lipogenesis by pharmacological activation of the liver X receptor leads to production of large, triglyceride-rich very low density lipoprotein particles. *J Biol Chem* 277: 34182 – 34190

Hirsova P, Gores GJ (2015) Death receptor-mediated cell death and Proinflammatory signaling in nonalcoholic Steatohepatitis. *Cell Mol Gastroenterol Hepatol* 1: 17 – 27

Huang W, Metlakunta A, Dedousis N, Zhang P, Sipula I, Dube JJ, Scott DK, O'Doherty RM (2010) Depletion of liver Kupffer cells prevents the development of diet-induced hepatic steatosis and insulin resistance. *Diabetes* 59: 347 – 357

Hussaarts L, Garcia-Tardon N, van Beek L, Heemskerk MM, Haeberlein S, van der Zon GC, Ozir-Fazalalikhan A, Berbee JF, Willems van Dijk K, van Harmelen V *et al* (2015) Chronic helminth infection and helminth-derived egg antigens promote adipose tissue M2 macrophages and improve insulin sensitivity in obese mice. *FASEB J* 29: 3027 – 3039

Ishibashi M, Filomenko R, Rebe C, Chevriaux A, Varin A, Derangere V, Bessede G, Gambert P, Lagrost L, Masson D (2013) Knock-down of the oxysterol receptor LXRalpha impairs cholesterol efflux in human primary macrophages: lack of compensation by LXRbeta activation. *Biochem Pharmacol* 86: 122 – 129

Ito A, Hong C, Rong X, Zhu X, Tarling EJ, Hedde PN, Gratton E, Parks J, Tontonoz P (2015) LXRs link metabolism to inflammation through Abca1-dependent regulation of membrane composition and TLR signaling. *Elife* 4: e08009

Itoh M, Kato H, Suganami T, Konuma K, Marumoto Y, Terai S, Sakugawa H, Kanai S, Hamaguchi M, Fukaishi T *et al* (2013) Hepatic crown-like structure: a unique histological feature in non-alcoholic steatohepatitis in mice and humans. *PLoS ONE* 8: e82163

Joseph SB, Bradley MN, Castrillo A, Bruhn KW, Mak PA, Pei L, Hogenesch J, O'Connell RM, Cheng G, Saez E *et al* (2004) LXR-dependent gene expression is important for macrophage survival and the innate immune response. *Cell* 119: 299 – 309

Kirby TJ (1967) Cataracts produced by triparanol (MER-29). *Trans Am Ophthalmol Soc* 65: 494 – 543

Kirchgessner TG, Sleph P, Ostrowski J, Lupisella J, Ryan CS, Liu X, Fernando G, Grimm D, Shipkova P, Zhang R *et al* (2016) Beneficial and adverse effects of an LXR agonist on human lipid and lipoprotein metabolism and circulating neutrophils. *Cell Metab* 24: 223 – 233

Korner A, Zhou E, Muller C, Mohammed Y, Herceg S, Bracher F, Rensen PCN, Wang Y, Mirakaj V, Giera M (2019) Inhibition of Delta24-dehydrocholesterol reductase activates pro-resolving lipid mediator biosynthesis and inflammation resolution. *Proc Natl Acad Sci USA* 116: 20623 – 20634

Lanthier N, Molendi-Coste O, Cani PD, van Rooijen N, Horsmans Y, Leclercq IA (2011) Kupffer cell depletion prevents but has no therapeutic effect on metabolic and inflammatory changes induced by a high-fat diet. *FASEB J* 25: 4301 – 4311

Lavin Y, Winter D, Blecher-Gonen R, David E, Keren-Shaul H, Merad M, Jung S, Amit I (2014) Tissue-resident macrophage enhancer landscapes are shaped by the local microenvironment. *Cell* 159: 1312 – 1326

Li P, Spann NJ, Kaikkonen MU, Lu M, Oh DY, Fox JN, Bandyopadhyay G, Talukdar S, Xu J, Lagakos WS *et al* (2013) NCoR repression of LXRs restricts macrophage biosynthesis of insulin-sensitizing omega 3 fatty acids. *Cell* 155: 200 – 214

Liang W, Menke AL, Driessen A, Koek GH, Lindeman JH, Stoop R, Havekes LM, Kleemann R, van den Hoek AM (2014) Establishment of a general NAFLD

 © 2023 The Authors

scoring system for rodent models and comparison to human liver pathology. *PLoS ONE* 9: e115922

Liu L, Mei M, Yang S, Li Q (2014) Roles of chronic low-grade inflammation in the development of ectopic fat deposition. *Mediators Inflamm* 2014: 418185

Matthews DR, Hosker JP, Rudenski AS, Naylor BA, Treacher DF, Turner RC (1985) Homeostasis model assessment: insulin resistance and beta-cell function from fasting plasma glucose and insulin concentrations in man. *Diabetologia* 28: 412 – 419

Moebius FF, Fitzky BU, Lee JN, Paik YK, Glossmann H (1998) Molecular cloning and expression of the human delta7-sterol reductase. *Proc Natl Acad Sci USA* 95: 1899 – 1902

Muller C, Hemmers S, Bartl N, Plodek A, Korner A, Mirakaj V, Giera M, Bracher F (2017) New chemotype of selective and potent inhibitors of human delta 24-dehydrocholesterol reductase. *Eur J Med Chem* 140: 305 – 320

Muller C, Junker J, Bracher F, Giera M (2019) A gas chromatography-mass spectrometry-based whole-cell screening assay for target identification in distal cholesterol biosynthesis. *Nat Protoc* 14: 2546 – 2570

Muse ED, Yu S, Edillor CR, Tao J, Spann NJ, Troutman TD, Seidman JS, Henke A, Roland JT, Ozeki KA *et al* (2018) Cell-specific discrimination of desmosterol and desmosterol mimetics confers selective regulation of LXR and SREBP in macrophages. *Proc Natl Acad Sci USA* 115: E4680 – E4689

Nes WD (2011) Biosynthesis of cholesterol and other sterols. *Chem Rev* 111: 6423 – 6451

Peet DJ, Turley SD, Ma W, Janowski BA, Lobaccaro JM, Hammer RE, Mangelsdorf DJ (1998) Cholesterol and bile acid metabolism are impaired in mice lacking the nuclear oxysterol receptor LXR alpha. *Cell* 93: 693 – 704

Plosch T, van der Veen JN, Havinga R, Huijkman NC, Bloks VW, Kuipers F (2006) Abcg5/Abcg8-independent pathways contribute to hepatobiliary cholesterol secretion in mice. *Am J Physiol Gastrointest Liver Physiol* 291: G414 – G423

Prufer K, Boudreaux J (2007) Nuclear localization of liver X receptor alpha and beta is differentially regulated. *J Cell Biochem* 100: 69 – 85

Remmerie A, Martens L, Thone T, Castoldi A, Seurinck R, Pavie B, Roels J, Vanneste B, De Prijck S, Vanhockerhout M *et al* (2020) Osteopontin expression identifies a subset of recruited macrophages distinct from Kupffer cells in the fatty liver. *Immunity* 53: 641 – 657

Riazi K, Azhari H, Charette JH, Underwood FE, King JA, Afshar EE, Swain MG, Congly SE, Kaplan GG, Shaheen AA (2022) The prevalence and incidence of NAFLD worldwide: a systematic review and meta-analysis. *Lancet Gastroenterol Hepatol* 7: 851 – 861

Rodriguez-Acebes S, de la Cueva P, Fernandez-Hernando C, Ferruelo AJ, Lasuncion MA, Rawson RB, Martinez-Botas J, Gomez-Coronado D (2009) Desmosterol can replace cholesterol in sustaining cell proliferation and regulating the SREBP pathway in a sterol-Delta24-reductase-deficient cell line. *Biochem J* 420: 305 – 315

Schultz JR, Tu H, Luk A, Repa JJ, Medina JC, Li L, Schwendner S, Wang S, Thoolen M, Mangelsdorf DJ *et al* (2000) Role of LXRs in control of lipogenesis. *Genes Dev* 14: 2831 – 2838

Schuster S, Cabrera D, Arrese M, Feldstein AE (2018) Triggering and resolution of inflammation in NASH. *Nat Rev Gastroenterol Hepatol* 15: 349 – 364

Seidman JS, Troutman TD, Sakai M, Gola A, Spann NJ, Bennett H, Bruni CM, Ouyang Z, Li RZ, Sun X *et al* (2020) Niche-specific reprogramming of epigenetic landscapes drives myeloid cell diversity in nonalcoholic Steatohepatitis. *Immunity* 52: 1057 – 1074

Simonen M, Mannisto V, Leppanen J, Kaminska D, Karja V, Venesmaa S, Kakela P, Kuusisto J, Gylling H, Laakso M *et al* (2013) Desmosterol in human nonalcoholic steatohepatitis. *Hepatology* 58: 976 – 982

Sliz E, Shin J, Syme C, Patel Y, Parker N, Richer L, Gaudet D, Bennett S, Paus T, Pausova Z (2020) A variant near DHCR24 associates with microstructural properties of white matter and peripheral lipid metabolism in adolescents. *Mol Psychiatry* 26: 3795 – 3805

Snodgrass RG, Benatzy Y, Schmid T, Namgaladze D, Mainka M, Schebb NH, Lutjohann D, Brune B (2020) Efferocytosis potentiates the expression of arachidonate 15-lipoxygenase (ALOX15) in alternatively activated human macrophages through LXR activation. *Cell Death Differ* 28: 1301 – 1316

Spann NJ, Garmire LX, McDonald JG, Myers DS, Milne SB, Shibata N, Reichart D, Fox JN, Shaked I, Heudobler D *et al* (2012) Regulated accumulation of desmosterol integrates macrophage lipid metabolism and inflammatory responses. *Cell* 151: 138 – 152

Tarasco E, Pellegrini G, Whiting L, Lutz TA (2018) Phenotypical heterogeneity in responder and nonresponder male ApoE*3Leiden.CETP mice. *Am J Physiol Gastrointest Liver Physiol* 315: G602 – G617

Tosello-Trampont AC, Landes SG, Nguyen V, Novobrantseva TI, Hahn YS (2012) Kuppfer cells trigger nonalcoholic steatohepatitis development in diet-induced mouse model through tumor necrosis factor-alpha production. *J Biol Chem* 287: 40161 – 40172

Tran S, Baba I, Poupel L, Dussaud S, Moreau M, Gelineau A, Marcelin G, Magreau-Davy E, Ouhachi M, Lesnik P *et al* (2020) Impaired Kupffer cell self-renewal alters the liver response to lipid overload during non-alcoholic Steatohepatitis. *Immunity* 53: 627 – 640

Venkateswaran A, Laffitte BA, Joseph SB, Mak PA, Wilpitz DC, Edwards PA, Tontonoz P (2000) Control of cellular cholesterol efflux by the nuclear oxysterol receptor LXR alpha. *Proc Natl Acad Sci USA* 97: 12097 – 12102

Wandrer F, Liebig S, Marhenke S, Vogel A, John K, Manns MP, Teufel A, Itzel T, Longerich T, Maier O *et al* (2020) TNF-Receptor-1 inhibition reduces liver steatosis, hepatocellular injury and fibrosis in NAFLD mice. *Cell Death Dis* 11: 212

Wang YY, Dahle MK, Agren J, Myhre AE, Reinholt FP, Foster SJ, Collins JL, Thiemermann C, Aasen AO, Wang JE (2006) Activation of the liver X receptor protects against hepatic injury in endotoxemia by suppressing Kupffer cell activation. *Shock* 25: 141 – 146

Westerterp M, van der Hoogt CC, de Haan W, Offerman EH, Dallinga-Thie GM, Jukema JW, Havekes LM, Rensen PC (2006) Cholesteryl ester transfer protein decreases high-density lipoprotein and severely aggravates atherosclerosis in APOE*3-Leiden mice. *Arterioscler Thromb Vasc Biol* 26: 2552 – 2559

Zhang Y, Breevoort SR, Angdisen J, Fu M, Schmidt DR, Holmstrom SR, Kliewer SA, Mangelsdorf DJ, Schulman IG (2012) Liver LXRalpha expression is crucial for whole body cholesterol homeostasis and reverse cholesterol transport in mice. *J Clin Invest* 122: 1688 – 1699

License: This is an open access article under the terms of the Creative Commons Attribution License, which permits use, distribution and reproduction in any medium, provided the original work is properly cited.

Expanded View Figures

Figure EV1. Inhibition of DHCR24 by SH42 does not affect food intake, body composition, organ weight, and plasma glucose and insulin levels, while increasing plasma desmosterol levels.

A *E3L.CETP* mice fed a high-fat high-cholesterol diet (HFCD) were treated with vehicle (Ctrl) or DHCR24 inhibitor SH42 (SH42) (n = 8 mice per group).
B Food intake was measured during week 3 to 6 (n = 6 and 7 cages, respectively).
C–E Body weight was measured weekly and (D) lean body mass and (E) fat body mass were determined at the end of week 8.
F After 8 weeks of treatment, mice were killed and organs were collected and weighted.
G–I Four-hour-fasted blood samples were collected before the sacrifice to measure (G) desmosterol levels and (H) glucose and (I) insulin levels.
J The homeostatic Model Assessment for Insulin Resistance (HOMA-IR) scores were calculated.

Data information: Values are mean ± SEM. Differences between two groups (SH42/Ctrl) were determined using a nonparametric Mann–Whitney test. gWAT, gonadal white adipose tissue; iBAT, interscapular brown adipose tissue.

 © 2023 The Authors

 © 2023 The Authors

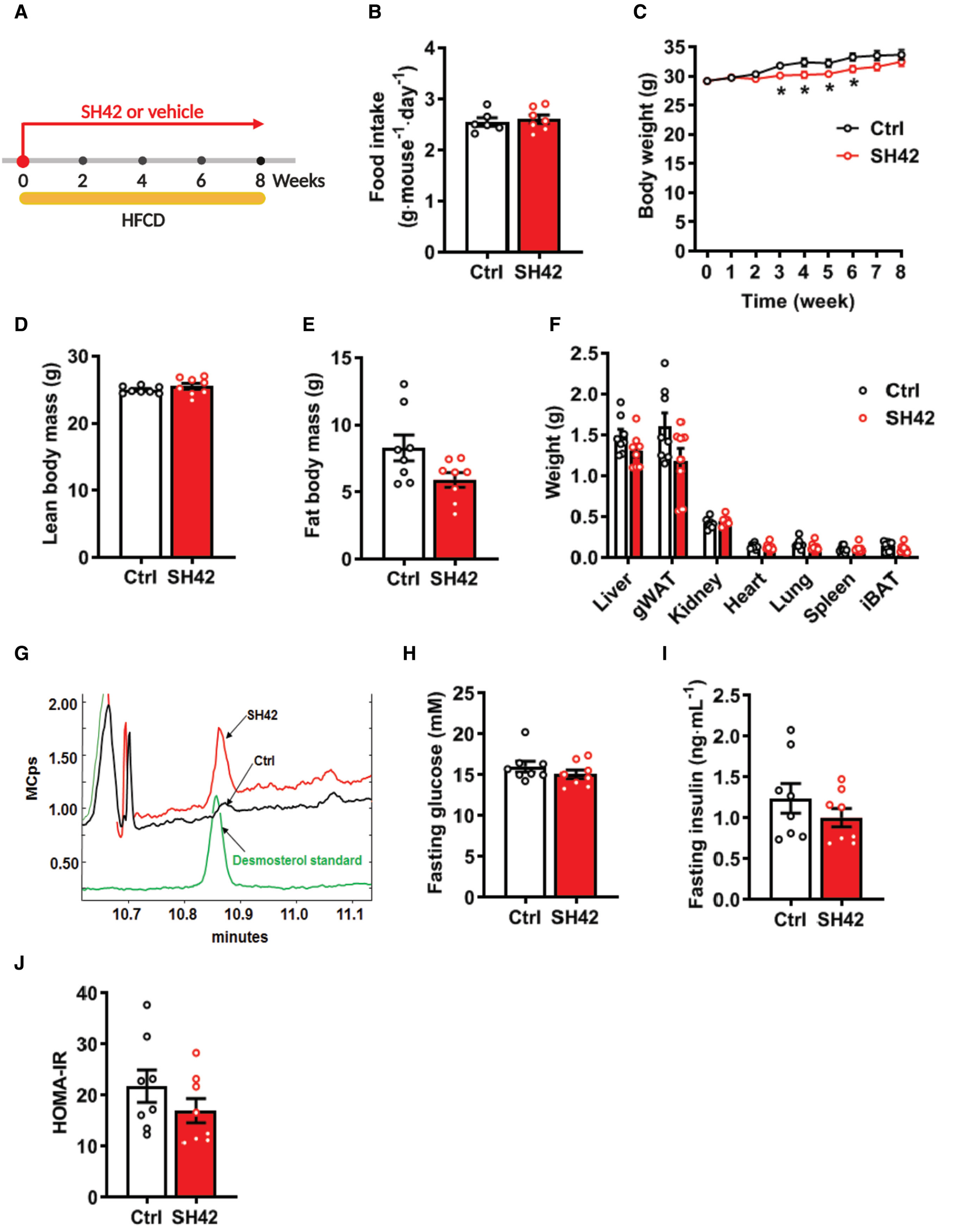
A
SH42 or vehicle
0 2 4 6 8 Weeks
HFCD
B
Food intake (g·mouse-1·day-1)
Ctrl SH42
C
Body weight (g)
Ctrl
SH42
Time (week)
D
Lean body mass (g)
Ctrl SH42
E
Fat body mass (g)
Ctrl SH42
F
Weight (g)
Ctrl
SH42
Liver gWAT Kidney Heart Lung Spleen iBAT
G
MCps
SH42
Ctrl
Desmosterol standard
minutes
H
Fasting glucose (mM)
Ctrl SH42
I
Fasting insulin (ng·mL-1)
Ctrl SH42
J
HOMA-IR
Ctrl SH42

Figure EV1.

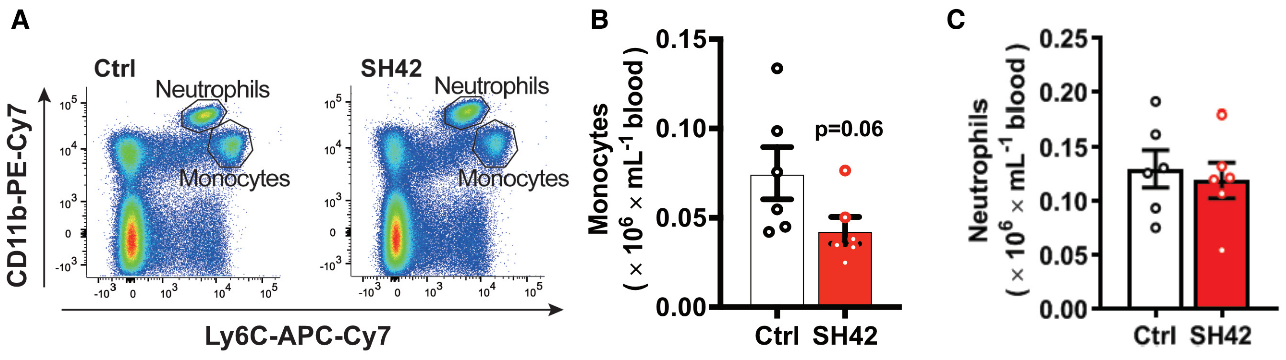

Figure EV2. Inhibition of DHCR24 by SH42 tends to reduce circulating monocytes while no effect on circulating neutrophils.

A–C *E3L.CETP* mice fed a HFCD were treated with vehicle (Ctrl) or DHCR24 inhibitor SH42 (SH42) (n = 6 mice per group). After 4 weeks of treatment, blood samples were collected to measure (A and B) monocytes and (A and C) neutrophils via flow cytometry. Values are mean ± SEM. Differences between two groups (SH42/Ctrl) were determined using a nonparametric Mann–Whitney test.

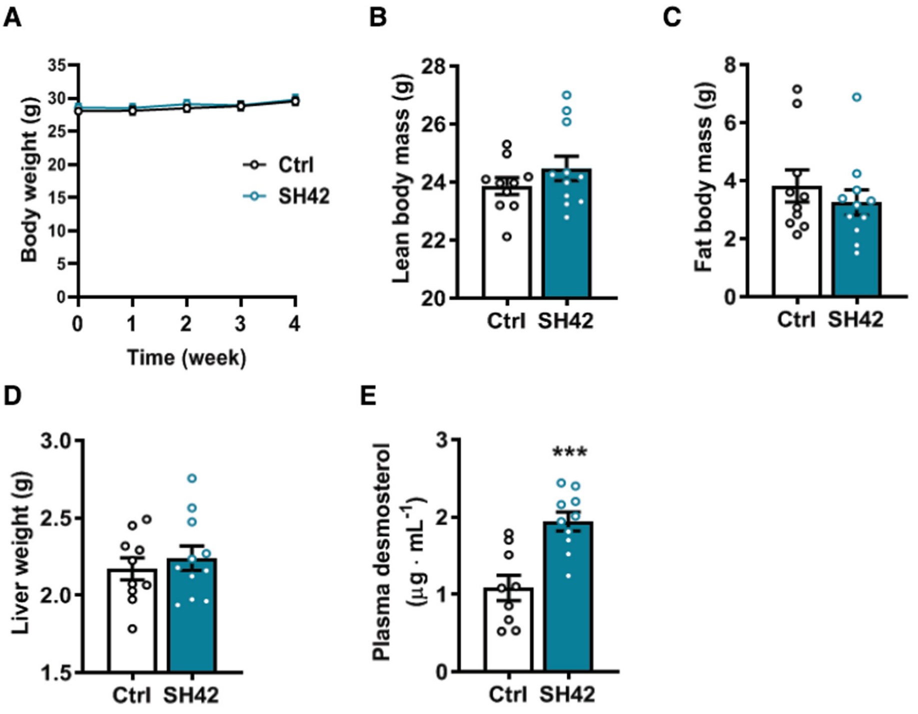

Figure EV3. Inhibition of DHCR24 by SH42 does not affect body weight, body composition or liver weight, while increasing plasma desmosterol levels in LXRα-deficient mice.

LXRα-deficient mice fed HFCD were treated with vehicle (Ctrl) or DHCR24 inhibitor SH42 (SH42) (n = 10 and 11 mice, respectively).

A Body weight was measured weekly.

B, C After 4 weeks of treatment, (B) lean body mass and (C) fat body mass were determined.

D Mice were killed and livers were collected and weighted.

E Plasma desmosterol levels were measured at the end of the experiment (n = 9 and 11 mice, respectively; one sample in control group was lost due to technical failure).

Data information: Values are mean ± SEM. Differences between two groups (SH42/Ctrl) were determined using a nonparametric Mann–Whitney test. ***$P < 0.001$ vs. ctrl.

© 2023 The Authors

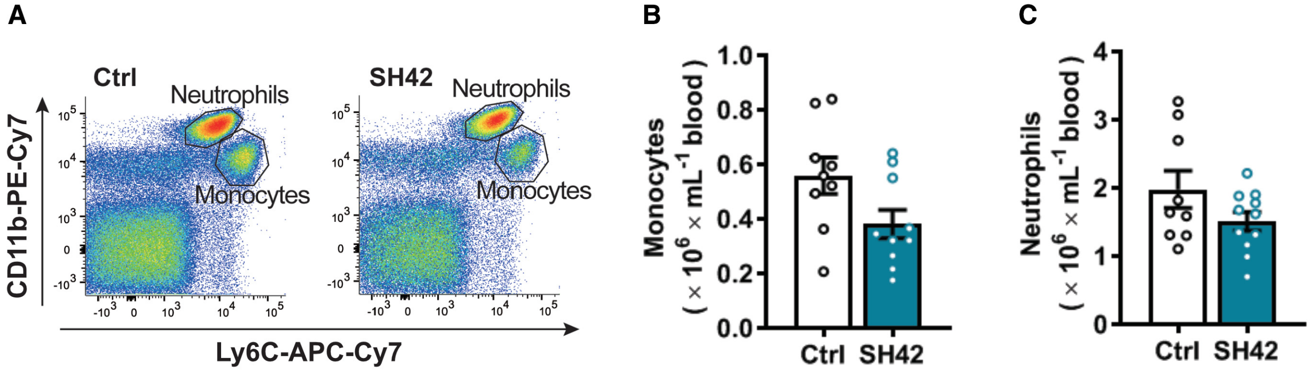

Figure EV4. Inhibition of DHCR24 by SH42 does affect circulating monocytes and neutrophils in LXRα-deficient mice.

A–C LXRα-deficient mice fed HFCD were treated with vehicle (Ctrl) or DHCR24 inhibitor SH42 (SH42) (n = 10 and 11 mice, respectively). After 4 weeks of treatment, blood samples were collected to measure (A and B) monocytes (n = 9 and 10 mice, respectively; two values were identified as outliers based on a Grubbs' test and removed from statistical analysis) and (A and C) neutrophils were determined (n = 9 and 11 mice, respectively; one value was identified as an outlier based on a Grubbs' test and removed from statistical analysis) via flow cytometry analysis. Values are mean ± SEM. Differences between two groups (SH42/Ctrl) were determined using a nonparametric Mann–Whitney test.

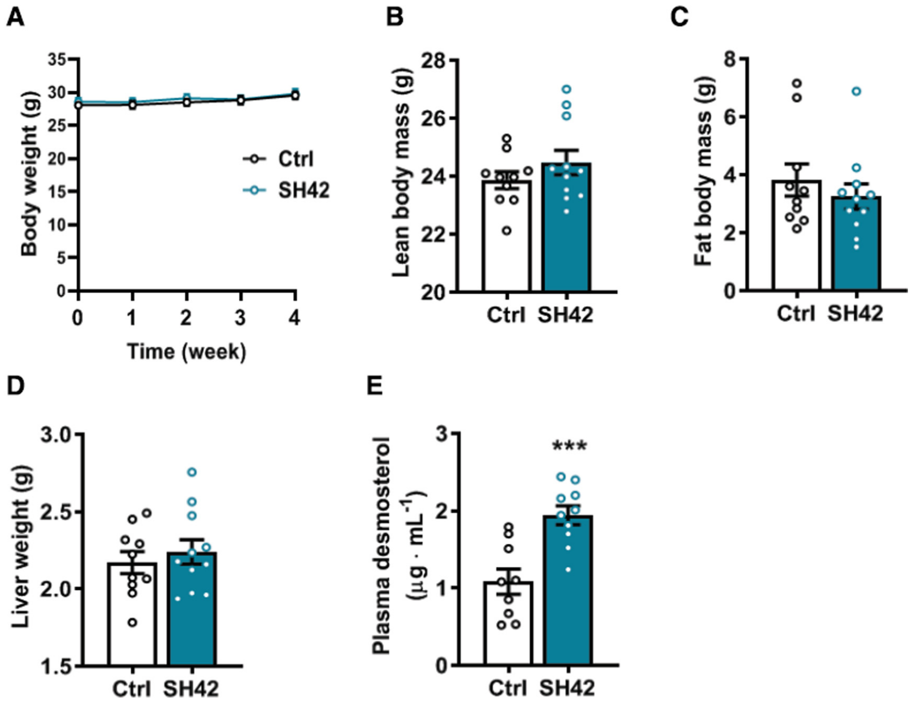

Figure EV5. Inhibition of DHCR24 by SH42 increases plasma desmosterol and does not influence body and liver weight after 10 weeks of HFCD treatment.

E3LCETP mice were fed with a HFCD for 10 weeks first and then treated with vehicle (Ctrl) or DHCR24 inhibitor SH42 (SH42) (n = 10 and 9 mice, respectively) for additional 8 weeks.

A Body weight was measured at indicated time points.
B, C After the 18 weeks, (B) lean and (C) fat body mass was evaluated.
D, E Mice were killed and (D) liver weight and (E) plasma desmosterol levels were measured.

Data information: Values are mean ± SEM. Differences between two groups (SH42/Ctrl) were determined using a nonparametric Mann–Whitney test. ***P < 0.001 vs. control (ctrl).

Zhou et al.

Inhibition of DHCR24 activates LXRα to ameliorate hepatic steatosis and inflammation

APPENDIX

Table of Contents

- **Appendix Figure S1. Inhibition of DHCR24 by SH42 alters liver lipid class compositions.** *Page 2*
- **Appendix Figure S2. Inhibition of DHCR24 by SH42 does not increase plasma DAG and TAG fractions.** *Page 3*
- **Appendix Figure S3. Representative gating scheme for FACS analysis in liver samples.** *Page 4*
- **Appendix Figure S4. Representative gating scheme for FACS analysis in blood samples.** *Page 4*
- **Appendix Figure Table S1. Antibodies used for immunohistochemistry.** *Page 5*
- **Appendix Figure Table S2. Antibodies used for flow cytometry.** *Page 10*
- **Appendix References.** *Page 5*

Appendix Figures

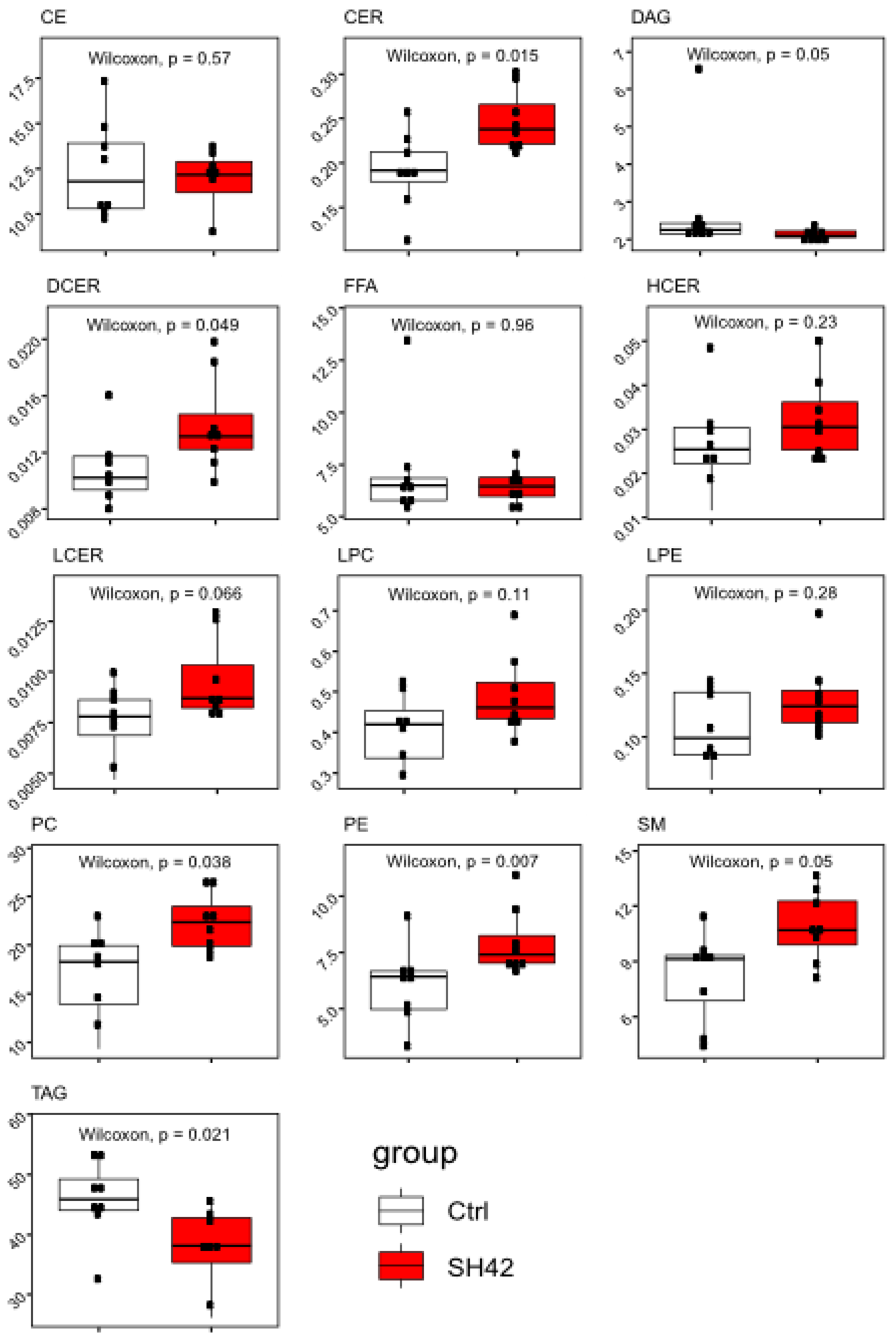

Appendix Fig. S1. Inhibition of DHCR24 by SH42 alters liver lipid class compositions.

E3L.CETP mice fed a HFCD were treated with vehicle (Ctrl) or DHCR24 inhibitor SH42 (SH42) (n= 8 mice per group). After 8 weeks of treatment, mice were killed and livers were collect to determine liver lipid class compositions. The boxplot indicates 1st quartile, median, and 3rd quartile. Differences between two groups (SH42/Ctrl) were determined using Wilcoxon rank sum test. Abbreviations: CE, cholesteryl esters; CER, ceramides; DAG, diacylglycerols; DCER, dihydroceramides; FFA, free fatty acids; HCER, hexosylceramides; LPC, lysophosphatidylcholines; LPE, lysophosphatidylethanolamines; PC, phosphatidylcholines; PE, phosphatidylethanolamines; SM, sphingomyelins; TAG, triacylglycerols.

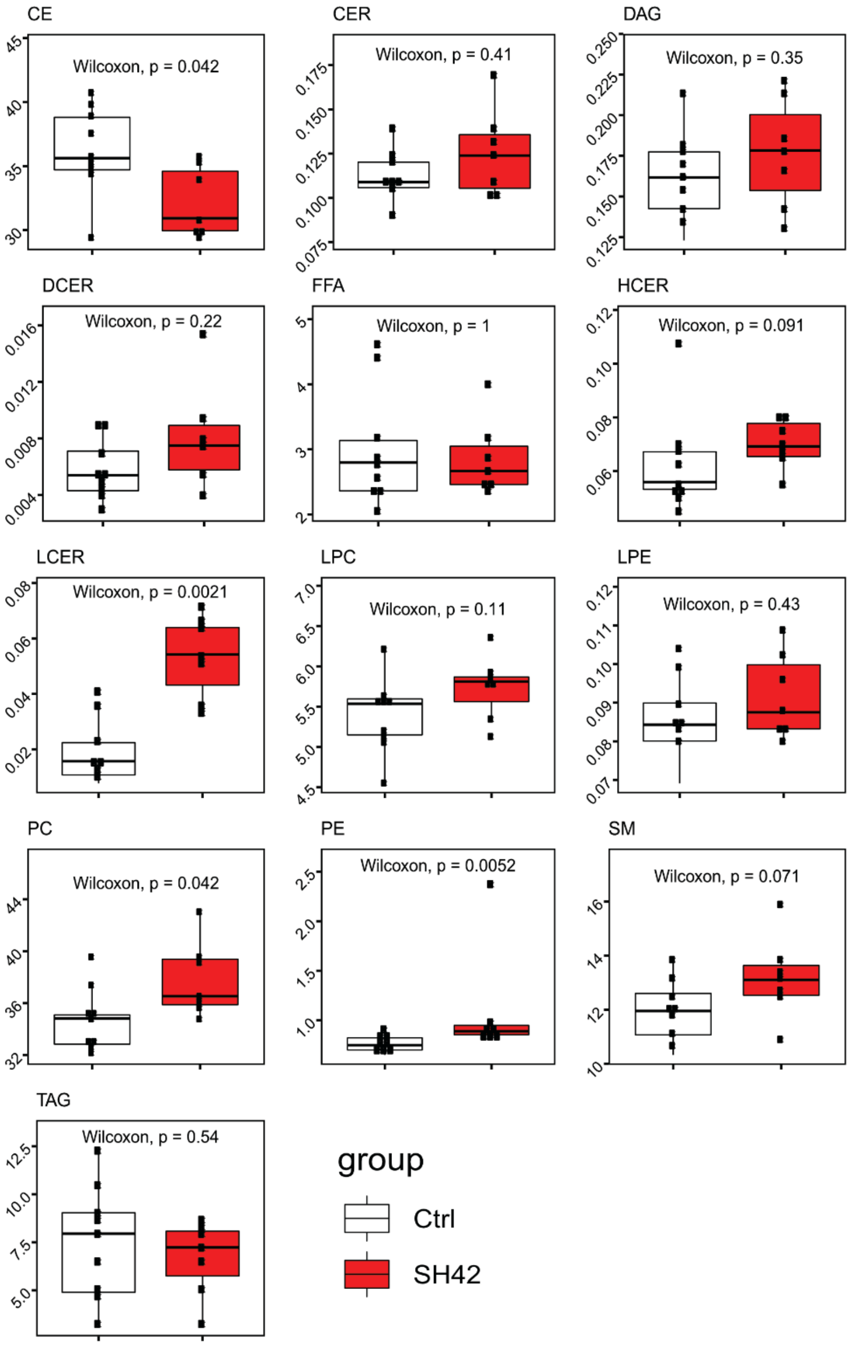

Appendix Fig. S2. Inhibition of DHCR24 by SH42 does not increase plasma DAG and TAG fractions.

E3L.CETP mice fed a HFCD were treated with vehicle (Ctrl) or DHCR24 inhibitor SH42 (SH42) (n= 8 mice per group). After 8 weeks of treatment, blood samples were collected to determine lipid class compositions. The boxplot indicates 1st quartile, median, and 3rd quartile. Differences between two groups (SH42/Ctrl) were determined using Wilcoxon rank sum test. Abbreviations: CE, cholesteryl esters; CER, ceramides; DAG, diacylglycerols; DCER, dihydroceramides; FFA, free fatty acids; HCER, hexosylceramides; LPC, lysophosphatidylcholines; LPE, lysophosphatidylethanolamines; PC, phosphatidylcholines; PE, phosphatidylethanolamines; SM, sphingomyelins; TAG, triacylglycerols.

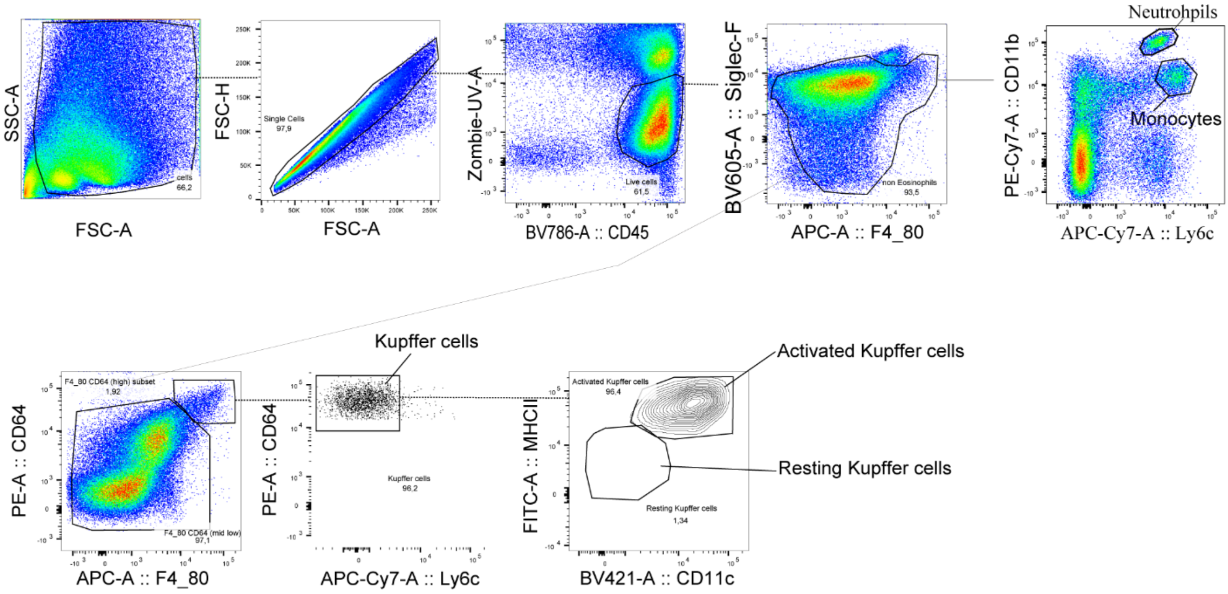

Appendix Fig. S3. Representative gating scheme for FACS analysis in liver samples.

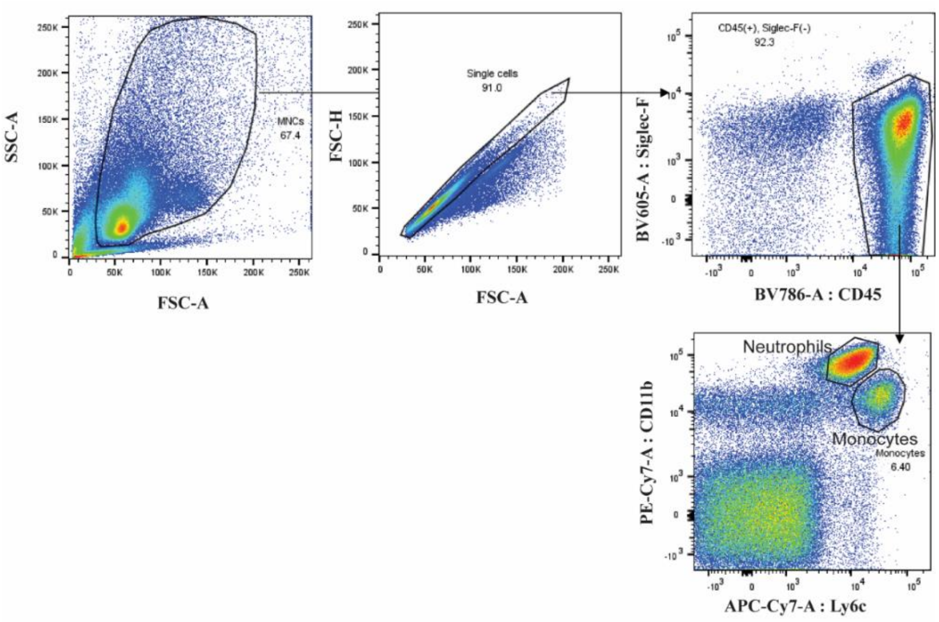

Appendix Fig. S4. Representative gating scheme for FACS analysis in blood samples.

Appendix Table S1. Antibodies used for immunohistochemistry

Target	Concentration used	Vendor	Catalog No.
F4/80	1 $\mu g \cdot mL^{-1}$	Serotec, Oxford, UK	MCA497

Appendix Table S2. Antibodies used for flow cytometry

Fluorophore	Target	Clone	Vendor
FITC	MHCII	M5/114.15.2	Biolegend
PE	CD64	X54-5/7.1	Biolegend
PE-Cy7	CD11b	M1/70	eBioscience
APC	F4/80	BM8	eBioscience
APC-Cy7	Ly6C	HK1.4	Biolegend
BV421	CD11c	N418	Biolegend
BV605	Siglec-F	E50-2440	BD Biosciences
BV785	CD45	30F11	Biolegend
