# Supplementary figures and images for: Inhibition of DHCR24 activates LXRα to ameliorate hepatic steatosis and inflammation
Source: EMBO Mol Med. 2023 Jun 26;15(8):e16845. doi: 10.15252/emmm.202216845 (PMC10405065; doi:10.15252/emmm.202216845)

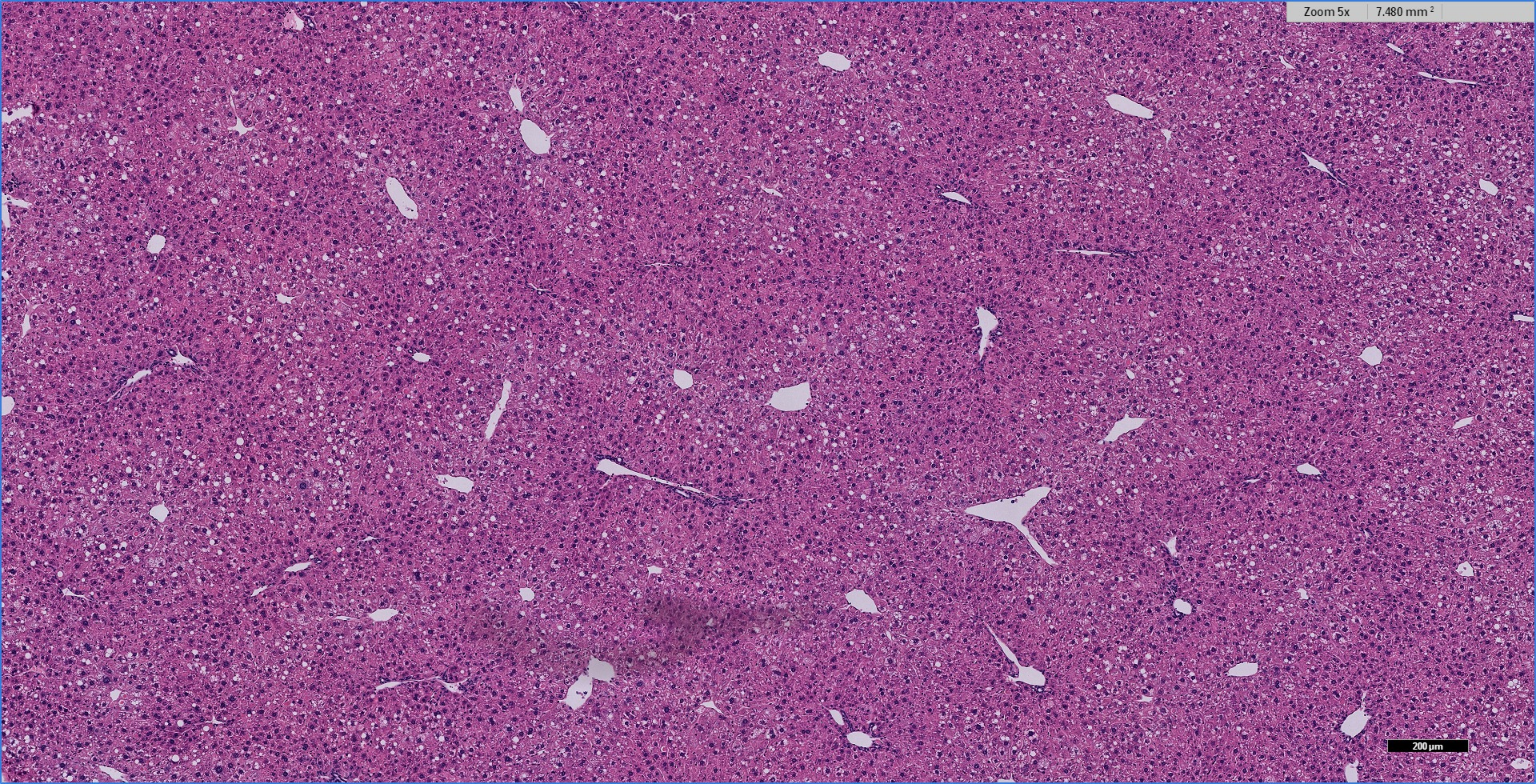

Supplement: Supplementary file 4 — Source Data for Figure 1 [file EMMM-15-e16845-s010.zip › Figure 1/1B/1B_SH42_HE.pdf]

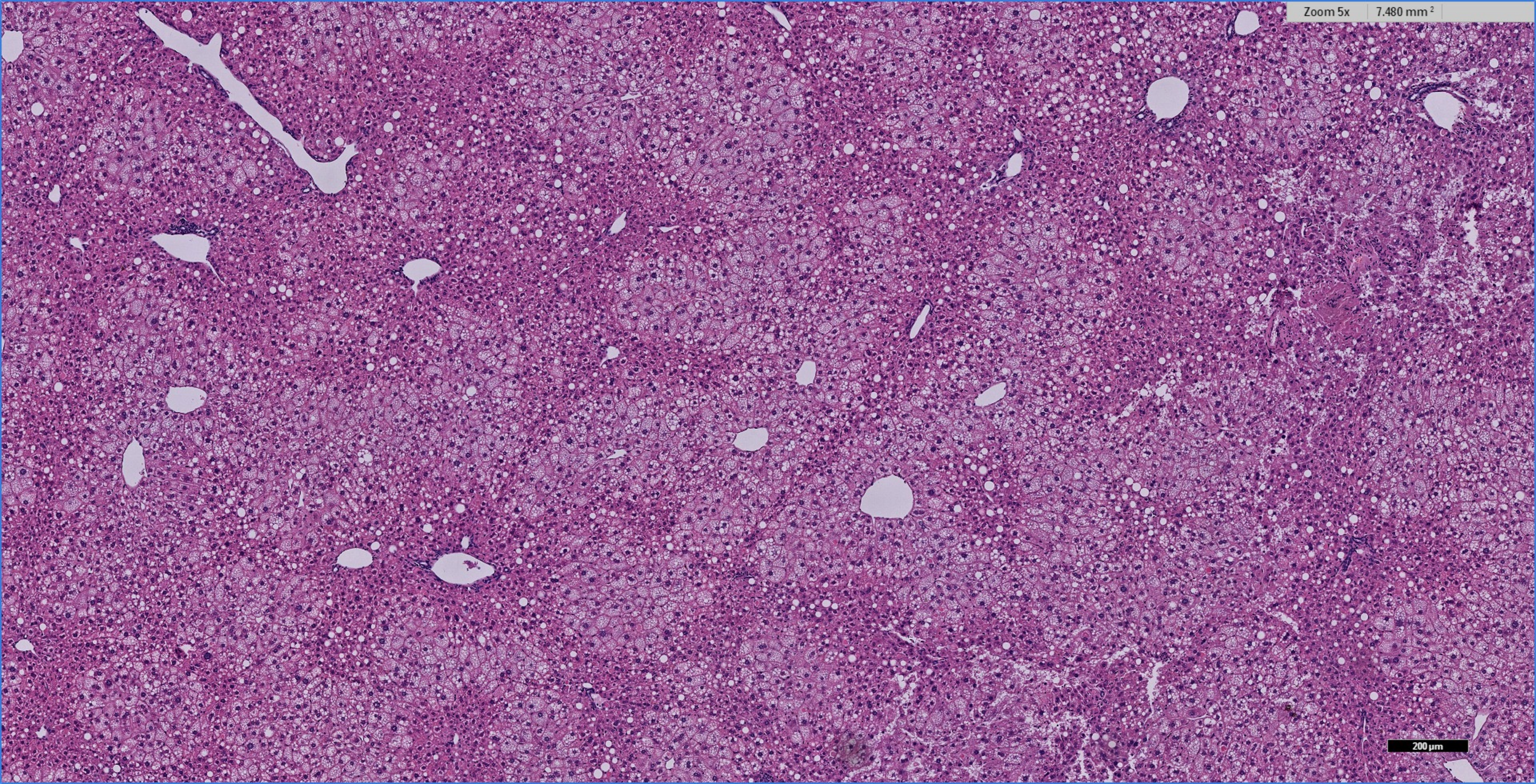

Supplement: Supplementary file 4 — Source Data for Figure 1 [file EMMM-15-e16845-s010.zip › Figure 1/1B/1B_Ctrl_HE.pdf]

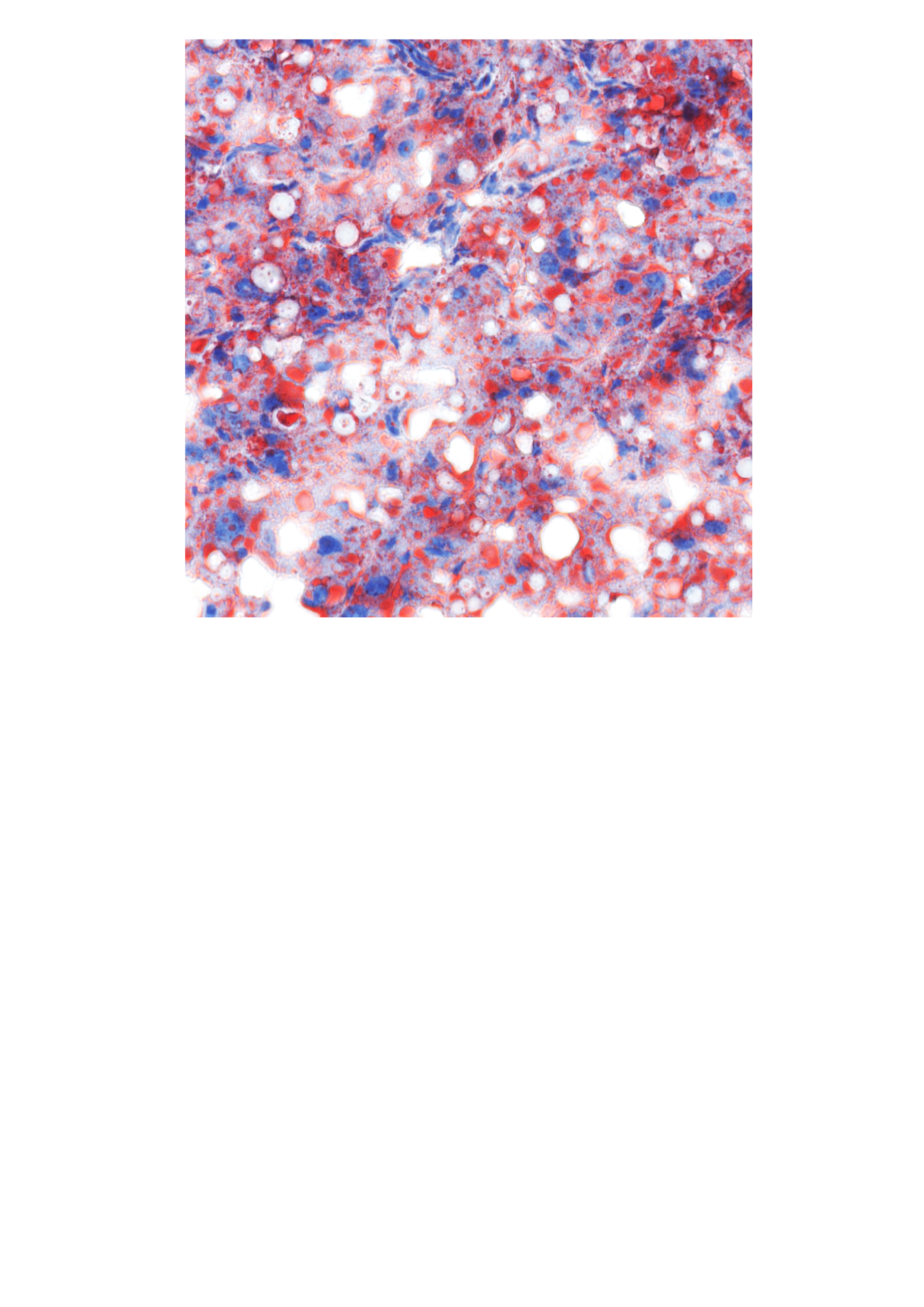

Supplement: Supplementary file 4 — Source Data for Figure 1 [file EMMM-15-e16845-s010.zip › Figure 1/1B/1B_SH42_Oil Red O.tiff]

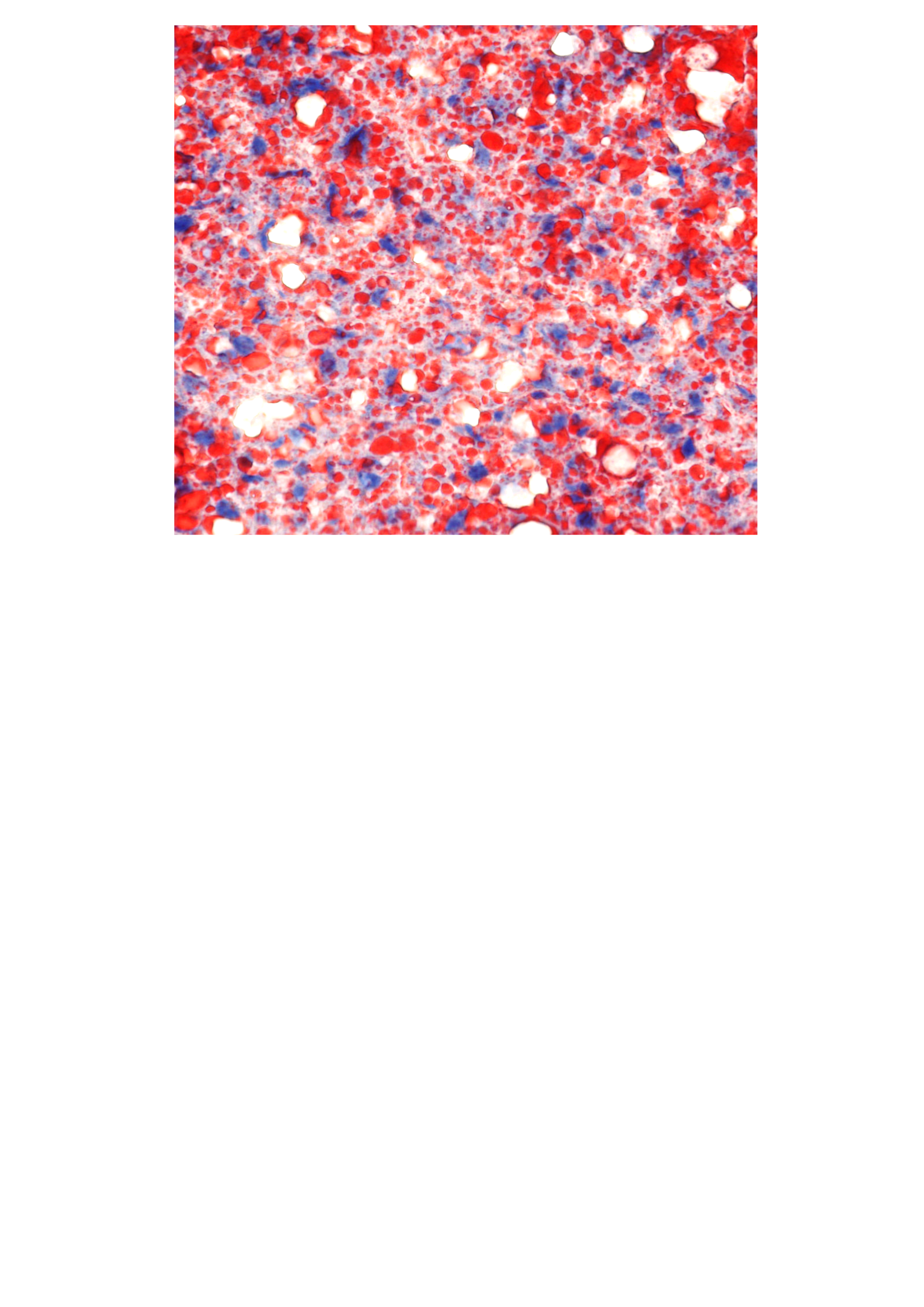

Supplement: Supplementary file 4 — Source Data for Figure 1 [file EMMM-15-e16845-s010.zip › Figure 1/1B/1B_Ctrl_Oil Red O.tiff]

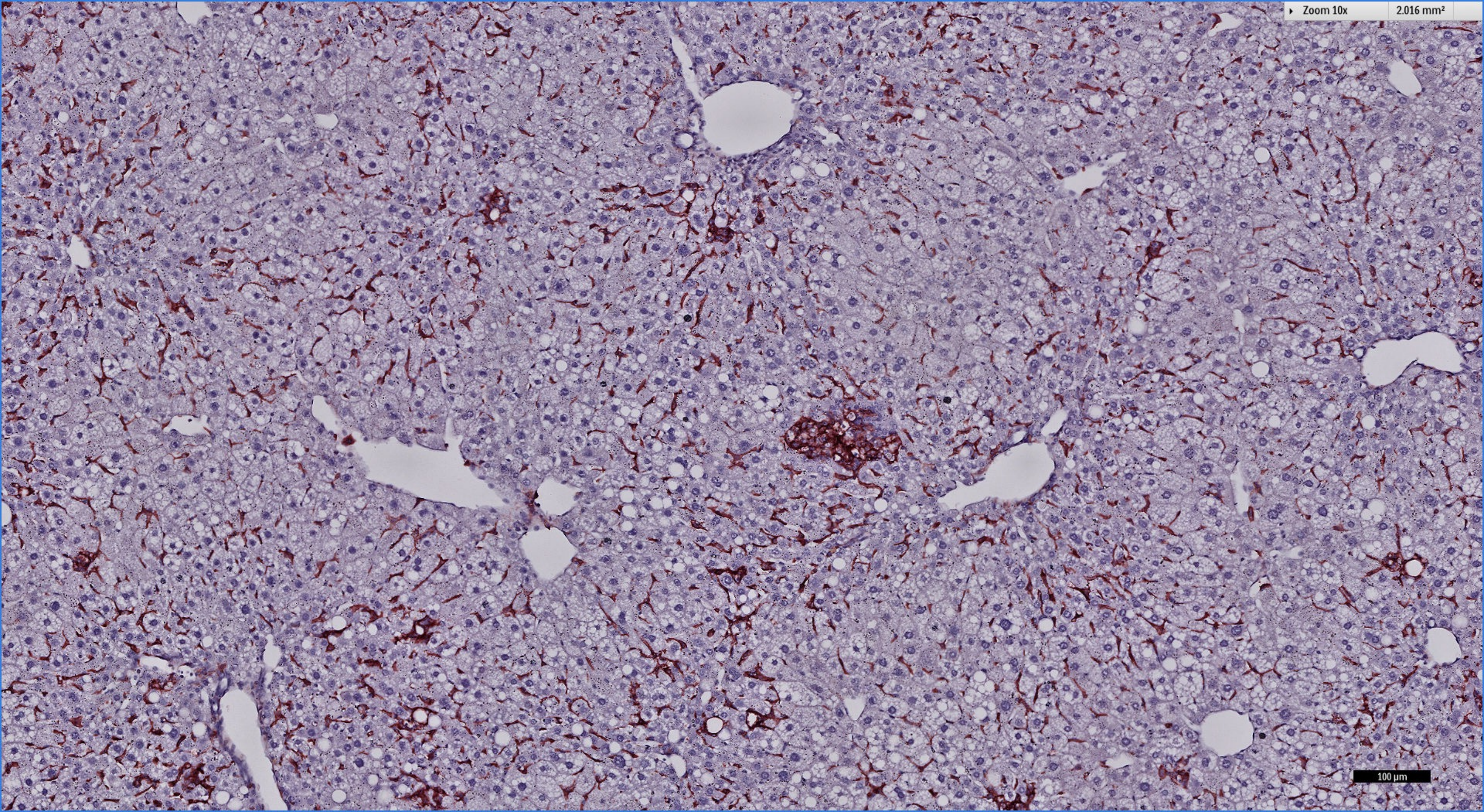

Supplement: Supplementary file 5 — Source Data for Figure 2 [file EMMM-15-e16845-s006.zip › Figure 2/2A/2A_Ctrl.pdf]

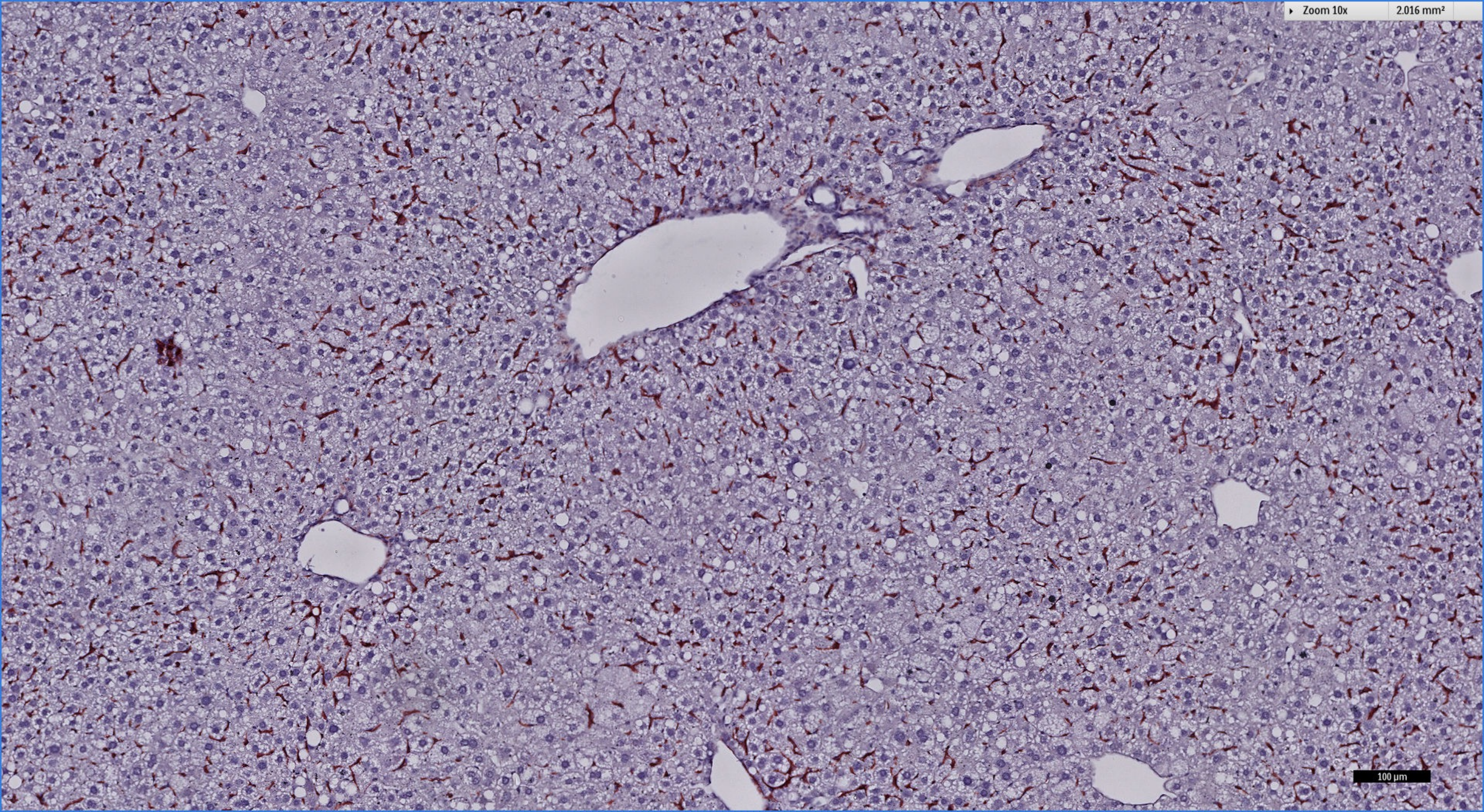

Supplement: Supplementary file 5 — Source Data for Figure 2 [file EMMM-15-e16845-s006.zip › Figure 2/2A/2A_SH42.pdf]

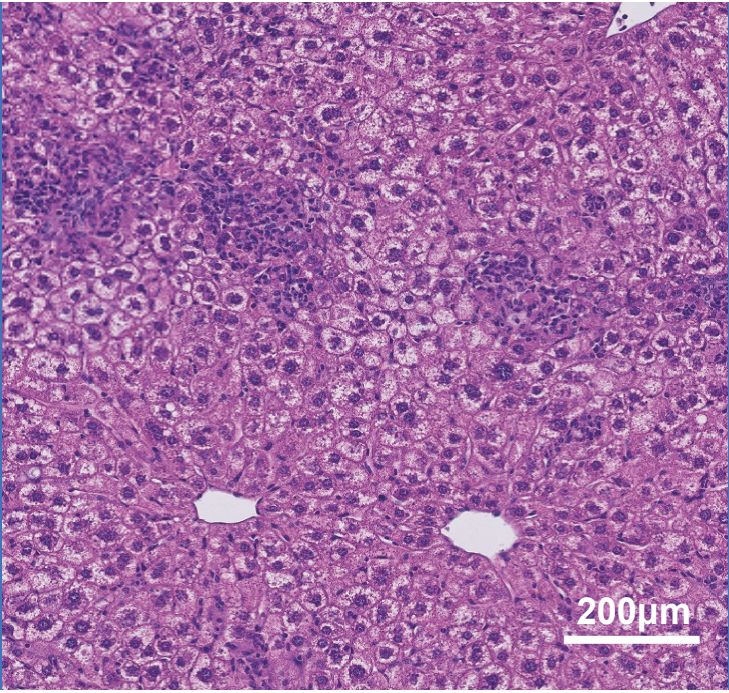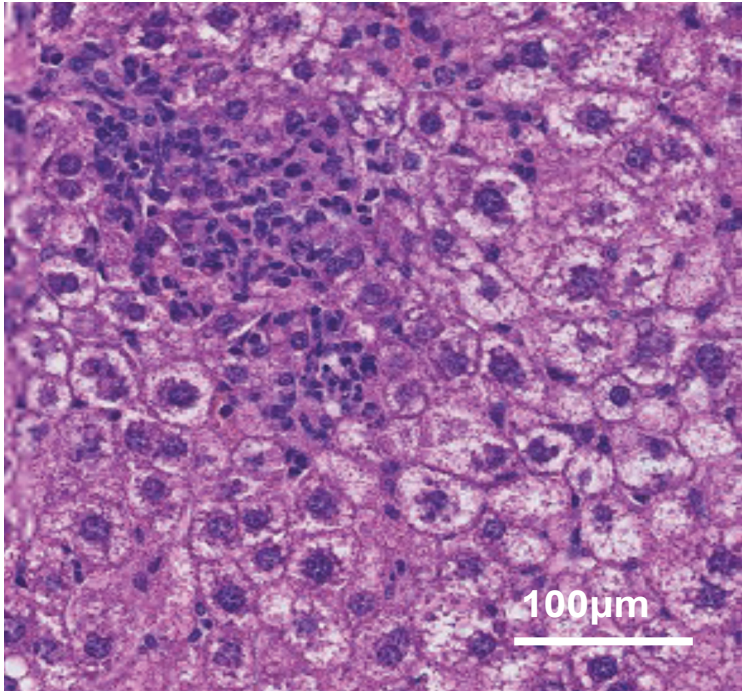

Supplement: Supplementary file 7 — Source Data for Figure 4 [file EMMM-15-e16845-s008.zip › Figure 4/4A/4A_HE_SH42.pdf]

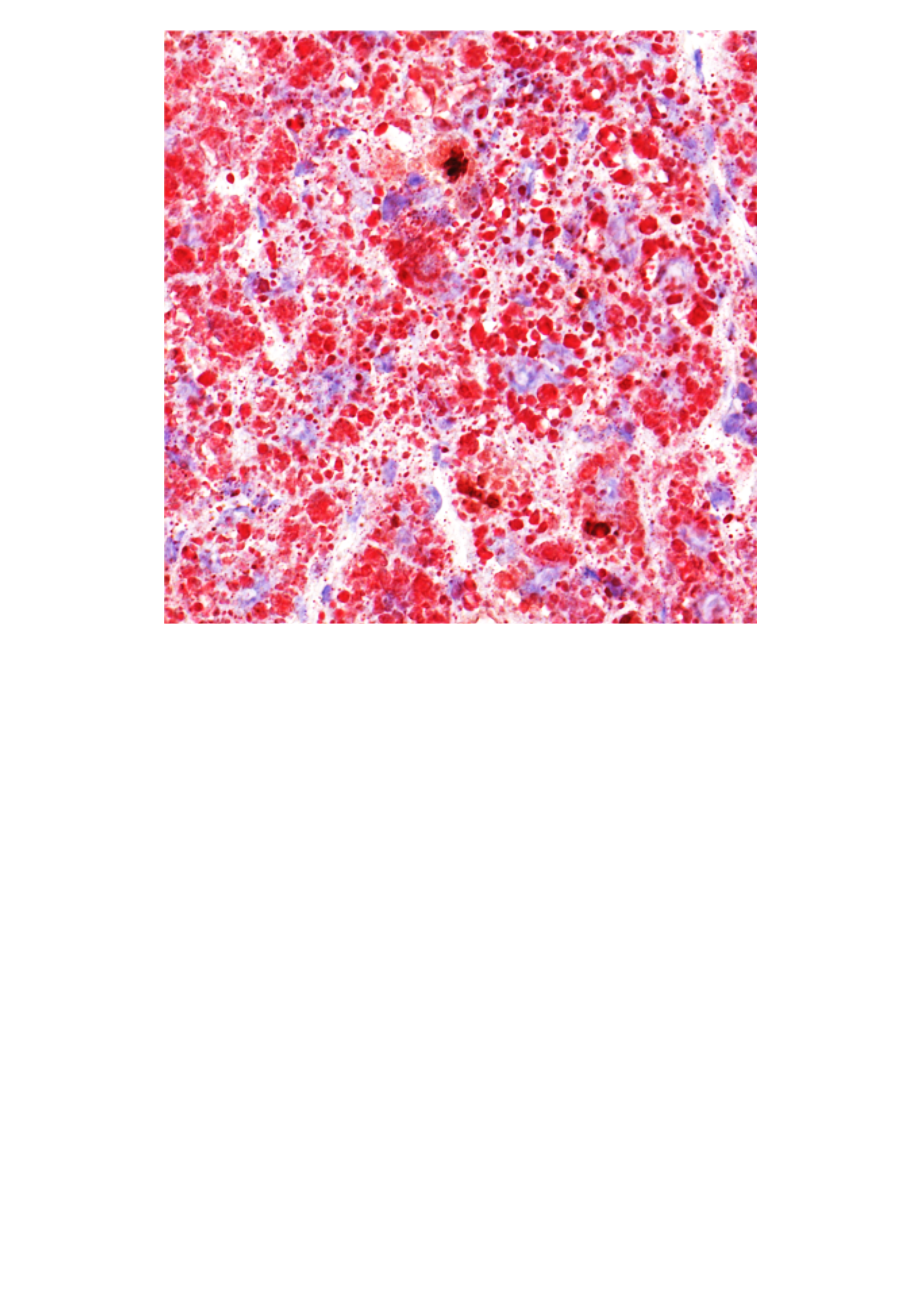

Supplement: Supplementary file 7 — Source Data for Figure 4 [file EMMM-15-e16845-s008.zip › Figure 4/4A/4A_Oil Red O_SH42.tiff]

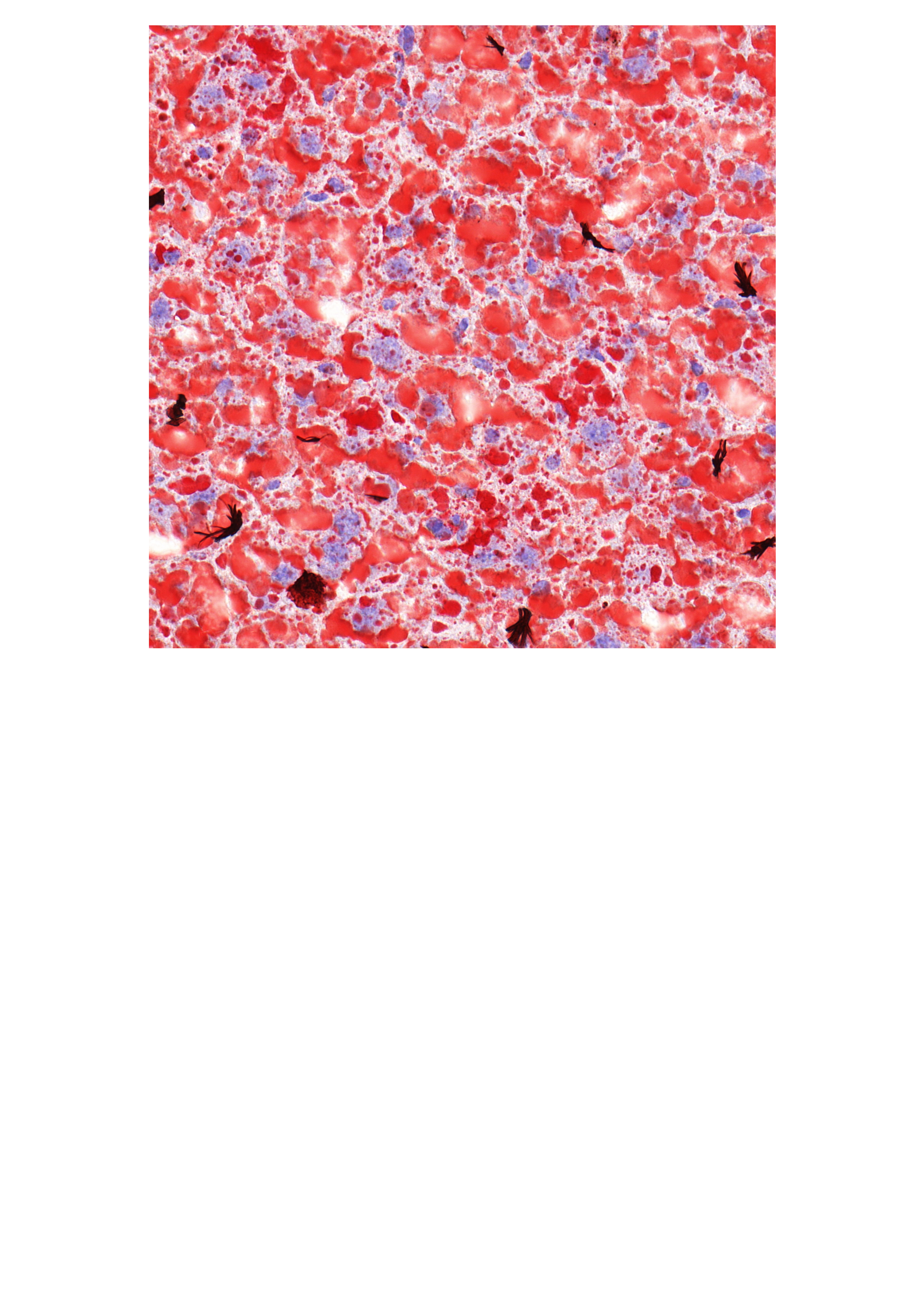

Supplement: Supplementary file 7 — Source Data for Figure 4 [file EMMM-15-e16845-s008.zip › Figure 4/4A/4A_Oil Red O_Ctrl.tiff]

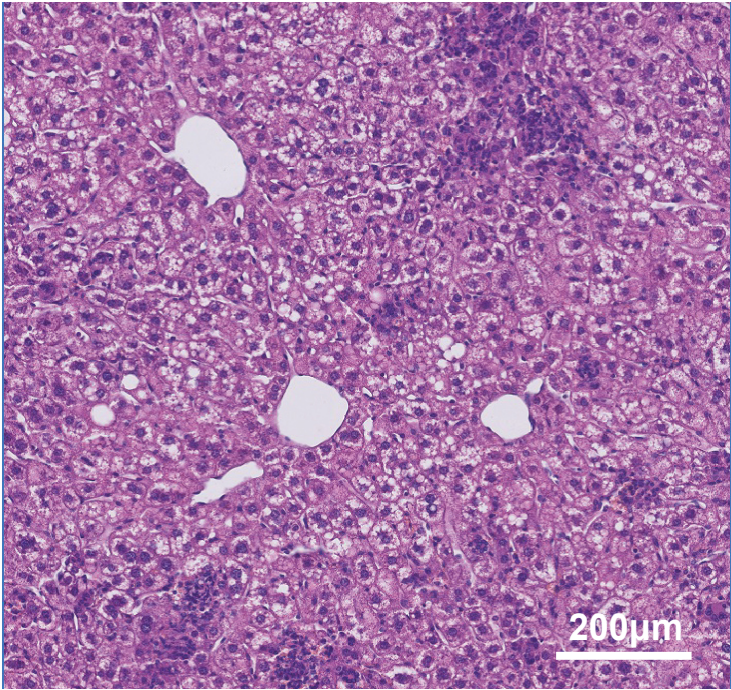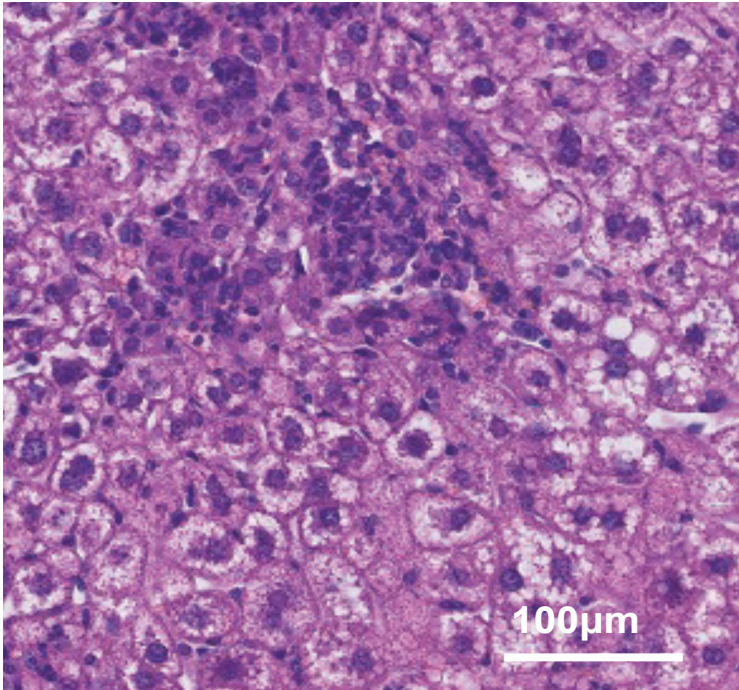

Supplement: Supplementary file 7 — Source Data for Figure 4 [file EMMM-15-e16845-s008.zip › Figure 4/4A/4A_HE_Ctrl.pdf]

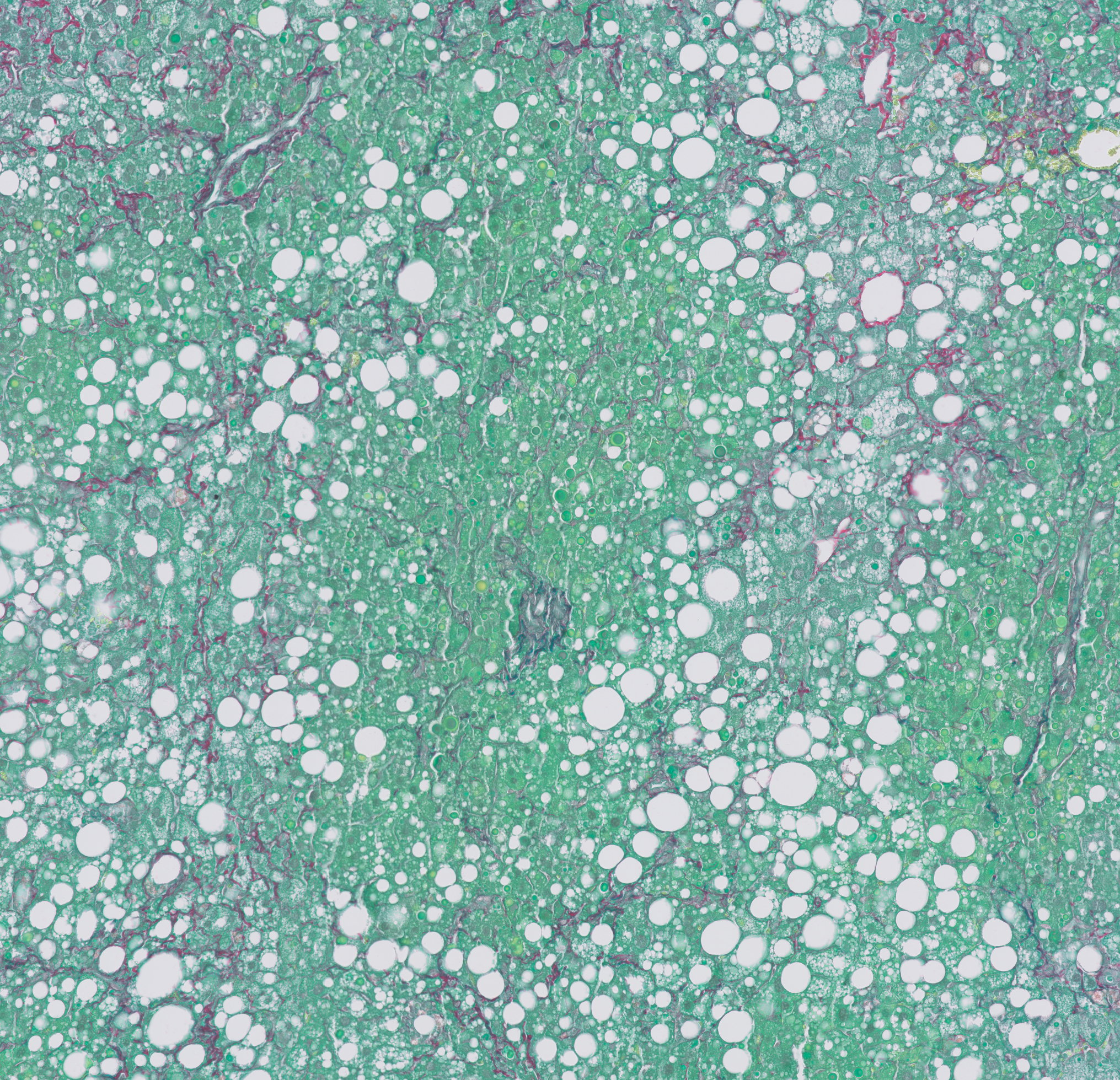

Supplement: Supplementary file 9 — Source Data for Figure 6 [file EMMM-15-e16845-s003.zip › Figure 6/6H/6H_Ctrl.png]

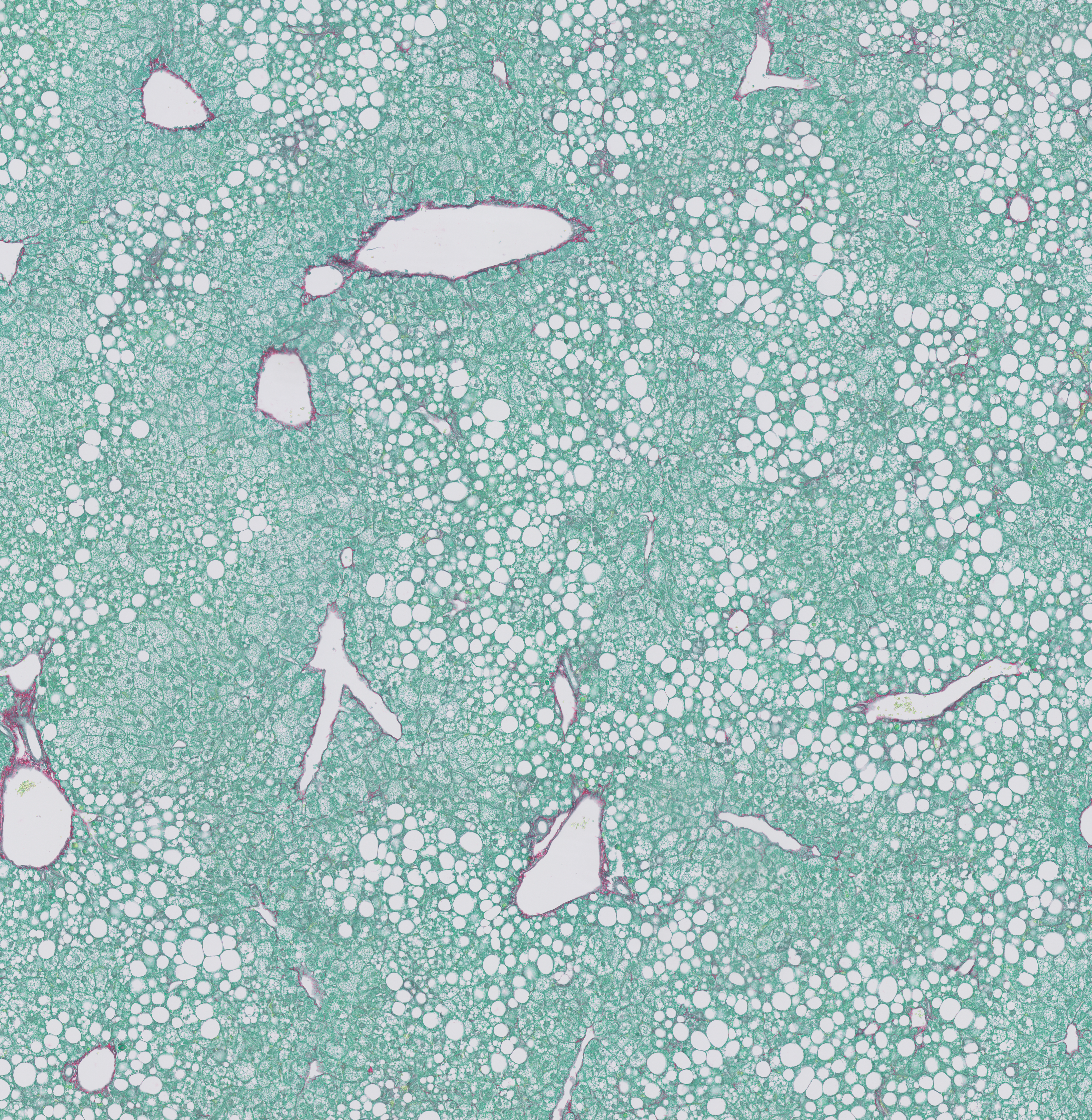

Supplement: Supplementary file 9 — Source Data for Figure 6 [file EMMM-15-e16845-s003.zip › Figure 6/6H/6H_SH42.png]

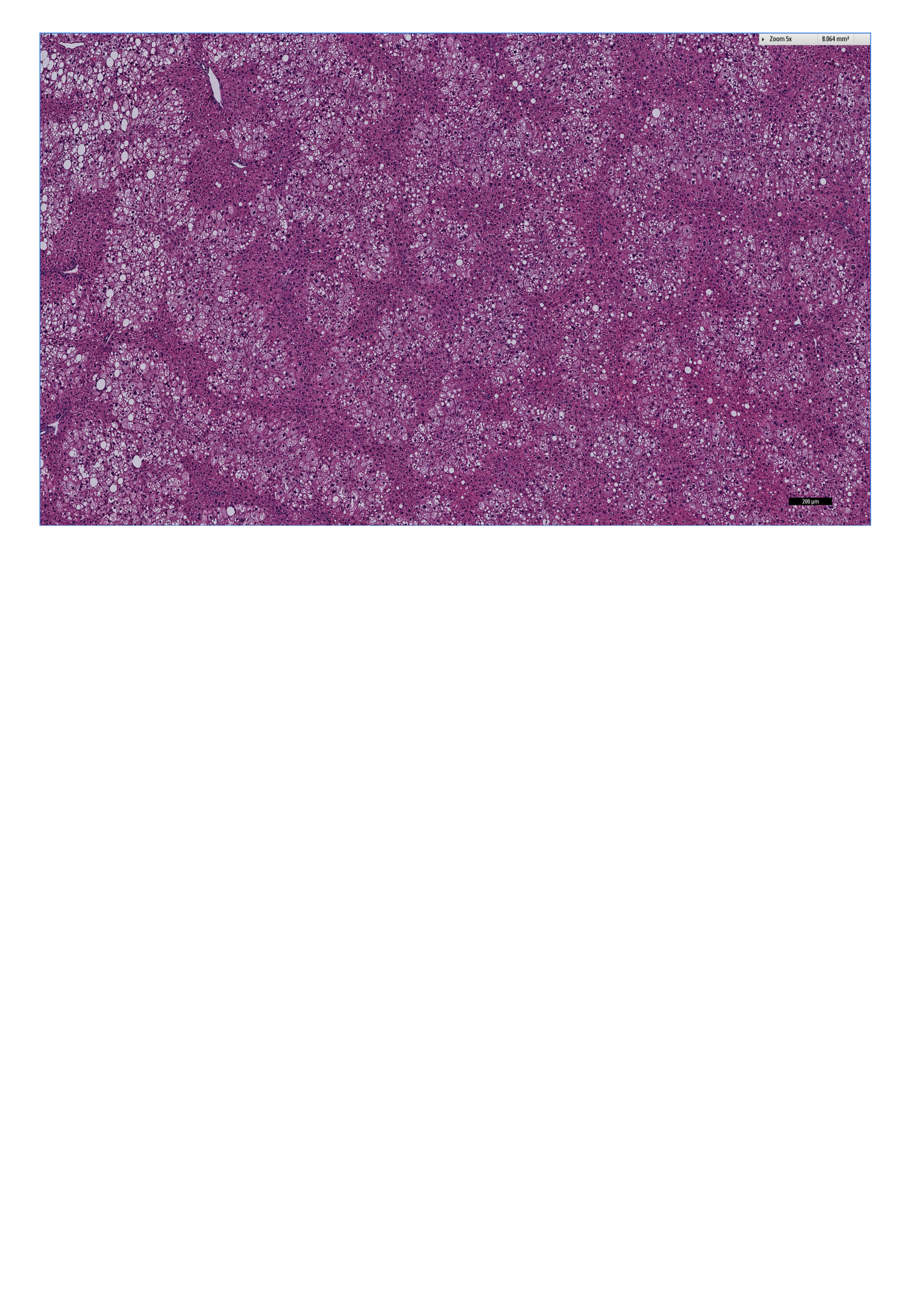

Supplement: Supplementary file 9 — Source Data for Figure 6 [file EMMM-15-e16845-s003.zip › Figure 6/6B/6B_HE_SH42.tiff]

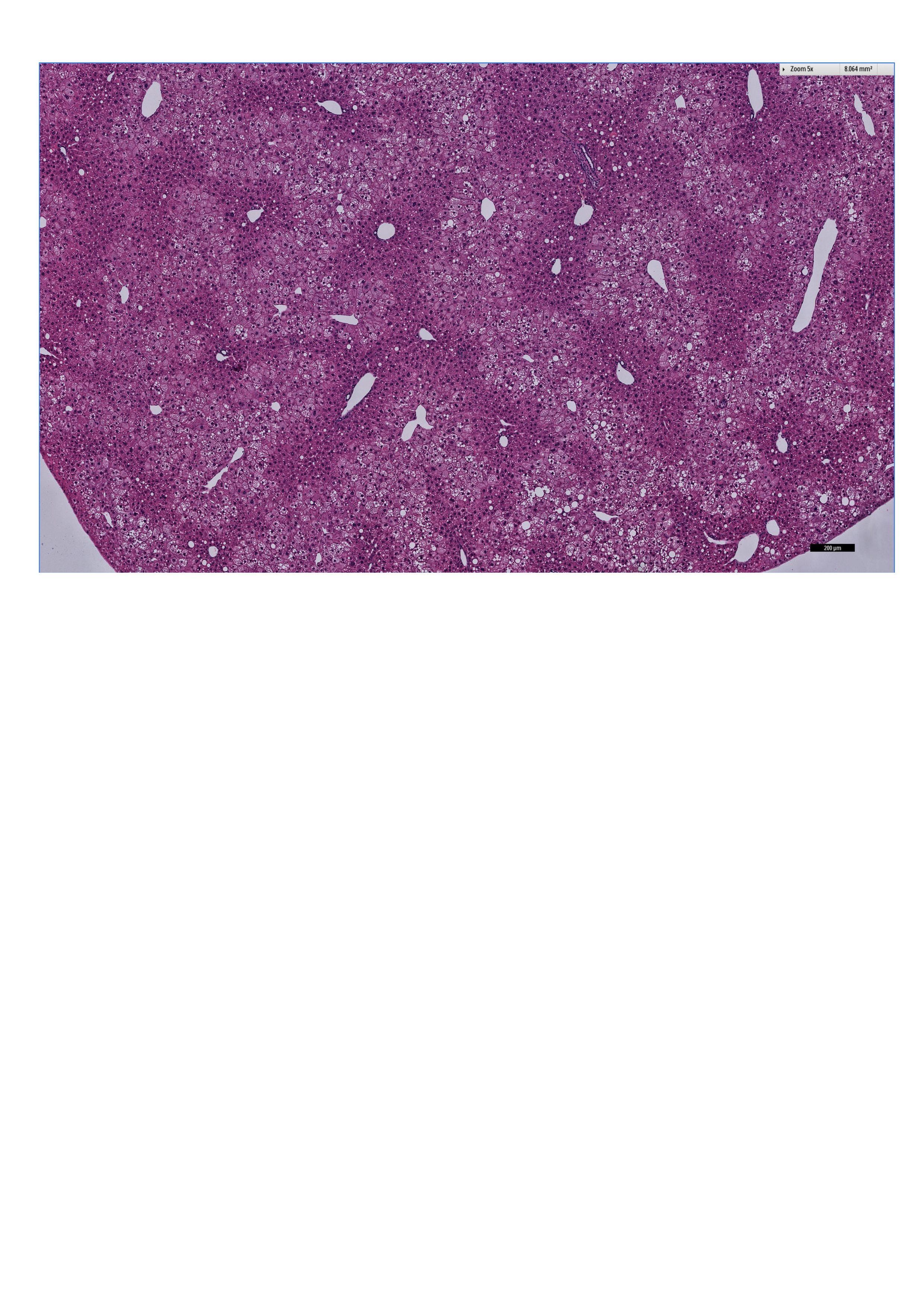

Supplement: Supplementary file 9 — Source Data for Figure 6 [file EMMM-15-e16845-s003.zip › Figure 6/6B/6B_HE_Ctrl.tiff]

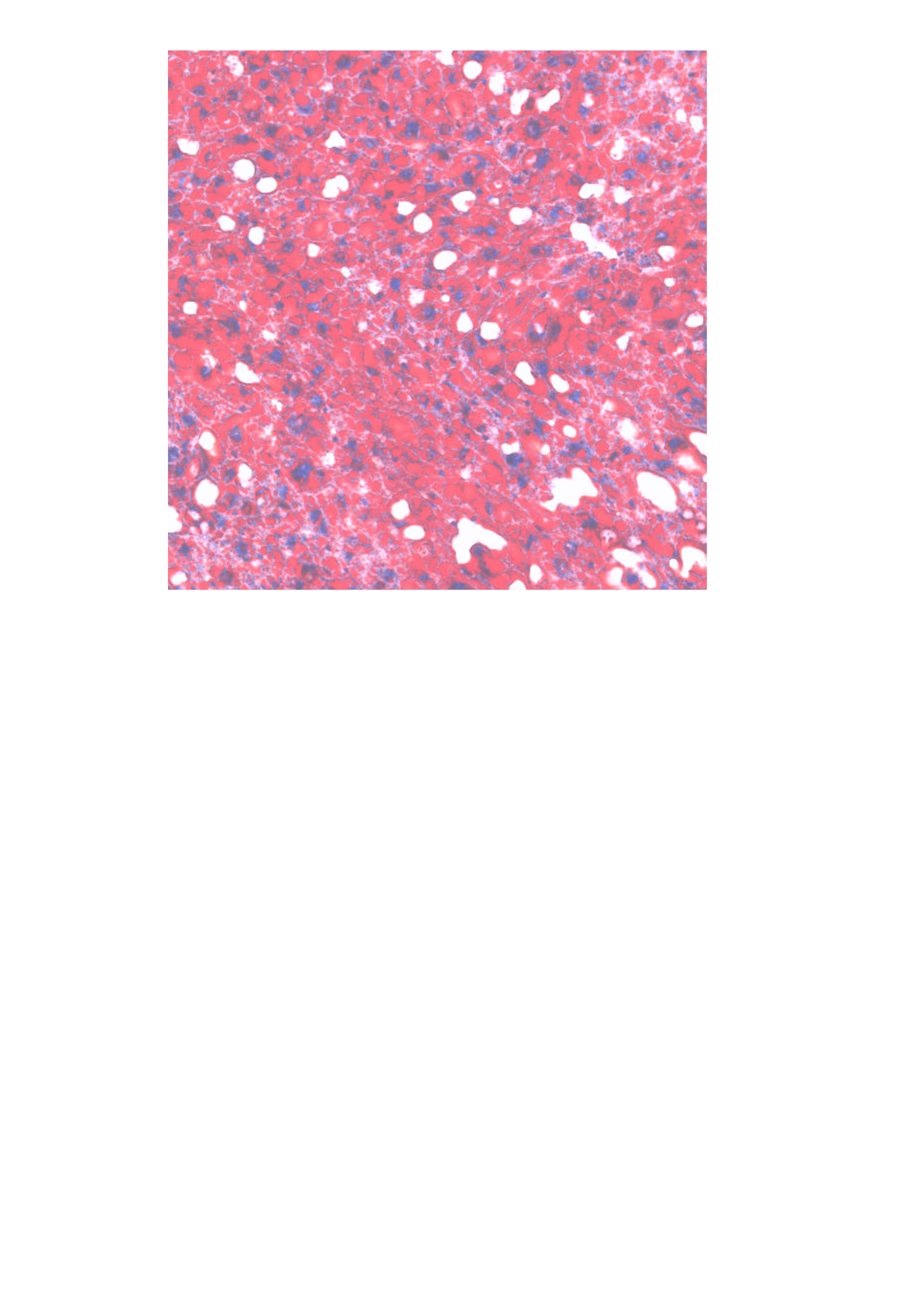

Supplement: Supplementary file 9 — Source Data for Figure 6 [file EMMM-15-e16845-s003.zip › Figure 6/6B/6B_Oil Red O_Ctrl.tiff]

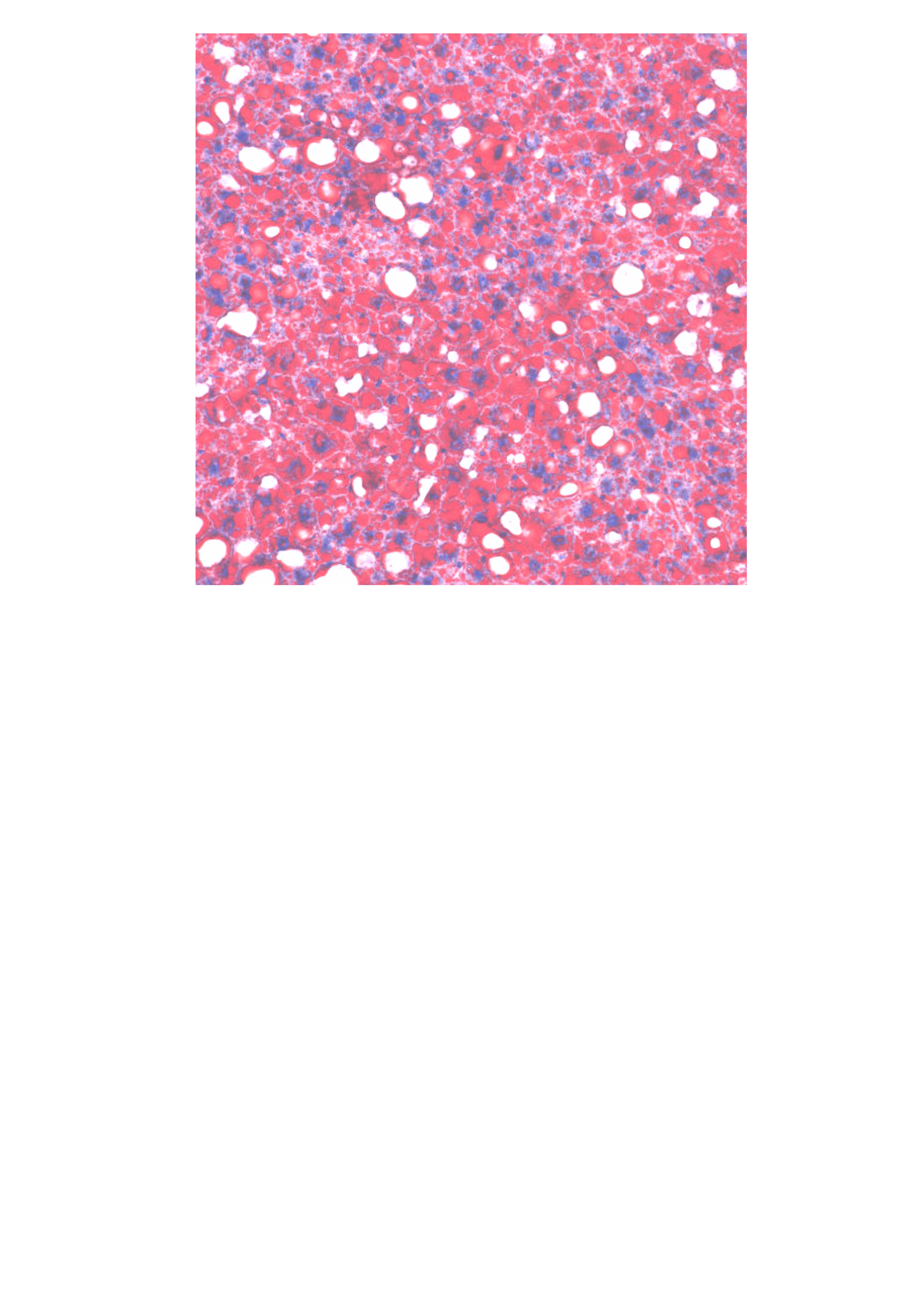

Supplement: Supplementary file 9 — Source Data for Figure 6 [file EMMM-15-e16845-s003.zip › Figure 6/6B/6B_Oil Red O_SH42.tiff]

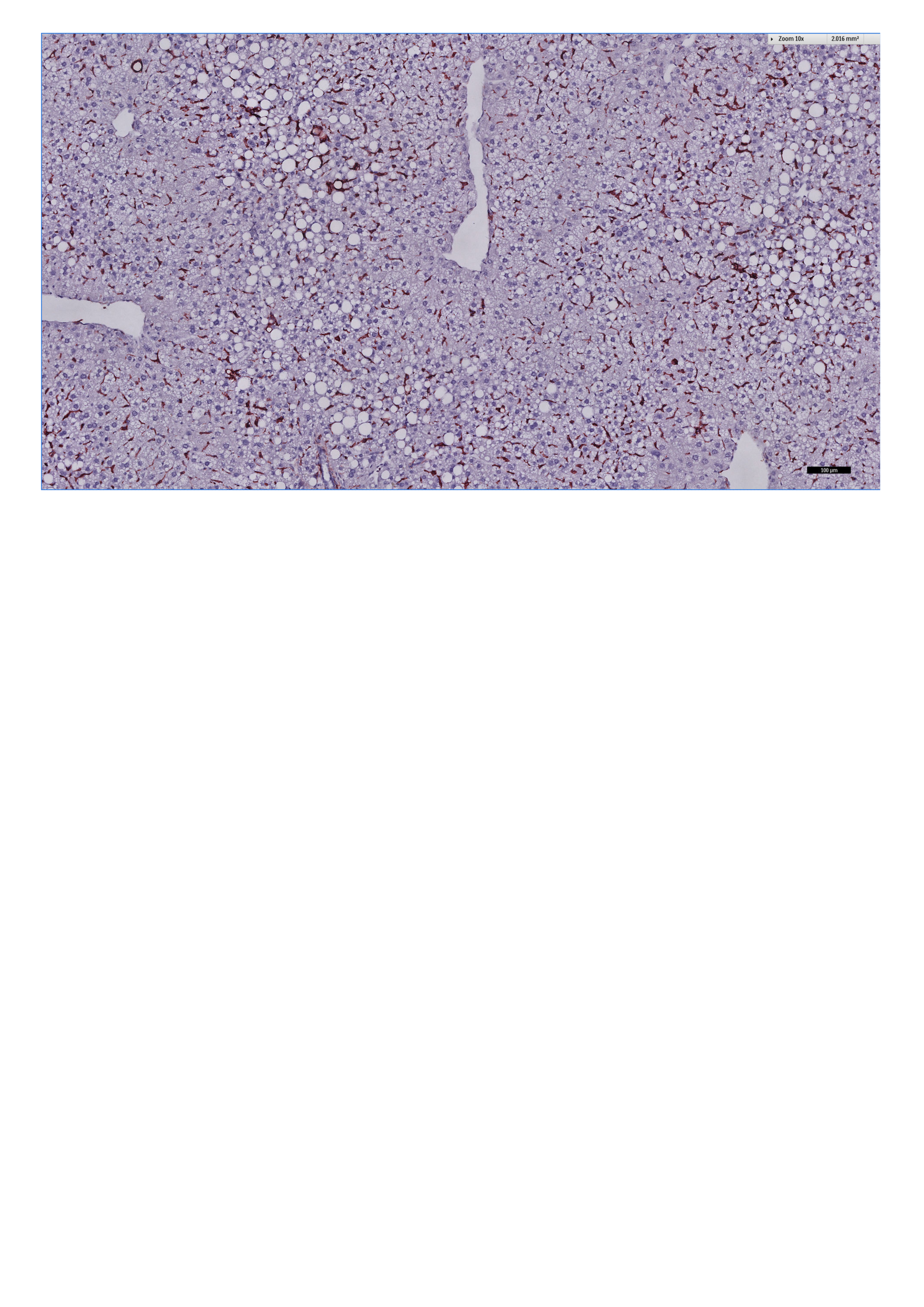

Supplement: Supplementary file 9 — Source Data for Figure 6 [file EMMM-15-e16845-s003.zip › Figure 6/6E/6E_SH42.tiff]

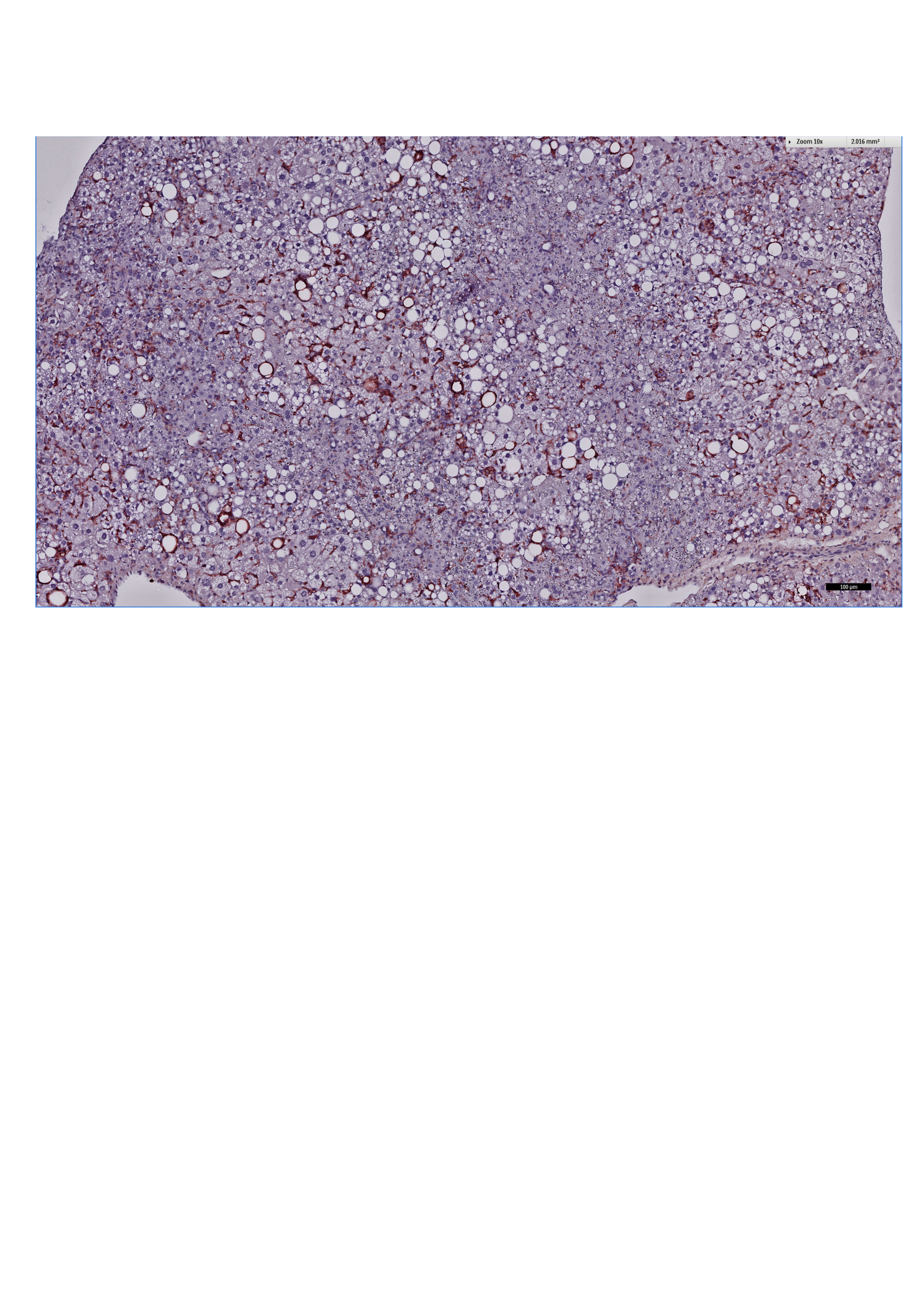

Supplement: Supplementary file 9 — Source Data for Figure 6 [file EMMM-15-e16845-s003.zip › Figure 6/6E/6E_Ctrl.tiff]
