# Supplementary material for: Inhibition of DHCR24 activates LXRα to ameliorate hepatic steatosis and inflammation
Source: EMBO Mol Med. 2023 Jun 26;15(8):e16845. doi: 10.15252/emmm.202216845 (PMC10405065; doi:10.15252/emmm.202216845)
Supplement: Supplementary file 10 [file EMMM-15-e16845-s001.pdf]

EMBO Molecular Medicine

Inhibition of DHCR24 activates LXRα to ameliorate hepatic steatosis and inflammation

Enchen Zhou Zhou, Xiaoke Ge, Hiroyuki Nakashima, Rumei Li, Hendrik J.P. van der Zande, Cong Liu, Zhuang Li, Christoph Müller, Franz Bracher, Yassene Mohammed, Jan Freark de Boer, Folkert Kuipers, Bruno Guigas, Christopher Glass, Patrick Rensen, Martin Giera, and Yanan Wang
DOI: 10.15252/emmm.202216845

Corresponding author(s): Yanan Wang (yanan.wang@xjtu.edu.cn) , Yanan Wang (yanan.wang@xjtu.edu.cn)

Review Timeline:

Submission Date:	7th Oct 22
Editorial Decision:	15th Nov 22
Appeal Received:	5th Dec 22
Editorial Decision:	7th Dec 22
Revision Received:	28th Feb 23
Editorial Decision:	22nd Mar 23
Revision Received:	8th May 23
Editorial Decision:	23rd May 23
Revision Received:	6th Jun 23
Accepted:	7th Jun 23

Editor: Zeljko Durdevic

Transaction Report:

(Note: With the exception of the correction of typographical or spelling errors that could be a source of ambiguity, letters and reports are not edited. Depending on transfer agreements, referee reports obtained elsewhere may or may not be included in this compilation. Referee reports are anonymous unless the Referee chooses to sign their reports.)

15th Nov 2022

Dear Dr. Wang,

Thank you for the submission of your manuscript to EMBO Molecular Medicine, and please accept my apologies for the delay in getting back to you. We have now received feedback from two of the three reviewers who agreed to evaluate your manuscript. As the referee #3 will unfortunately not be able to return his/her report in a timely manner, and given that both reviewers provide very similar recommendations, we prefer to make a decision now in order to avoid further delay in the process.

As you will see from their reports pasted below, while they recognize interest of your study, they also raise serious concerns, particularly regarding the lack of evidence to support the main conclusions of the study. Given the nature of these criticisms, addressing all the referees' comments would require a lot of additional work, time, and effort. As clear and conclusive insight into a novel, clinically relevant observation is crucial for publication in EMBO Molecular Medicine, and together with the fact that we only accept papers that receive enthusiastic support upon initial review, I am afraid that we cannot offer to consider the manuscript further.

Given the potential interest and novelty of the findings, we would, however, be willing to consider a new manuscript on the same topic if at some time in the near future you obtained data that would considerably strengthen the message of the study and address the referees concerns in full. To be completely clear, however, I would like to stress that if you were to send a new manuscript this would be treated as a new submission rather than a revision and would be reviewed afresh, in particular with respect to the literature and the novelty of your findings at the time of resubmission. If you decide to follow this route, please make sure you nevertheless upload a letter of response to the referees' comments.

I am sorry that I could not bring better news this time and hope that the referee comments are helpful in your continued work in this area.

Yours sincerely,

Zeljko Durdevic

Zeljko Durdevic
Editor
EMBO Molecular Medicine

***** Reviewer's comments *****

Referee #1 (Remarks for Author):

This manuscript describes the effects of DHCR24 inhibition on nonalcoholic fatty liver disease (NAFLD) and non-alcoholic steatohepatitis (NASH) in apoE*3 Leiden.CETP mice. Treatment with the novel DHCR24 inhibtitor SH42 increased the levels of desmosterol, an endogenous activator of LXR, and caused decreases of hepatic fat content and hepatic inflammation, notably Kupffer cell activation and monocyte immigration. In LXRalpha ko mice, these effects were not seen, indicating that the effects are LXR dependent. THese findings may lay the basis for the development of drug treatment of NAFLD and NASH. However, the study has at least two major limitations that question the conclusions of the authors:
1. Many effects are only trends but not statistically signficant or only at a $p < 0.05$ level. In this regard it is critical that the authors used parametric rather than non-parametric tests without proving normal frequency distribution. Already the visual inspection of the dots in the bar diagrams shows that in many instances the observations are not normally distributed. THere are many cases of wide or even clustered scatters. The depiction of variation as SEM's rather than SD's hides this problem. THis reviewer assumes that almost all findings will not remain statistically significant upon application of appropriate non-parametric statistical tests. On top of this, the authors did not adjust their explorative analyses for multiple testing. This should be done if several lipids or immune phenotypes are explored, also if the test results are distributed between several bar diagrams. FOr example figures 1G, H, I, J are four explorations that will need a Bonferroni-adjusted p-values of < 0.0125 for statistical significance (and this is rather permissive, because many other lipid species and classes were probably analysed as well but not reported. This problem holds true for other analyses as well.

2. As mentioned before, the diagrams show broad or clustered scatters data on lipid and immune phenotypes. This is most prominent in the untreated control condition. FOr example, several CTRL mice have as low liver fat content as SH42 treated

mice and appear to have no NAFLD. THis probably reflects the heterogeneous response of apoE3*Leiden.CETP mice to high fat diet. It was previosly described that upon fat feeding, some apoE3*Leiden.CETP mice respond with NAFLD whereas others do not. (PMID 29975550). It was therefore already suggested that NAFLD studies in this mouse model should pre-select responders (PMID: 29975550). Such a pre-selection may help to see more convincing and statistically significant differences between CTRL mice and SH42 treated mice also upon the use of the appropriate statistical tests. Moreover, They should do the intervention in responders only. This will lead to much clearer results either confirming or falsifying the hypothesis that DHCR24 inhibition improves or prevents NAFLD. Moreover, such a preselection of appropriate animals may also detect effects of DHCR24 inhibition on glucose tolerance and insulin resistance as well as circulting lipds.

Referee #2 (Comments on Novelty/Model System for Author):

The model system is well established and published

Referee #2 (Remarks for Author):

The authors studied whether the inhibition of DHRC24 through a synthetic compound (namely SH42) could be of use in the therapy of diet-induced NAFLD, in a well-established humanized mouse model for hyperlipidemia and atherosclerosis. They showed that treatment with SH42 ameliorates hepatic steatosis, thereby prevents Kupffer cell activation and immune cell infiltration and does not increase circulating lipids. Moreover, they provide evidence that SH42 treatment does not worsen hepatic steatosis.
This study provides first idea that SH42 might ameliorate hepatic inflammation, at least in mice. The mouse model is well chosen for this compound testing and the study well designed. However, the last conclusion that inhibition of DHCR24 delays high-fat diet induced liver inflammation and fibrosis is not really supported by the provided data. The writing of the manuscript could also be improved since many details are missing. It is an interesting study, but there are many open questions, which need to be improved:

1. The authors have previously published the SH42 inhibitor but have not proven that it directly inhibits DHCR24. Although the data presented here is interesting, it does not prove that SH42 directly binds and inhibits DHCR24. The authors should explore and discuss the possibility that some of their data can be explained by indirect effects.
2. The last conclusion that inhibition of DHCR24 delays high-fat diet induced liver inflammation and fibrosis is not really supported by the provided data, since it is only obvious that SH42 treatment reduces hepatic crown-like structures and a calculated tendency to reduced Sirius red staining. The authors would support their statement also by measuring plasma AST/ALT levels as marker for liver inflammation? Please provide here a more representative image (Figure 6H). Maybe the authors could reformulate their conclusion? In addition, other markers of fibrosis need to be tested/measured.
3. Upon SH42 treatment the authors saw a reduction in liver TAG, DAG and a tendency to reduced FFA and CE (Figure 1G-H). Can you speculate on the cause of this reduction in lipids, is it due to decreased lipid synthesis due to a lower availability of FA or increased lipolysis?
4. In the introduction the authors mention that desmosterol levels induce LXR -target genes while inhibiting SREBP target genes in macrophages, but is the same true for other cell types, e.g. hepatocytes and the liver? Could the increased desmosterol levels be of any harm for the animals, since they where highly increased? Why are the plasma desmosterol levels in LXR -deficient control mice detectable and in E3L.CETP control mice not (Figure 3A and S5E)? The expression levels of LXR -targets need to be determined.
5. The authors show that inhibition of DHCR24 reduces immune cell infiltration in the liver. Why did the authors show macrophage staining in 8 weeks treated mice and flow cytometry analysis in only 4 weeks treated mice?
6. Did the SH42 treatment alter the total amount of liver cells?
7. How did the mice cope with the SH42 treatment, especially long-term, how many of them died, was it well tolerated? In other words, what are the consequences of sky-high desmosterol levels on the physiology of the mice?
8. Differences in H&E staining might be better visible if the authors might include a higher magnification of the images, specifically in Figure 1B, 4A, and, 6B.
9. How and from which staining did you calculate the liver lipid area? Please specify in methods
10. In the introduction, the authors cite a study from 2016 for the global prevalence of NAFLD. There are newer studies available.
11. In the results section in paragraph 2 in the first sentence: I assume the authors meant "...scored as described detailed previously".
12. In the methods section: Please provide more information how the blood was centrifuged and how much plasma was used for the glucose and insulin measurement. Could the authors specify in the methods section why they used a modified steatosis score? For the lipidomics analysis, was always the same liver lobe collected? Please be more exact in the description of the sample preparation
13. In all Figures: the explanation for the abbreviation ctr is missing and for understanding it would be better to specify that you compared treatment vs. control for the significance test. In Figure 2A, 6E: please describe what the arrow indicates in the legend.

As a service to authors, EMBO provides authors with the possibility to transfer a manuscript that one journal cannot offer to publish to another EMBO publication. The full manuscript and if applicable, reviewers reports are automatically sent to the receiving journal to allow for fast handling and a prompt decision on your manuscript. For more details of this service, and to transfer your manuscript to another EMBO title please click on Link Not Available

Please do not share this URL as it will give anyone who clicks it access to your account.

We thank the reviewers for their valuable comments. Please find our point-by-point responses to the reviewers' comments below.

***** Reviewer's comments *****

Referee #1 (Remarks for Author):

This manuscript describes the effects of DHCR24 inhibition on nonalcoholic fatty liver disease (NAFLD) and non-alcoholic steatohepatitis (NASH) in ApoE*3 Leiden.CETP mice. Treatment with the novel DHCR24 inhibitor SH42 increased the levels of desmosterol, an endogenous activator of LXR, and caused decreases of hepatic fat content and hepatic inflammation, notably Kupffer cell activation and monocyte immigration. In LXRalpha ko mice, these effects were not seen, indicating that the effects are LXR dependent. These findings may lay the basis for the development of drug treatment of NAFLD and NASH.

However, the study has at least two major limitations that question the conclusions of the authors:

1. Many effects are only trends but not statistically significant or only at a $p < 0.05$ level. In this regard it is critical that the authors used parametric rather than non-parametric tests without proving normal frequency distribution. Already the visual inspection of the dots in the bar diagrams shows that in many instances the observations are not normally distributed. There are many cases of wide or even clustered scatters. The depiction of variation as SEM's rather than SD's hides this problem. This reviewer assumes that almost all findings will not remain statistically significant upon application of appropriate non-parametric statistical tests.

Re: We fully agree that all data should be checked for normal distribution to decide whether parametric or non-parametric tests have to be applied, and we sincerely apologize for this unintentional oversight. Indeed, some datasets did not pass a statistical test for Gaussian distribution, *e.g.* Figure 2B-K. Accordingly, a non-parametric Mann-Whitney test was now applied throughout our study**. Of note, changing to non-parametric statistical testing did not alter the main conclusions of this study**, as both hepatic steatosis including steatosis score (Fig 1C), lipid droplet positive area (Fig 1D), and liver inflammation including F4/80 positive area (Fig 2B and C), amount of total/resting Kupffer cells (Fig 2F and I), recruited monocytes (Fig 2J), and neutrophils (Fig 2K), remained significantly different (please find **new modified all figures** based on non-parametric tests in the revised manuscript).

On top of this, the authors did not adjust their explorative analyses for multiple testing. This should be done if several lipids or immune phenotypes are explored, also if the test results are distributed between several bar diagrams. For example figures 1G, H, I, J are four explorations that will need a Bonferroni-adjusted p-values of < 0.0125 for statistical significance (and this is rather permissive, because many other lipid species and classes were probably analysed as well but not reported). This problem holds true for other analyses as well.

Re: we agree with the referee that multiple testing correction is generally essential when multiple tests are performed. However, our intention was not to pinpoint, select and follow-up on specific lipid species, but rather show that all constituents of multiple lipid classes are showing changes in abundance upon SH42 treatment. Having that in mind and aiming at a visual representation, applying a correction (or not) does not affect the interpretation of the volcano plot. For example, in Figure 1F, all orange dots representing the TG class are moving to the left showing decrease in abundance. Important to reiterate here, we did not biologically follow-up on any specific lipid species. If we would have done so, we fully agree that multiple testing correction would have been mandatory. In turn, we refrained from multiple testing correction in the first instance. **Along this line of thought we have now removed all species names as well as the significance line for the p-value in the volcano plot (Figure 1F and Figure 4D).**

However, **for Figure 1G-J and Figure 4E-H we agree that multiple testing correction should be applied**. Nevertheless, instead of Bonferroni correction we opted for the generally accepted correction for this type of data that would be Benjamini-Hochberg correction (FDR). We have now accordingly modified the Result, Materials and Methods, and Figure legends section in the revised manuscript.

2. As mentioned before, the diagrams show broad or clustered scatters data on lipid and immune phenotypes. This is most prominent in the untreated control condition. For example, several CTRL mice have as low liver fat content as SH42 treated mice and appear to have no NAFLD. This probably reflects the heterogeneous response of apoE3*Leiden.CETP mice to high fat diet. It was previously described that upon fat feeding, some apoE3*Leiden.CETP mice respond with NAFLD whereas others do not. (PMID 29975550). It was therefore already suggested that NAFLD studies in this mouse model should pre-select responders (PMID: 29975550). Such a pre-selection may help to see more convincing and statistically significant differences between CTRL mice and SH42 treated mice also upon the use of the appropriate statistical tests. Moreover, They should do the intervention in responders only. This will lead to much clearer results either confirming or falsifying the hypothesis that DHCR24 inhibition improves or prevents NAFLD. Moreover, such a preselection of appropriate animals may also detect effects of DHCR24 inhibition on glucose tolerance and insulin resistance as well as circulating lipids.

Re: We fully agree with this suggestion and acknowledge that male APOE*3-Leiden.CETP mice display phenotypical heterogeneity in our setting. In fact, it is a routine procedure in our lab to exclude non-responders before any treatment. This is based on fasting plasma lipid levels, i.e., total cholesterol levels < 2 mM and triglyceride levels < 2 mM (i.e. exactly based on the criteria from the reference PMID 29975550). We apologize that we did not make this more clear in first instance and have now provided the detailed pre-selection step in the Materials and Methods section of the revised manuscript (lines 313-316).

Referee #2 (Comments on Novelty/Model System for Author):

The model system is well established and published

Referee #2 (Remarks for Author):

The authors studied whether the inhibition of DHRC24 through a synthetic compound (namely SH42) could be of use in the therapy of diet-induced NAFLD, in a well-established humanized mouse model for hyperlipidemia and atherosclerosis. They showed that treatment with SH42 ameliorates hepatic steatosis, thereby prevents Kupffer cell activation and immune cell infiltration and does not increase circulating lipids. Moreover, they provide evidence that SH42 treatment does not worsen hepatic steatosis.
This study provides first idea that SH42 might ameliorate hepatic inflammation, at least in mice. The mouse model is well chosen for this compound testing and the study well designed. However, the last conclusion that inhibition of DHCR24 delays high-fat diet induced liver inflammation and fibrosis is not really supported by the provided data. The writing of the manuscript could also be improved since many details are missing. It is an interesting study, but there are many open questions, which need to be improved:

1. The authors have previously published the SH42 inhibitor but have not proven that it directly inhibits DHCR24. Although the data presented here is interesting, it does not prove that SH42 directly binds and inhibits DHCR24. The authors should explore and discuss the possibility that some of their data can be explained by indirect effects.

Re: We thank the reviewer for the comment. Indeed, we were unfortunately not able to show the direct evidence that SH42 binds to DHCR24, which is mainly due to a lack of available pure enzyme and the fact that DHCR24 is membrane bound making its isolation and crystallization hardly possible. However, we have shown selectivity of SH42 within cholesterol biosynthesis (PMID: 28964935) using GC/MS analysis and a direct inhibition of DHCR24 is being deduced from desmosterol accumulation in vitro and in vivo (PMID: 28964935; PMID: 31548397; Figure 1A and 3A of this manuscript). In addition, our previous study also excluded the possible cross-reactivities of SH42 with other nuclear receptors, including farnesoid X receptor (FXR), pregnane X receptor (PXR), and retinoid X receptor (RXR) by monitoring the expression of their target genes (PMID 31548397). Despite of these, we acknowledge the possibility that some of our effects could be due to unknown 'off-target' effects of SH42 compound, and this has been now discussed in the revised manuscript (lines 299-303).

2. The last conclusion that inhibition of DHCR24 delays high-fat diet induced liver inflammation and fibrosis is not really supported by the provided data, since it is only obvious that SH42 treatment reduces hepatic crown-like structures and a calculated tendency to reduced Sirius red staining. The authors would support their statement also by measuring plasma AST/ALT levels as marker for liver inflammation? Please provide here a more representative image (Figure 6H). Maybe the authors could reformulate their conclusion? In addition, other markers of fibrosis need to be tested/measured.

Re: We thank the reviewer for this constructive comment, and will determine plasma ALT levels and liver hydroxyproline levels by ELISA, and mRNA expression of genes related to liver fibrosis by qPCR soon.

3. Upon SH42 treatment the authors saw a reduction in liver TAG, DAG and a tendency to

reduced FFA and CE (Figure 1G-H). Can you speculate on the cause of this reduction in lipids, is it due to decreased lipid synthesis due to a lower availability of FA or increased lipolysis?

Re: Reduction in liver lipids, which leads to significantly improved hepatic steatosis, could be a multi-facetted effect by SH42 treatment. On one hand, inhibition of DHCR24 by SH42 could directly reduce *de novo* cholesterol biosynthesis in both hepatocytes and non-parenchymal cells, as evidence by desmosterol accumulation in both liver and circulation (Fig 1A and Fig 3A). On the other hand, LXR activation by increased desmosterol in Kupffer cells suppresses Kupffer cell activation (Figure 2F-2I) thus reducing liver inflammation (Fig 2). Alleviation of liver inflammation has been shown to have multiple effects, including reduced fatty acid update, VLDL secretion, and lipid oxidation (PMID 25143667), which needs to be further investigated. We will add this discussion in the revised manuscript.

4. In the introduction the authors mention that desmosterol levels induce LXRα-target genes while inhibiting SREBP target genes in macrophages, but is the same true for other cell types, e.g. hepatocytes and the liver?

Re: Based on the convincing data we previously published (PMID 29632203), desmosterol and desmosterol mimetics confer selective regulation of LXR and SREBP in macrophages, but not in hepatocytes both in vitro and in mice. This is the reason why there is no difference in the gene expressions of LXR target genes by SH42 treatment in the whole liver tissue (please see details below).

Could the increased desmosterol levels be of any harm for the animals, since they were highly increased?

Re: We did not observe any signs of either general health issues, cataract or ichthyosis by SH42 treatment during both acute (PMID 31548397) and this chronic treatment.

Why are the plasma desmosterol levels in LXRα-deficient control mice detectable and in E3L.CETP control mice not (Figure 3A and S5E)?

Re: We apologize for this GC-MS artifact. This has been a multi-year project due to covid19, and on the way we replaced our old Bruker GC-MS with a new Agilent GC-MS. Due to the increased sensitivity of the new machine we could now also quantify desmosterol levels in control mice of the second experiment. We have clarified the details in the Supplementary materials and methods section (lines 33-34).

The expression levels of LXRα-targets need to be determined.

Re: We thank the reviewer for the comment. Please find the qPCR data in whole liver tissues below. In line with what we previously found (PMID 29632203), SH42 treatment did not influence the expression of LXR target genes in the whole liver, including *Abca1*, *Abcg1*, *Srebp1c*, and *Fas*.

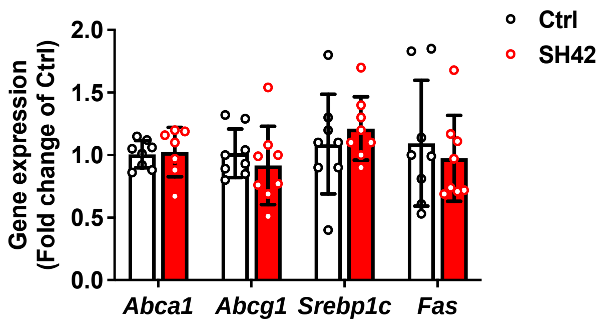

5. The authors show that inhibition of DHCR24 reduces immune cell infiltration in the liver. Why did the authors show macrophage staining in 8 weeks treated mice and flow cytometry analysis in only 4 weeks treated mice?

Re: We thank the reviewer for the comment. The experiment in LXR knockout mice was terminated after 4 weeks of high fat cholesterol diet (HFCD), as these mice already have very severe hepatic steatosis and inflammation (Figure 4 and 5). We wanted to compare the effects of SH42 treatment on immune cell infiltration in both WT and LXR knockout mice, so both flow cytometry analyses were done after 4 weeks of HFCD. There is no special reason why we used 8-weeks treated mice for F4/80 quantification. Of note, we observed a significant reduction of hepatic immune cells after both 4 and 8 weeks of SH42 treatment using either immunohistochemistry or flow cytometry.

6. Did the SH42 treatment alter the total amount of liver cells?

Re: Hepatic content of phospholipids (PL) between control and SH42 groups are equal (please see the details below). Given that PL are major membrane constituents that correlate with cell number, SH42 treatment likely did not alter the total amount of liver cells.

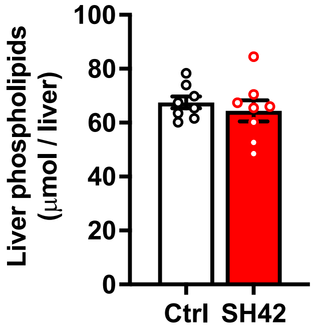

7. How did the mice cope with the SH42 treatment, especially long-term, how many of them died, was it well tolerated? In other words, what are the consequences of sky-high desmosterol levels on the physiology of the mice?

Re: We have routine veterinary examination of all treated mice in our animal facility. All animals were perfectly healthy without any signs of either general health issues, cataract or ichthyosis.

8. Differences in H&E staining might be better visible if the authors might include a higher magnification of the images, specifically in Figure 1B, 4A, and, 6B.

Re: We thank the reviewer for the suggestion and have now added a higher magnification of the images in Figures 1B, 4A, and 6B.

9. How and from which staining did you calculate the liver lipid area? Please specify in methods

Re: We used liver hematoxylin and eosin (HE) stained slides to visualize lipid droplets (i.e., areas that are not stained by HE), and used ImageJ software to select and quantify those unstained areas.
We apologize for the incomplete description and have now added the details in the methods section.

10. In the introduction, the authors cite a study from 2016 for the global prevalence of NAFLD. There are newer studies available.

Re: We thank the reviewer for the suggestion and have now updated that reference (PMID 35798021).

11. In the results section in paragraph 2 in the first sentence: I assume the authors meant "...scored as described detailed previously".

Re: We thank the reviewer for the comment. The sentence has now been corrected.

12. In the methods section: Please provide more information how the blood was centrifuged and how much plasma was used for the glucose and insulin measurement.

Re: We apologize for the incomplete description and have now added the details in the methods section.

Could the authors specify in the methods section why they used a modified steatosis score?

Re: We apologize for the description as we actually strictly followed the criteria proposed by Liang et al. (PMID 25535951) to score hepatic steatosis including microvesicular steatosis and macrovesicular steatosis, and hepatocellular hypertrophy (grade 0 - 3) based on the percentage of the total area affected. This has now been corrected in the methods section.

For the lipidomics analysis, was always the same liver lobe collected? Please be more exact in the description of the sample preparation.

Re: For all liver assays except for the flow cytometry, a small piece of the right liver lobe was used. The remainder of the right lobe and whole left lobes were used to isolate enough immune cells for the flow cytometry analysis. These has been now clarified in the methods section.

13. In all Figures: the explanation for the abbreviation ctrl is missing and for understanding it would be better to specify that you compared treatment vs. control for the significance test. In Figure 2A, 6E: please describe what the arrow indicates in the legend.

Re: We thank the reviewer for the suggestion and have now clearly explained the abbreviations ‘Ctrl’ and ‘SH42’, we clarified the comparison (SH42/Ctrl), and we clarified that arrows indicate hepatic crown-like structures in figure legends.

7th Dec 2022

Dear Dr. Wang,

Thank you for your response to the editorial decision on your manuscript entitled "Inhibition of DHCR24 activates LXRα to ameliorate hepatic steatosis and inflammation". I have now carefully examined the arguments provided in your letter and discussed them with the other members of our editorial team. As I mentioned in the previous decision letter, we would be willing to reconsider your manuscript if you provide data that would considerably strengthen the message of the study and address the referees concerns in full. From your point-by-point response it seems that you can address all the referees concerns, so please submit the revised version of the manuscript within next three months. Please let us know if you require longer to complete the revision.

Further consideration of the revised article will entail a second round of review and we cannot guarantee the outcome of the reevaluation; therefore, I would strongly advise against returning an incomplete revision.

I look forward to receiving your revised manuscript.

Yours sincerely,

Zeljko Durdevic

Zeljko Durdevic
Editor
EMBO Molecular Medicine

*****

When submitting your revised manuscript, please carefully review the instructions that follow below. We perform an initial quality control of all revised manuscripts before re-review; failure to include requested items will delay the evaluation of your revision.

We require:

1) A .docx formatted version of the manuscript text (including legends for main figures, EV figures and tables). Please make sure that the changes are highlighted to be clearly visible.

2) Individual production quality figure files as .eps, .tif, .jpg (one file per figure). For guidance, download the 'Figure Guide PDF': (https://www.embopress.org/page/journal/17574684/authorguide#figureformat).

3) A .docx formatted letter INCLUDING the reviewers' reports and your detailed point-by-point responses to their comments. As part of the EMBO Press transparent editorial process, the point-by-point response is part of the Review Process File (RPF), which will be published alongside your paper.

4) A complete author checklist, which you can download from our author guidelines (https://www.embopress.org/page/journal/17574684/authorguide#submissionofrevisions). Please insert information in the checklist that is also reflected in the manuscript. The completed author checklist will also be part of the RPF.

5) Please note that all corresponding authors are required to supply an ORCID ID for their name upon submission of a revised manuscript.

6) It is mandatory to include a 'Data Availability' section after the Materials and Methods. Before submitting your revision, primary datasets produced in this study need to be deposited in an appropriate public database, and the accession numbers and database listed under 'Data Availability'. Please remember to provide a reviewer password if the datasets are not yet public (see https://www.embopress.org/page/journal/17574684/authorguide#dataavailability).

In case you have no data that requires deposition in a public database, please state so in this section. Note that the Data Availability Section is restricted to new primary data that are part of this study.

7) For data quantification: please specify the name of the statistical test used to generate error bars and P values, the number (n) of independent experiments (specify technical or biological replicates) underlying each data point and the test used to calculate p-values in each figure legend. The figure legends should contain a basic description of n, P and the test applied. Graphs must include a description of the bars and the error bars (s.d., s.e.m.). See also 'Figure Legend' guidelines: https://www.embopress.org/page/journal/17574684/authorguide#figureformat

8) At EMBO Press we ask authors to provide source data for the main manuscript figures. Our source data coordinator will contact you to discuss which figure panels we would need source data for and will also provide you with helpful tips on how to upload and organize the files.

9) Our journal encourages inclusion of *data citations in the reference list* to directly cite datasets that were re-used and obtained from public databases. Data citations in the article text are distinct from normal bibliographical citations and should directly link to the database records from which the data can be accessed. In the main text, data citations are formatted as follows: "Data ref: Smith et al, 2001" or "Data ref: NCBI Sequence Read Archive PRJNA342805, 2017". In the Reference list, data citations must be labeled with "[DATASET]". A data reference must provide the database name, accession number/identifiers and a resolvable link to the landing page from which the data can be accessed at the end of the reference. Further instructions are available at .

10) We replaced Supplementary Information with Expanded View (EV) Figures and Tables that are collapsible/expandable online. A maximum of 5 EV Figures can be typeset. EV Figures should be cited as 'Figure EV1, Figure EV2" etc... in the text and their respective legends should be included in the main text after the legends of regular figures.

- For the figures that you do NOT wish to display as Expanded View figures, they should be bundled together with their legends in a single PDF file called *Appendix*, which should start with a short Table of Content. Appendix figures should be referred to in the main text as: "Appendix Figure S1, Appendix Figure S2" etc.

- Additional Tables/Datasets should be labeled and referred to as Table EV1, Dataset EV1, etc. Legends have to be provided in a separate tab in case of .xls files. Alternatively, the legend can be supplied as a separate text file (README) and zipped together with the Table/Dataset file.

See detailed instructions here:

.

11) The paper explained: EMBO Molecular Medicine articles are accompanied by a summary of the articles to emphasize the major findings in the paper and their medical implications for the non-specialist reader. Please provide a draft summary of your article highlighting

- the medical issue you are addressing,

- the results obtained and

- their clinical impact.

This may be edited to ensure that readers understand the significance and context of the research. Please refer to any of our published articles for an example.

12) For more information: There is space at the end of each article to list relevant web links for further consultation by our readers. Could you identify some relevant ones and provide such information as well? Some examples are patient associations, relevant databases, OMIM/proteins/genes links, author's websites, etc...

13) Author contributions: You will be asked to provide CRediT (Contributor Role Taxonomy) terms in the submission system. These replace a narrative author contribution section in the manuscript.

14) A Conflict of Interest statement should be provided in the main text.

15) Every published paper now includes a 'Synopsis' to further enhance discoverability. Synopses are displayed on the journal webpage and are freely accessible to all readers. They include a short stand first (maximum of 300 characters, including space) as well as 2-5 one-sentences bullet points that summarizes the paper. Please write the bullet points to summarize the key NEW findings. They should be designed to be complementary to the abstract - i.e. not repeat the same text. We encourage inclusion

of key acronyms and quantitative information (maximum of 30 words / bullet point). Please use the passive voice. Please attach these in a separate file or send them by email, we will incorporate them accordingly.

Please also suggest a striking image or visual abstract to illustrate your article as a PNG file 550 px wide x 300-600 px high.

EMBO Molecular Medicine has a "scooping protection" policy, whereby similar findings that are published by others during review or revision are not a criterion for rejection. Should you decide to submit a revised version, I do ask that you get in touch after three months if you have not completed it, to update us on the status.

Please note: When submitting your revision you will be prompted to enter your funding and payment information. This will allow Wiley to send you a quote for the article processing charge (APC) in case of acceptance. This quote takes into account any reduction or fee waivers that you may be eligible for. Authors do not need to pay any fees before their manuscript is accepted and transferred to the publisher.

EMBO Press participates in many Publish and Read agreements that allow authors to publish Open Access with reduced/no publication charges. Check your eligibility: https://authorservices.wiley.com/author-resources/Journal-Authors/open-access/affiliation-policies-payments/index.html

We thank the reviewers for their valuable comments. Please find our point-by-point responses to the reviewers' comments below.

***** Reviewer's comments *****

Referee #1 (Remarks for Author):

This manuscript describes the effects of DHCR24 inhibition on nonalcoholic fatty liver disease (NAFLD) and non-alcoholic steatohepatitis (NASH) in ApoE*3 Leiden.CETP mice. Treatment with the novel DHCR24 inhibitor SH42 increased the levels of desmosterol, an endogenous activator of LXR, and caused decreases of hepatic fat content and hepatic inflammation, notably Kupffer cell activation and monocyte immigration. In LXRalpha ko mice, these effects were not seen, indicating that the effects are LXR dependent. These findings may lay the basis for the development of drug treatment of NAFLD and NASH.

However, the study has at least two major limitations that question the conclusions of the authors:

1. Many effects are only trends but not statistically significant or only at a $p < 0.05$ level. In this regard it is critical that the authors used parametric rather than non-parametric tests without proving normal frequency distribution. Already the visual inspection of the dots in the bar diagrams shows that in many instances the observations are not normally distributed. There are many cases of wide or even clustered scatters. The depiction of variation as SEM's rather than SD's hides this problem. This reviewer assumes that almost all findings will not remain statistically significant upon application of appropriate non-parametric statistical tests.

Re: We fully agree that all data should be checked for normal distribution to decide whether parametric or non-parametric tests have to be applied, and we sincerely apologize for this unintentional oversight. Indeed, some datasets did not pass a statistical test for Gaussian distribution, *e.g.* Figure 2B-K. Accordingly, a non-parametric Mann-Whitney test was now applied throughout our study. Of note, changing to non-parametric statistical testing did not alter the main conclusions of this study, as both hepatic steatosis including steatosis score (New Fig 1C), lipid droplet positive area (New Fig 1D), and liver inflammation including F4/80 positive area (New Fig 2B and C), amount of total and resting Kupffer cells (New Fig 2F and I), recruited monocytes (New Fig 2J), and neutrophils (New Fig 2K), remained significantly different (please find new modified figures based on non-parametric tests in the revised manuscript).

On top of this, the authors did not adjust their explorative analyses for multiple testing. This should be done if several lipids or immune phenotypes are explored, also if the test results are distributed between several bar diagrams. For example figures 1G, H, I, J are four explorations that will need a Bonferroni-adjusted p-values of < 0.0125 for statistical significance (and this is rather permissive, because many other lipid species and classes were probably analysed as well but not reported). This problem holds true for other analyses as well.

Re: We agree with the referee that multiple testing correction is generally essential when multiple tests are performed. However, our intention was not to pinpoint, select and follow-up on

specific lipid species, but rather show that all constituents of multiple lipid classes are showing changes in abundance upon SH42 treatment. Having that in mind and aiming at a visual representation, applying a correction (or not) does not affect the interpretation of the volcano plot. For example, in Figure 1F, all orange dots representing the TG class are moving to the left showing decrease in abundance. Important to reiterate here, we did not biologically follow-up on any specific lipid species. If we would have done so, we fully agree that multiple testing correction would have been mandatory. In turn, we refrained from multiple testing correction in the first instance. Along this line of thought we have now removed all species names as well as the significance line for the p-value in the volcano plot (New Figure 1F and New Figure 4D).

However, for Figure 1G-J and Figure 4E-H we agree that multiple testing correction should be applied. Nevertheless, instead of Bonferroni correction we opted for the generally accepted correction for this type of data that would be Benjamini-Hochberg correction (FDR). We have now accordingly modified the Results (lines 143 - 149), Materials and Methods (lines 399 - 401), and Figure Legends section in the revised manuscript.

2. As mentioned before, the diagrams show broad or clustered scatters data on lipid and immune phenotypes. This is most prominent in the untreated control condition. For example, several CTRL mice have as low liver fat content as SH42 treated mice and appear to have no NAFLD. This probably reflects the heterogeneous response of apoE3*Leiden.CETP mice to high fat diet. It was previously described that upon fat feeding, some apoE3*Leiden.CETP mice respond with NAFLD whereas others do not. (PMID 29975550). It was therefore already suggested that NAFLD studies in this mouse model should pre-select responders (PMID: 29975550). Such a pre-selection may help to see more convincing and statistically significant differences between CTRL mice and SH42 treated mice also upon the use of the appropriate statistical tests. Moreover, They should do the intervention in responders only. This will lead to much clearer results either confirming or falsifying the hypothesis that DHCR24 inhibition improves or prevents NAFLD. Moreover, such a preselection of appropriate animals may also detect effects of DHCR24 inhibition on glucose tolerance and insulin resistance as well as circulating lipids.

Re: We fully agree with this suggestion and acknowledge that male APOE*3-Leiden.CETP mice display phenotypical heterogeneity in our setting. In fact, it is a routine procedure in our lab to exclude non-responders before any treatment, and was also done for the experiments described in this paper. Exclusion of mice is based on fasting plasma lipid levels, i.e., total cholesterol levels < 2 mM and triglyceride levels < 2 mM (i.e. based on the exact criteria from the reference PMID 29975550). We apologize that we did not make this clear in first instance and have now provided the detailed pre-selection step in the Materials and Methods section (lines 347 - 350).

Referee #2 (Comments on Novelty/Model System for Author):

The model system is well established and published

Referee #2 (Remarks for Author):

The authors studied whether the inhibition of DHRC24 through a synthetic compound (namely

SH42) could be of use in the therapy of diet-induced NAFLD, in a well-established humanized mouse model for hyperlipidemia and atherosclerosis. They showed that treatment with SH42 ameliorates hepatic steatosis, thereby prevents Kupffer cell activation and immune cell infiltration and does not increase circulating lipids. Moreover, they provide evidence that SH42 treatment does not worsen hepatic steatosis.

This study provides first idea that SH42 might ameliorate hepatic inflammation, at least in mice. The mouse model is well chosen for this compound testing and the study well designed. However, the last conclusion that inhibition of DHCR24 delays high-fat diet induced liver inflammation and fibrosis is not really supported by the provided data. The writing of the manuscript could also be improved since many details are missing. It is an interesting study, but there are many open questions, which need to be improved:

1. The authors have previously published the SH42 inhibitor but have not proven that it directly inhibits DHCR24. Although the data presented here is interesting, it does not prove that SH42 directly binds and inhibits DHCR24. The authors should explore and discuss the possibility that some of their data can be explained by indirect effects.

Re: We thank the reviewer for the comment. Indeed, we were unfortunately not able to show the direct evidence that SH42 binds to DHCR24, which is mainly due to a lack of available pure enzyme and the fact that DHCR24 is membrane bound making its isolation and crystallization hardly possible. However, we have shown selectivity of SH42 within cholesterol biosynthesis (PMID: 28964935) using GC/MS analysis and a direct inhibition of DHCR24 is being deduced from desmosterol accumulation in vitro and in vivo (PMID: 28964935; PMID: 31548397; New Figure 1A and 3A). In addition, our previous study also excluded the possible cross-reactivities of SH42 with other nuclear receptors, including farnesoid X receptor (FXR), pregnane X receptor (PXR), and retinoid X receptor (RXR) by monitoring their target gene expressions (PMID 31548397). Despite of these, we acknowledge the possibility that some of our effects could be due to unknown effects of SH42 compound, and this has been now added to the Discussion section of the revised manuscript (lines 333 -338).

2. The last conclusion that inhibition of DHCR24 delays high-fat diet induced liver inflammation and fibrosis is not really supported by the provided data, since it is only obvious that SH42 treatment reduces hepatic crown-like structures and a calculated tendency to reduced Sirius red staining. The authors would support their statement also by measuring plasma AST/ALT levels as marker for liver inflammation? Please provide here a more representative image (Figure 6H). Maybe the authors could reformulate their conclusion? In addition, other markers of fibrosis need to be tested/measured.

Re: We thank the reviewer for this constructive comment, and have now measured plasma ALT levels. Consistently with the reduced hepatic crown-like structures, we found a significant reduction in plasma ALT levels in SH42-treated mice (-42%, $p < 0.05$; New Fig. 6J).

To better quantify liver fibrosis, we have re-stained liver collagen with Sirius Red/Fast Green staining and observed a robust and significant reduction of liver collagen content in SH42-treated

mice (-50%, p = 0.0055, New Fig. 6H and 6I). New representative images of liver Sirius Red/Fast Green staining have now been presented (New Fig. 6H). We also have now reformulated our conclusion accordingly (lines 223 – 224; 230 – 236).

3. Upon SH42 treatment the authors saw a reduction in liver TAG, DAG and a tendency to reduced FFA and CE (Figure 1G-H). Can you speculate on the cause of this reduction in lipids, is it due to decreased lipid synthesis due to a lower availability of FA or increased lipolysis?

Re: Reduction in liver lipids, which leads to significantly improved hepatic steatosis, could be a multi-facetted effect by SH42 treatment. On one hand, inhibition of DHCR24 by SH42 could directly reduce *de novo* cholesterol biosynthesis in both hepatocytes and non-parenchymal cells, as evidenced by desmosterol accumulation in both liver and circulation (New Fig 1A and New Fig 3A). On the other hand, SH42 treatment reduces liver inflammation (New Fig 2). Alleviation of liver inflammation has been shown to have multiple effects, including reduced fatty acid update, VLDL secretion, and lipid oxidation (PMID 25143667), which could be the cause of reduction in liver lipids by SH42 treatment. We have now added this to the Discussion section of the revised manuscript (lines 293 - 296).

4. In the introduction the authors mention that desmosterol levels induce LXRα-target genes while inhibiting SREBP target genes in macrophages, but is the same true for other cell types, e.g. hepatocytes and the liver?

Re: Based on the convincing data we previously published (PMID 29632203), desmosterol and desmosterol mimetics confer selective regulation of LXR and SREBP in macrophages, but not in hepatocytes both in vitro and in mice. This is the reason why there is no difference in the gene expression of LXR target genes by SH42 treatment in the whole liver tissue, in which hepatocytes are dominantly present (please see details below).

Could the increased desmosterol levels be of any harm for the animals, since they were highly increased?

Re: We did not observe any signs of either general health issues, cataract or ichthyosis by SH42 treatment during both acute (PMID 31548397) and this chronic treatment.

Why are the plasma desmosterol levels in LXRα-deficient control mice detectable and in E3L.CETP control mice not (Figure 3A and S5E)?

Re: We apologize for this GC-MS artifact. This has been a multi-year project due to covid19, and on the way we replaced our old Bruker GC-MS with a new Agilent GC-MS. Due to the increased sensitivity of the new machine we could now also quantify desmosterol levels in control mice. We have clarified the details in the Appendix Materials and Method section (line 66 - 67).

The expression levels of LXRα-targets need to be determined.

Re: We thank the reviewer for the comment. Please find the qPCR data in whole liver tissues below. In line with what we previously found (PMID 29632203), SH42 treatment did not influence LXR target gene expressions in the whole liver, including *Abca1*, *Abcg1*, *Srebp1c*, and *Fas*. This is in line with the fact that the dominant cell type in the liver are hepatocytes, in which desmosterol does not regulate LXR (see response to comment 4).

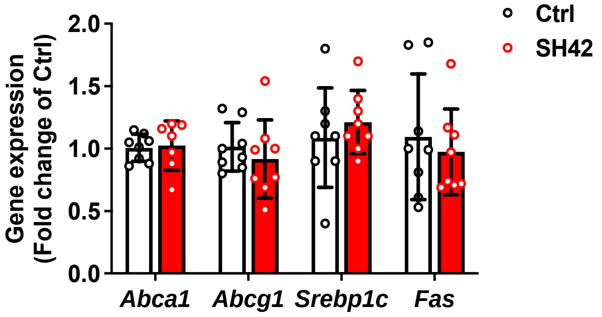

5. The authors show that inhibition of DHCR24 reduces immune cell infiltration in the liver. Why did the authors show macrophage staining in 8 weeks treated mice and flow cytometry analysis in only 4 weeks treated mice?

Re: We thank the reviewer for the comment. The experiment in LXR knockout mice was terminated after 4 weeks of high fat cholesterol diet treatment, as these mice already have very severe hepatic steatosis and inflammation (New Figures 4 and 5). We wanted to compare the effects of SH42 treatment on immune cell infiltration in both WT and LXR knockout mice, so both flow cytometry analyses were done after 4 weeks of high fat cholesterol diet treatment. There is no special reason why we used 8-weeks treated mice for F4/80 quantification. Of note, we observed a significant reduction of hepatic immune cells after both 4 and 8 weeks of SH42 treatment using either immunohistochemistry or flow cytometry.

6. Did the SH42 treatment alter the total amount of liver cells?

Re: Hepatic content of phospholipids (PL) between control and SH42 groups are equal (please see the details below). Given that PL are major membrane constituents that correlate with cell number, SH42 treatment likely did not alter the total amount of liver cells.

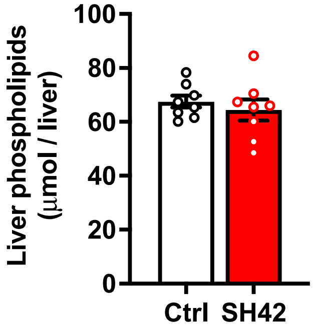

7. How did the mice cope with the SH42 treatment, especially long-term, how many of them

died, was it well tolerated? In other words, what are the consequences of sky-high desmosterol levels on the physiology of the mice?

Re: We have routine veterinary examination of all treated mice in our animal facility. All animals were perfectly healthy without any signs of either general health issues, cataract or ichthyosis, and no animals died.

8. Differences in H&E staining might be better visible if the authors might include a higher magnification of the images, specifically in Figure 1B, 4A, and, 6B.

Re: We thank the reviewer for the suggestion and have now added a higher magnification of the images in New Figures 1B, 4A, and 6B.

9. How and from which staining did you calculate the liver lipid area? Please specify in methods

Re: We used paraffin-embedded liver hematoxylin and eosin (HE) stained slides to visualize lipid droplets (i.e., areas that are not stained by HE), and used ImageJ software to select and quantify those unstained areas. We apologize for the incomplete description and have now added the details in the Appendix Materials and Methods section (lines 52 - 54).

10. In the introduction, the authors cite a study from 2016 for the global prevalence of NAFLD. There are newer studies available.

Re: We thank the reviewer for the suggestion and have now updated that reference (Riazi et al, 2022; PMID 35798021) in the reviser manuscript (line 59).

11. In the results section in paragraph 2 in the first sentence: I assume the authors meant "...scored as described detailed previously".

Re: We thank the reviewer for the comment. The sentence has now been corrected in the revised manuscript (line 134).

12. In the methods section: Please provide more information how the blood was centrifuged and how much plasma was used for the glucose and insulin measurement.

Re: We apologize for the incomplete description and have now added the details in the Appendix Materials and Methods section (lines 29 -31).

Could the authors specify in the methods section why they used a modified steatosis score?

Re: We apologize for the description as we actually strictly followed the criteria proposed by Liang et al. (PMID 25535951) to score hepatic steatosis including microvesicular steatosis and macrovesicular steatosis, and hepatocellular hypertrophy (grade 0 - 3) based on the percentage of the total area affected. This has now been corrected in the Appendix Materials and Methods section (lines 49).

For the lipidomics analysis, was always the same liver lobe collected? Please be more exact in the description of the sample preparation.

Re: For all liver assays except for the flow cytometry, a small piece of the right liver lobe was used. The remainder of the right lobe and whole left lobes were used to isolate sufficient immune cells for the flow cytometry analysis. These has been now clarified in the Appendix Materials and Methods section (lines 99 - 101).

13. In all Figures: the explanation for the abbreviation ctrl is missing and for understanding it would be better to specify that you compared treatment vs. control for the significance test. In Figure 2A, 6E: please describe what the arrow indicates in the legend.

Re: We thank the reviewer for the suggestion and have now clearly explained the abbreviations ‘Ctrl’ and ‘SH42’, we clarified the comparison (SH42/Ctrl), and we clarified that arrows indicate hepatic crown-like structures in figure legends.

22nd Mar 2023

Dear Dr. Wang,

Thank you for the submission of your revised manuscript to EMBO Molecular Medicine. We have now heard back from the two referees who we asked to re-evaluate your manuscript. In addition, I have also sought external advice on the study from an expert in the field. As you will see from the reports below, while the referee #1 supports publication of your manuscript, referee #2 raises a number of concerns particularly regarding conclusions not supported by the data. Our external advisor acknowledged interest of the study and overall conclusiveness of the data presented, however, in his/her opinion concerns of the referee #2 are valid and should be addressed in additional and final round of revision. The first two points of the referee #2 should be addressed by removing the misleading statements and adjusting conclusions that should be supported by the data. The last point should be addressed experimentally by performing oil red O staining.

Further consideration of a revision that addresses reviewer's concerns in full will entail an additional round of review. Acceptance or rejection of the manuscript will depend on the completeness of your responses included in the next, final version of the manuscript. For this reason, and to save you from any frustrations in the end, I would strongly advise against returning an incomplete revision.

We would welcome the submission of a revised version within three months for further consideration. Please let us know if you require longer to complete the revision.

I look forward to receiving your revised manuscript.

Yours sincerely,

Zeljko Durdevic

Zeljko Durdevic
Editor
EMBO Molecular Medicine

*****

When submitting your revised manuscript, please carefully review the instructions that follow below. We perform an initial quality control of all revised manuscripts before re-review; failure to include requested items will delay the evaluation of your revision.

We require:

1) A .docx formatted version of the manuscript text (including legends for main figures, EV figures and tables). Please make sure that the changes are highlighted to be clearly visible.

2) Individual production quality figure files as .eps, .tif, .jpg (one file per figure). For guidance, download the 'Figure Guide PDF': (https://www.embopress.org/page/journal/17574684/authorguide#figureformat).

3) A .docx formatted letter INCLUDING the reviewers' reports and your detailed point-by-point responses to their comments. As part of the EMBO Press transparent editorial process, the point-by-point response is part of the Review Process File (RPF), which will be published alongside your paper.

4) A complete author checklist, which you can download from our author guidelines (https://www.embopress.org/page/journal/17574684/authorguide#submissionofrevisions). Please insert information in the checklist that is also reflected in the manuscript. The completed author checklist will also be part of the RPF.

5) Please note that all corresponding authors are required to supply an ORCID ID for their name upon submission of a revised

manuscript.

6) It is mandatory to include a 'Data Availability' section after the Materials and Methods. Before submitting your revision, primary datasets produced in this study need to be deposited in an appropriate public database, and the accession numbers and database listed under 'Data Availability'. Please remember to provide a reviewer password if the datasets are not yet public (see https://www.embopress.org/page/journal/17574684/authorguide#dataavailability).

In case you have no data that requires deposition in a public database, please state so in this section. Note that the Data Availability Section is restricted to new primary data that are part of this study.

7) For data quantification: please specify the name of the statistical test used to generate error bars and P values, the number (n) of independent experiments (specify technical or biological replicates) underlying each data point and the test used to calculate p-values in each figure legend. The figure legends should contain a basic description of n, P and the test applied. Graphs must include a description of the bars and the error bars (s.d., s.e.m.). See also 'Figure Legend' guidelines: https://www.embopress.org/page/journal/17574684/authorguide#figureformat

8) At EMBO Press we ask authors to provide source data for the main manuscript figures. Our source data coordinator will contact you to discuss which figure panels we would need source data for and will also provide you with helpful tips on how to upload and organize the files.

9) Our journal encourages inclusion of *data citations in the reference list* to directly cite datasets that were re-used and obtained from public databases. Data citations in the article text are distinct from normal bibliographical citations and should directly link to the database records from which the data can be accessed. In the main text, data citations are formatted as follows: "Data ref: Smith et al, 2001" or "Data ref: NCBI Sequence Read Archive PRJNA342805, 2017". In the Reference list, data citations must be labeled with "[DATASET]". A data reference must provide the database name, accession number/identifiers and a resolvable link to the landing page from which the data can be accessed at the end of the reference. Further instructions are available at .

10) We replaced Supplementary Information with Expanded View (EV) Figures and Tables that are collapsible/expandable online. A maximum of 5 EV Figures can be typeset. EV Figures should be cited as 'Figure EV1, Figure EV2" etc... in the text and their respective legends should be included in the main text after the legends of regular figures.

- For the figures that you do NOT wish to display as Expanded View figures, they should be bundled together with their legends in a single PDF file called *Appendix*, which should start with a short Table of Content. Appendix figures should be referred to in the main text as: "Appendix Figure S1, Appendix Figure S2" etc.

- Additional Tables/Datasets should be labeled and referred to as Table EV1, Dataset EV1, etc. Legends have to be provided in a separate tab in case of .xls files. Alternatively, the legend can be supplied as a separate text file (README) and zipped together with the Table/Dataset file.

See detailed instructions here:

.

11) The paper explained: EMBO Molecular Medicine articles are accompanied by a summary of the articles to emphasize the major findings in the paper and their medical implications for the non-specialist reader. Please provide a draft summary of your article highlighting

- the medical issue you are addressing,

- the results obtained and

- their clinical impact.

This may be edited to ensure that readers understand the significance and context of the research. Please refer to any of our published articles for an example.

12) For more information: There is space at the end of each article to list relevant web links for further consultation by our readers. Could you identify some relevant ones and provide such information as well? Some examples are patient associations, relevant databases, OMIM/proteins/genes links, author's websites, etc...

13) Author contributions: You will be asked to provide CRediT (Contributor Role Taxonomy) terms in the submission system. These replace a narrative author contribution section in the manuscript.

14) A Conflict of Interest statement should be provided in the main text.

15) Every published paper now includes a 'Synopsis' to further enhance discoverability. Synopses are displayed on the journal webpage and are freely accessible to all readers. They include a short stand first (maximum of 300 characters, including space) as well as 2-5 one-sentences bullet points that summarizes the paper. Please write the bullet points to summarize the key NEW findings. They should be designed to be complementary to the abstract - i.e. not repeat the same text. We encourage inclusion of key acronyms and quantitative information (maximum of 30 words / bullet point). Please use the passive voice. Please attach these in a separate file or send them by email, we will incorporate them accordingly.

Please also suggest a striking image or visual abstract to illustrate your article as a PNG file 550 px wide x 300-600 px high.

EMBO Molecular Medicine has a "scooping protection" policy, whereby similar findings that are published by others during review or revision are not a criterion for rejection. Should you decide to submit a revised version, I do ask that you get in touch after three months if you have not completed it, to update us on the status.

Please note: When submitting your revision you will be prompted to enter your funding and payment information. This will allow Wiley to send you a quote for the article processing charge (APC) in case of acceptance. This quote takes into account any reduction or fee waivers that you may be eligible for. Authors do not need to pay any fees before their manuscript is accepted and transferred to the publisher.

EMBO Press participates in many Publish and Read agreements that allow authors to publish Open Access with reduced/no publication charges. Check your eligibility: https://authorservices.wiley.com/author-resources/Journal-Authors/open-access/affiliation-policies-payments/index.html

***** Reviewer's comments *****

Referee #1 (Remarks for Author):

The authors adressed my previous criticisms on statistics

Referee #2 (Comments on Novelty/Model System for Author):

see the authors response:
Re: We fully agree with this suggestion and acknowledge that male APOE*3-Leiden.CETP mice display phenotypical heterogeneity in our setting. In fact, it is a routine procedure in our lab to exclude non-responders before any treatment, and was also done for the experiments described in this paper. Exclusion of mice is based on fasting plasma lipid levels, i.e., total cholesterol levels < 2 mM and triglyceride levels < 2 mM (i.e. based on the exact criteria from the reference PMID 29975550).

Referee #2 (Remarks for Author):

The authors have made only minimal changes. When the figures are compared only Figure 6J was added and Figure 6H replaced.
There are still multiple issues that need to be corrected:
1. Line 335/336; "...and thereby proved selectivity (of SH42) for DHCR24 (Korner et al., 2019". This statement is misleading since the authors have not shown at all the SH42 binds to DHCR42. Unless they can show direct binding of SH42 to DHCR42, they will have to remove all statements that are misleading.
2. The last conclusion that inhibition of DHCR24 delays HFD-induced liver inflammation, fibrosis and injury is still not supported by the provided data and needs to be rewritten. The authors don't actually know if it is really delayed or if the mice are protected by the treatment. The authors have two choices: either remove statements that are not supported by the data, or check for many fibrosis markers, as suggested already previously - e.g., on gene expression level.
3. The response to 9 is not satisfying "Re: We used paraffin-embedded liver hematoxylin and eosin (HE) stained slides to visualize lipid droplets (i.e., areas that are not stained by HE), and used ImageJ software to select and quantify those unstained areas." This is not the correct way to do this experiment. The author need to stain with Oil Red O or a similar stain that is specific for lipids.

Zhou *et al*. Point-by-point Response

Dear Dr. Durdevic,

We would like to express our appreciation for your evaluating our manuscript entitled 'Inhibition of DHCR24 activates LXRα to ameliorate hepatic steatosis and inflammation' (EMM-2022-16845-V3) and for granting us the opportunity to resubmit it. I would like to extend my gratitude to referee #1 for promptly endorsing the publication of our manuscript, and to referee #2 for providing additional comments.

Based on your and referee #2's constructive feedback, we have performed Oil-Red O staining in liver samples from each animal studies, and re-quantified liver lipid positive areas accordingly. In addition, concerning the Referee #2's comment on the inhibition of DHCR24 by SH42, we would like to point out the following adaptations.

1. We have removed any mention of a "selective" inhibition.
2. We added a paragraph pointing out that we do not know if there are other targets or indirect effects on DHCR24.

It is important to point out that indeed we do not have co-crystallization data, but the previously described observation of accumulation of desmosterol from both in vitro and in vivo studies clearly pinpoints to pharmacological inhibition of DHCR24 by SH42, that may be direct or indirect. We hope we could clarify this issue in the revised manuscript.

Please find our point-by-point rebuttal letter for our response to the reviewer' comments. All authors have approved the final version of the revised manuscript, and the authors have no conflicting financial interests.

Yanan Wang

***** Reviewer's comments *****

Referee #1 (Remarks for Author):

The authors adressed my previous criticisms on statistics

Referee #2 (Remarks for Author):

The authors have made only minimal changes. When the figures are compared only Figure 6J was added and Figure 6H replaced.
There are still multiple issues that need to be corrected:
1. Line 335/336; "...and thereby proved selectivity (of SH42) for DHCR24 (Korner et al., 2019". This statement is misleading since the authors have not shown at all the SH42 binds to DHCR42. Unless they can show direct binding of SH42 to DHCR42, they will have to remove all statements that are misleading.

Re: We apologize for our misleading statement and have now removed any misleading descriptions. Additionally, we have addressed this limitation in the Discussion section to ensure clarity for the readers.

Specifically:

1. We have removed any mention of a “selective” inhibition.
2. We added a paragraph pointing out that we do not know if there are other targets or indirect effects on DHCR24 into the Discussion section.

   “*Of note, our study is limited by our inability to provide direct evidence of SH42 binding to DHCR24, due to a lack of available pure enzyme and the fact that DHCR24 is membrane bound making its isolation and co-crystallization very difficult. Therefore, we cannot exclude that SH42 exerts indirect inhibitory actions responsible for desmosterol accumulation or interaction with yet undiscovered targets contributing to the improvement of the NAFLD phenotype in our study*.” (lines 334-342)

2. The last conclusion that inhibition of DHCR24 delays HFD-induced liver inflammation, fibrosis and injury is still not supported by the provided data and needs to be rewritten. The authors don't actually know if it is really delayed or if the mice are protected by the treatment. The authors have two choices: either remove statements that are not supported by the data, or check for many fibrosis markers, as suggested already previously - e.g., on gene expression level.
Re: We apologize for the overstatements which have now been corrected in the Result section, and the Discussion section
Specifically:

1. We have removed all relevant statements that "Finally, we also showed that SH42 treatment delays NAFLD progression from simple steatosis to NASH." in the Abstract section (lines 42-44), "SH42 delays high fat diet-induced NAFLD/NASH progression from simple steatosis to advanced stages with severe liver inflammation, fibrosis and injury" in the Result section (lines 237-239), and "delays NASH progression to advanced stages with severe liver inflammation and fibrosis" in the Discussion section (lines 255-257).
2. We have modified the last conclusion to "*Treatment with SH42 reduces hepatic crown-like structures, liver collagen content, and plasma alanine transaminase levels in an established NAFLD model*". (lines 223-225)

3. The response to 9 is not satisfying "Re: We used paraffin-embedded liver hematoxylin and eosin (HE) stained slides to visualize lipid droplets (i.e., areas that are not stained by HE), and used ImageJ software to select and quantify those unstained areas." This is not the correct way to do this experiment. The author need to stain with Oil Red O or a similar stain that is specific for lipids.

Re: We thank the reviewer for the suggestion and have now stained liver slides with Oil Red O for lipid content quantification. Consistently, we observed that SH42 treatment reduced liver lipid area positively stained by Oil Red O (-53%, New Fig. 1B and D), but not in LXRα-deficient mice (New Fig. 4A, C). We have updated the details in the sections of Figures (New Fig. 1B and D, New Fig. 4A, C, and New Fig. 6B and D), Results and Materials & Methods.

23rd May 2023

Dear Dr. Wang,

Thank you for the submission of your revised manuscript to EMBO Molecular Medicine. I am pleased to inform you that we will be able to accept your manuscript pending the following final amendments:

1) In the main manuscript file, please do the following:
- Correct/answer the track changes suggested by our data editors by working from the attached document.
- Please add callouts for Fig. 1A, Fig EV3E, Appendix Table S1 and Appendix Table S2. Please make sure that the figures and panels are called out sequentially, currently Fig. 6G is called out before 6F. You refer to the Fig. 3G-I in the text, however Fig. 3 does not have panels G-I, please correct.
- Please rename "Competing Interest" to "Disclosure Statement & Competing Interests". We updated our journal's competing interests policy in January 2022 and request authors to consider both actual and perceived competing interests. Please review the policy https://www.embopress.org/competing-interests and update your competing interests if necessary.
- Author contributions: Please remove it from the manuscript and specify author contributions in our submission system. CRediT has replaced the traditional author contributions section because it offers a systematic machine-readable author contributions format that allows for more effective research assessment. You are encouraged to use the free text boxes beneath each contributing author's name to add specific details on the author's contribution. More information is available in our guide to authors:
https://www.embopress.org/page/journal/17574684/authorguide#authorshipguidelines
2) Appendix: Please move the whole material and methods part to the main manuscript file.
3) Synopsis:
- Synopsis image: Please provide visual abstract as a high-resolution jpeg file 550 px-wide x (250-400)-px high to illustrate your article.
- Please check your synopsis text and image before submission with your revised manuscript. Please be aware that in the proof stage minor corrections only are allowed (e.g., typos).
4) For more information: This space should be used to list relevant web links for further consultation by our readers. Could you identify some relevant ones and provide such information as well? Some examples are patient associations, relevant databases, OMIM/proteins/genes links, author's websites, etc...
5) As part of the EMBO Publications transparent editorial process initiative (see our Editorial at http://embomolmed.embopress.org/content/2/9/329), EMBO Molecular Medicine will publish online a Review Process File (RPF) to accompany accepted manuscripts. This file will be published in conjunction with your paper and will include the anonymous referee reports, your point-by-point response and all pertinent correspondence relating to the manuscript. Let us know whether you agree with the publication of the RPF and as here, if you want to remove or not any figures from it prior to publication. Please note that the Authors checklist will be published at the end of the RPF.
6) Please provide a point-by-point letter INCLUDING my comments as well as the reviewer's reports and your detailed responses (as Word file).

I look forward to reading a new revised version of your manuscript as soon as possible.

Yours sincerely,

Zeljko Durdevic

Zeljko Durdevic
Editor
EMBO Molecular Medicine

*** Instructions to submit your revised manuscript ***

*** PLEASE NOTE *** As part of the EMBO Publications transparent editorial process initiative (see our Editorial at https://www.embopress.org/doi/pdf/10.1002/emmm.201000094), EMBO Molecular Medicine will publish online a Review Process File to accompany accepted manuscripts.

In the event of acceptance, this file will be published in conjunction with your paper and will include the anonymous referee reports, your point-by-point response and all pertinent correspondence relating to the manuscript. If you do NOT want this file to

be published, please inform the editorial office at
contact@embomolmed.org.

When submitting your revised manuscript, please include:

1) a .docx formatted version of the manuscript text (including Figure legends and tables)

2) Separate figure files*

3) supplemental information as Expanded View and/or Appendix. Please carefully check the authors guidelines for formatting Expanded view and Appendix figures and tables at
https://www.embopress.org/page/journal/17574684/authorguide#expandedview

4) a letter INCLUDING the reviewer's reports and your detailed responses to their comments (as Word file).

5) The paper explained: EMBO Molecular Medicine articles are accompanied by a summary of the articles to emphasize the major findings in the paper and their medical implications for the non-specialist reader. Please provide a draft summary of your article highlighting
- the medical issue you are addressing,
- the results obtained and
- their clinical impact.
This may be edited to ensure that readers understand the significance and context of the research.
Please refer to any of our published articles for an example.

6) For more information: There is space at the end of each article to list relevant web links for further consultation by our readers. Could you identify some relevant ones and provide such information as well? Some examples are patient associations, relevant databases, OMIM/proteins/genes links, author's websites, etc...

7) Author contributions: the contribution of every author must be detailed in a separate section.

8) EMBO Molecular Medicine now requires a complete author checklist (https://www.embopress.org/page/journal/17574684/authorguide) to be submitted with all revised manuscripts. Please use the checklist as guideline for the sort of information we need WITHIN the manuscript. The checklist should only be filled with page numbers were the information can be found. This is particularly important for animal reporting, antibody dilutions (missing) and exact values and n that should be indicted instead of a range.

9) Every published paper now includes a 'Synopsis' to further enhance discoverability. Synopses are displayed on the journal webpage and are freely accessible to all readers. They include a short stand first (maximum of 300 characters, including space) as well as 2-5 one sentence bullet points that summarise the paper. Please write the bullet points to summarise the key NEW findings. They should be designed to be complementary to the abstract - i.e. not repeat the same text. We encourage inclusion of key acronyms and quantitative information (maximum of 30 words / bullet point). Please use the passive voice. Please attach these in a separate file or send them by email, we will incorporate them accordingly.

You are also welcome to suggest a striking image or visual abstract to illustrate your article. If you do please provide a jpeg file 550 px-wide x 400-px high.

10) A Conflict of Interest statement should be provided in the main text

11) Please note that we now mandate that all corresponding authors list an ORCID digital identifier. This takes <90 seconds to complete. We encourage all authors to supply an ORCID identifier, which will be linked to their name for unambiguous name identification.

Currently, our records indicate that the ORCID for your account is 0000-0002-0327-0458.

Please click the link below to modify this ORCID:
Link Not Available

12) The system will prompt you to fill in your funding and payment information. This will allow Wiley to send you a quote for the article processing charge (APC) in case of acceptance. This quote takes into account any reduction or fee waivers that you may

be eligible for. Authors do not need to pay any fees before their manuscript is accepted and transferred to our publisher.

*Additional important information regarding Figures

Each figure should be given in a separate file and should have the following resolution:
Graphs 800-1,200 DPI
Photos 400-800 DPI
Colour (only CMYK) 300-400 DPI"

Figures are not edited by the production team. All lettering should be the same size and style; figure panels should be indicated by capital letters (A, B, C etc). Gridlines are not allowed except for log plots. Figures should be numbered in the order of their appearance in the text with Arabic numerals. Each Figure must have a separate legend and a caption is needed for each panel.

*Additional important information regarding figures and illustrations can be found at https://bit.ly/EMBOPressFigurePreparationGuideline. See also figure legend preparation guidelines: https://www.embopress.org/page/journal/17574684/authorguide#figureformat

The system will prompt you to fill in your funding and payment information. This will allow Wiley to send you a quote for the article processing charge (APC) in case of acceptance. This quote takes into account any reduction or fee waivers that you may be eligible for. Authors do not need to pay any fees before their manuscript is accepted and transferred to our publisher.

***** Reviewer's comments *****

Referee #2 (Remarks for Author):

The authors have done the required revisions

Dear Dr. Zeljko Durdevic,

I am delighted to have received the conditional acceptance of our manuscript. We have made the necessary corrections based on your comments. Please review the point-by-point response below.

1) In the main manuscript file, please do the following:
- Correct/answer the track changes suggested by our data editors by working from the attached document.
- Please add callouts for Fig. 1A, Fig EV3E, Appendix Table S1 and Appendix Table S2. Please make sure that the figures and panels are called out sequentially, currently Fig. 6G is called out before 6F.

Re: We have now mentioned Fig.1A, EV3E, Table S1 and S2, and switched Figure 6G and 6F so that the figures are called out sequentially in the manuscript.

You refer to the Fig. 3G-I in the text, however Fig. 3 does not have panels G-I, please correct.

Re: We feel sorry for the mistakes, actually "Fig.3G-I" should be "Fig.2G-I", which has been corrected in the manuscript.

- Please rename "Competing Interest" to "Disclosure Statement & Competing Interests". We updated our journal's competing interests policy in January 2022 and request authors to consider both actual and perceived competing interests. Please review the policy https://www.embopress.org/competing-interests and update your competing interests if necessary.
Re: This has been corrected now.

- Author contributions: Please remove it from the manuscript and specify author contributions in our submission system. CRediT has replaced the traditional author contributions section because it offers a systematic machine-readable author contributions format that allows for more effective research assessment. You are encouraged to use the free text boxes beneath each contributing author's name to add specific details on the author's contribution. More information is available in our guide to authors:
https://www.embopress.org/page/journal/17574684/authorguide#authorshipguidelines

Re: The section has been removed now in the manuscript.

2) Appendix: Please move the whole material and methods part to the main manuscript file.

Re: Done.

3) Synopsis:

- Synopsis image: Please provide visual abstract as a high-resolution jpeg file 550 px-wide x (250-400)-px high to illustrate your article.
- Please check your synopsis text and image before submission with your revised manuscript. Please be aware that in the proof stage minor corrections only are allowed (e.g., typos).

Re: We now have uploaded a new image.

4) For more information: This space should be used to list relevant web links for further consultation by our readers. Could you identify some relevant ones and provide such information as well? Some examples are patient associations, relevant databases, OMIM/proteins/genes links, author's websites, etc...
Re: We do not have any relevant information for this section.

5) As part of the EMBO Publications transparent editorial process initiative (see our Editorial at http://embomolmed.embopress.org/content/2/9/329), EMBO Molecular Medicine will publish online a Review Process File (RPF) to accompany accepted manuscripts. This file will be published in conjunction with your paper and will include the anonymous referee reports, your point-by-point response and all pertinent correspondence relating to the manuscript. Let us know whether you agree with the publication of the RPF and as here, if you want to remove or not any figures from it prior to publication. Please note that the Authors checklist will be published at the end of the RPF.
Re: We fully agree with the publication of the RPF and we do not need remove any figures.

6) Please provide a point-by-point letter INCLUDING my comments as well as the reviewer's reports and your detailed responses (as Word file).

Re: Please check the file.

Yanan Wang

7th Jun 2023

Dear Dr. Wang,

We are pleased to inform you that your manuscript is accepted for publication and is now being sent to our publisher to be included in the next available issue of EMBO Molecular Medicine.

Please read below for additional IMPORTANT information regarding your article, its publication and the production process.

Congratulations on your interesting work,

Zeljko Durdevic

Zeljko Durdevic
Editor
EMBO Molecular Medicine

Follow us on Twitter @EmboMolMed
Sign up for eTOCs at embopress.org/alertsfeeds

*** *** *** IMPORTANT INFORMATION *** *** ***

SPEED OF PUBLICATION
The journal aims for rapid publication of papers, using using the advance online publication "Early View" to expedite the process: A properly copy-edited and formatted version will be published as "Early View" after the proofs have been corrected. Please help the Editors and publisher avoid delays by providing e-mail address(es), telephone and fax numbers at which author(s) can be contacted.

Should you be planning a Press Release on your article, please get in contact with embomolmed@wiley.com as early as possible, in order to coordinate publication and release dates.

LICENSE AND PAYMENT:

All articles published in EMBO Molecular Medicine are fully open access: immediately and freely available to read, download and share.

EMBO Molecular Medicine charges an article processing charge (APC) to cover the publication costs. You, as the corresponding author for this manuscript, should have already received a quote with the article processing fee separately. Please let us know in case this quote has not been received.

Once your article is at Wiley for editorial production you will receive an email from Wiley's Author Services system, which will ask you to log in and will present you with the publication license form for completion. Within the same system the publication fee can be paid by credit card, an invoice, pro forma invoice or purchase order can be requested.

Payment of the publication charge and the signed Open Access Agreement form must be received before the article can be published online.

PROOFS

You will receive the proofs by e-mail approximately 2 weeks after all relevant files have been sent o our Production Office. Please return them within 48 hours and if there should be any problems, please contact the production office at embopressproduction@wiley.com.

Please inform us if there is likely to be any difficulty in reaching you at the above address at that time. Failure to meet our

deadlines may result in a delay of publication.

All further communications concerning your paper proofs should quote reference number EMM-2022-16845-V5 and be directed to the production office at embopressproduction@wiley.com.

Thank you,

Zeljko Durdevic
Editor
EMBO Molecular Medicine

EMBO Press Author Checklist

Corresponding Author Name: Yanan Wang	
Journal Submitted to: EMBO Molecular Medicine	
Manuscript Number: EMM-2022-16845-V2	

USEFUL LINKS FOR COMPLETING THIS FORM
The EMBO Journal - Author Guidelines
EMBO Reports - Author Guidelines
Molecular Systems Biology - Author Guidelines
EMBO Molecular Medicine - Author Guidelines

Reporting Checklist for Life Science Articles (updated January
This checklist is adapted from Materials Design Analysis Reporting (MDAR) Checklist for Authors. MDAR establishes a minimum set of requirements in transparent reporting in the life sciences (see Statement of Task: 10.31222/osf.io/9sm4x). Please follow the journal's guidelines in preparing your
Please note that a copy of this checklist will be published alongside your article.

Abridged guidelines for figures
1. Data
The data shown in figures should satisfy the following conditions:
- the data were obtained and processed according to the field's best practice and are presented to reflect the results of the experiments in an accurate and unbiased manner.
- ideally, figure panels should include only measurements that are directly comparable to each other and obtained with the same assay.
- plots include clearly labeled error bars for independent experiments and sample sizes. Unless justified, error bars should not be shown for technical
- if n<5, the individual data points from each experiment should be plotted. Any statistical test employed should be justified.
- Source Data should be included to report the data underlying figures according to the guidelines set out in the authorship guidelines on Data

2. Captions
Each figure caption should contain the following information, for each panel where they are relevant:
- a specification of the experimental system investigated (eg cell line, species name).
- the assay(s) and method(s) used to carry out the reported observations and measurements.
- an explicit mention of the biological and chemical entity(ies) that are being measured.
- an explicit mention of the biological and chemical entity(ies) that are altered/varied/perturbed in a controlled manner.
- the exact sample size (n) for each experimental group/condition, given as a number, not a range;
- a description of the sample collection allowing the reader to understand whether the samples represent technical or biological replicates (including how many animals, litters, cultures, etc.).
- a statement of how many times the experiment shown was independently replicated in the laboratory.
- definitions of statistical methods and measures:
  - common tests, such as t-test (please specify whether paired vs. unpaired), simple χ2 tests, Wilcoxon and Mann-Whitney tests, can be unambiguously identified by name only, but more complex techniques should be described in the methods section;
  - are tests one-sided or two-sided?
  - are there adjustments for multiple comparisons?
  - exact statistical test results, e.g., P values = x but not P values < x;
  - definition of 'center values' as median or average;
  - definition of error bars as s.d. or s.e.m.

Please complete ALL of the questions below.
Select "Not Applicable" only when the requested information is not relevant for your study.

Materials

Newly Created Materials	Information included in the manuscript?	In which section is the information available? (Reagents and Tools Table, Materials and Methods, Figures, Data Availability Section)
New materials and reagents need to be available; do any restrictions apply?	Not Applicable	

Antibodies	Information included in the manuscript?	In which section is the information available? (Reagents and Tools Table, Materials and Methods, Figures, Data Availability Section)
For **antibodies** provide the following information:<br>- Commercial antibodies: RRID (if possible) or supplier name, catalogue number and or/clone number<br>- Non-commercial: RRID or citation	Yes	Appendix Tables

DNA and RNA sequences	Information included in the manuscript?	In which section is the information available? (Reagents and Tools Table, Materials and Methods, Figures, Data Availability Section)
**Short novel DNA or RNA including primers, probes**: provide the sequences.	Not Applicable	

Cell materials	Information included in the manuscript?	In which section is the information available? (Reagents and Tools Table, Materials and Methods, Figures, Data Availability Section)
**Cell lines:** Provide species information, strain. Provide accession number in repository **OR** supplier name, catalog number, clone number, and/**OR** RRID.	Not Applicable	
**Primary cultures:** Provide species, strain, sex of origin, genetic modification status.	Not Applicable	
Report if the cell lines were recently **authenticated** (e.g., by STR profiling) and tested for mycoplasma contamination.	Not Applicable	

Experimental animals	Information included in the manuscript?	In which section is the information available? (Reagents and Tools Table, Materials and Methods, Figures, Data Availability Section)
**Laboratory animals or Model organisms:** Provide species, strain, sex, age, genetic modification status. Provide accession number in repository **OR** supplier name, catalog number, clone number, **OR** RRID.	Yes	Materials and Methods
**Animal observed in or captured from the field:** Provide species, sex, and age where possible.	Not Applicable	
Please detail **housing and husbandry conditions**.	Yes	Materials and Methods

Plants and microbes	Information included in the manuscript?	In which section is the information available? (Reagents and Tools Table, Materials and Methods, Figures, Data Availability Section)
**Plants:** provide species and strain, ecotype and cultivar where relevant, unique accession number if available, and source (including location for collected wild specimens).	Not Applicable	
**Microbes:** provide species and strain, unique accession number if available, and source.	Not Applicable	

Human research participants	Information included in the manuscript?	In which section is the information available? (Reagents and Tools Table, Materials and Methods, Figures, Data Availability Section)
If collected and within the bounds of privacy constraints report on age, sex and gender or ethnicity for all study participants.	Not Applicable	

Core facilities	Information included in the manuscript?	In which section is the information available? (Reagents and Tools Table, Materials and Methods, Figures, Data Availability Section)
If your work benefited from core facilities, was their service mentioned in the acknowledgments section?	Not Applicable	

Design

Study protocol	Information included in the manuscript?	In which section is the information available? (Reagents and Tools Table, Materials and Methods, Figures, Data Availability Section)

If study protocol has been **pre-registered, provide DOI in the manuscript**. For clinical trials, provide the trial registration number **OR** cite DOI.	Not Applicable	
Report the **clinical trial registration number** (at ClinicalTrials.gov or equivalent), where applicable.	Not Applicable	

Laboratory protocol	Information included in the manuscript?	In which section is the information available? (Reagents and Tools Table, Materials and Methods, Figures, Data Availability Section)
Provide DOI OR other citation details if **external detailed step-by-step protocols** are available.	Not Applicable	

Experimental study design and statistics	Information included in the manuscript?	In which section is the information available? (Reagents and Tools Table, Materials and Methods, Figures, Data Availability Section)
Include a statement about **sample size** estimate even if no statistical methods were used.	Yes	Materials and Methods
Were any steps taken to minimize the effects of subjective bias when allocating animals/samples to treatment (e.g. **randomization procedure**)? If yes, have they been described?	Yes	Materials and Methods
Include a statement about **blinding** even if no blinding was done.	Yes	Materials and Methods
Describe **inclusion/exclusion criteria** if samples or animals were excluded from the analysis. Were the criteria pre-established?<br><br>If sample or data points were omitted from analysis, report if this was due to attrition or intentional exclusion and provide justification.	Yes	Materials and Methods
For every figure, are **statistical tests** justified as appropriate? Do the data meet the assumptions of the tests (e.g., normal distribution)? Describe any methods used to assess it. Is there an estimate of variation within each group of data? Is the variance similar between the groups that are being statistically compared?	Yes	Materials and Methods

Sample definition and in-laboratory replication	Information included in the manuscript?	In which section is the information available? (Reagents and Tools Table, Materials and Methods, Figures, Data Availability Section)
In the figure legends: state number of times the experiment was **replicated** in laboratory.	Yes	Figures
In the figure legends: define whether data describe **technical or biological replicates**.	Yes	Figures

Ethics

Ethics	Information included in the manuscript?	In which section is the information available? (Reagents and Tools Table, Materials and Methods, Figures, Data Availability Section)
Studies involving **human participants**: State details of **authority granting ethics approval** (IRB or equivalent committee(s), provide reference number for approval.	Not Applicable	
Studies involving **human participants**: Include a statement confirming that **informed consent** was obtained from all subjects and that the experiments conformed to the principles set out in the WMA Declaration of Helsinki and the Department of Health and Human Services Belmont Report.	Not Applicable	
Studies involving **human participants:** For publication of **patient photos**, include a statement confirming that consent to publish was obtained.	Not Applicable	
Studies involving experimental **animals**: State details of **authority granting ethics approval** (IRB or equivalent committee(s), provide reference number for approval. Include a statement of compliance with ethical regulations.	Yes	Materials and Methods
Studies involving **specimen and field samples:** State if relevant **permits** obtained, provide details of authority approving study; if none were required, explain why.	Not Applicable	

Dual Use Research of Concern (DURC)	Information included in the manuscript?	In which section is the information available? (Reagents and Tools Table, Materials and Methods, Figures, Data Availability Section)
Could your study fall under dual use research restrictions? Please check biosecurity documents and list of **select agents and toxins** (CDC): https://www.selectagents.gov/sat/list.htm	Not Applicable	
If you used a select agent, is the security level of the lab appropriate and reported in the manuscript?	Not Applicable	
If a study is subject to dual use research of concern regulations, is the name of the **authority granting approval and reference number** for the regulatory approval provided in the manuscript?	Not Applicable	

Reporting

The MDAR framework recommends adoption of discipline-specific guidelines, established and endorsed through community initiatives. Journals have their own policy about requiring specific guidelines and recommendations to complement MDAR.

Adherence to community standards	Information included in the manuscript?	In which section is the information available? (Reagents and Tools Table, Materials and Methods, Figures, Data Availability Section)
State if relevant guidelines or checklists (e.g., **ICMJE, MIBBI, ARRIVE, PRISMA**) have been followed or provided.	Not Applicable	
For **tumor marker prognostic studies,** we recommend that you follow the **REMARK** reporting guidelines (see link list at top right). See author guidelines, under 'Reporting Guidelines'. Please confirm you have followed these guidelines.	Not Applicable	
For **phase II and III randomized controlled trials**, please refer to the **CONSORT** flow diagram (see link list at top right) and submit the CONSORT checklist (see link list at top right) with your submission. See author guidelines, under 'Reporting Guidelines'. Please confirm you have submitted this list.	Not Applicable	

Data Availability

Data availability	Information included in the manuscript?	In which section is the information available? (Reagents and Tools Table, Materials and Methods, Figures, Data Availability Section)
Have **primary datasets** been deposited according to the journal's guidelines (see 'Data Deposition' section) and the respective accession numbers provided in the Data Availability Section?	Not Applicable	
Were **human clinical and genomic datasets** deposited in a public access-controlled repository in accordance to ethical obligations to the patients and to the applicable consent agreement?	Not Applicable	
Are **computational models** that are central and integral to a study available without restrictions in a machine-readable form? Were the relevant accession numbers or links provided?	Not Applicable	
If publicly available data were reused, provide the respective **data citations in the reference list**.	Not Applicable	
